# Multi-omics reveals the regulatory effects of Chinese herbal medicine substrates on secondary metabolite biosynthesis in *Inonotus glomeratus*

**DOI:** 10.3389/fmicb.2025.1658730

**Published:** 2025-11-07

**Authors:** Chengbo Peng, Meng Meng, Xiaolong Yuan, Jiaojun Yu, Boyi Wang, Li Zhong, Yi Wang, Lu Li, Tingwen He, Yuan Zheng

**Affiliations:** 1 College of Forestry, Southwest Forestry University, Kunming, China; 2 Yunnan Key Laboratory of Biodiversity of Gaoligong Mountain, Yunnan Academy of Forestry and Grass-Land, Kunming, China; 3 Hubei Key Laboratory of Economic Forest Germplasm Improvement and Resources Comprehensive Utilization, Huanggang Normal University, Huanggang, China; 4 Yunnan Forestry Technological College, Kunming, China; 5 College of Biological and Food Engineering, Southwest Forestry University, Kunming, China; 6 Edible/Medicinal Fungi Research Innovation Team, Modern Industry School of Edible-Fungi, Southwest Forestry University, Kunming, China; 7 Forest Resources Exploitation and Utilization Engineering Research Center for Grand Health of Yunnan Provincial Universities, Southwest Forestry University, Kunming, China

**Keywords:** *Inonotus glomeratus*, genome sequence, biological synthesis gene cluster, metabolomics analysis, transcriptome sequencing, transcriptional regulation, bioactive compound

## Abstract

**Introduction:**

The fungal genus *Inonotus* is renowned for its medicinal properties, including antioxidant and anti-tumor activities, which are largely attributed to its rich repertoire of terpenoid and polyphenolic secondary metabolites. This study aimed to investigate how different Chinese herbal medicine powders used as culture media influence the secondary metabolite profile of *Inonotus glomeratus*.

**Methods:**

This study employed a multi-omics approach, utilizing Illumina and Nanopore sequencing to assemble a high-quality genome for *I. glomeratus*. The fungus was cultivated on media containing powders from *Polygonum multiflorum*, *Coix lacryma-jobi*, *Pisum sativum* flour, *Salvia miltiorrhiza*, *Panax ginseng*, and *Astragalus membranaceus*. Subsequent integrated metabolomic and transcriptomic analyses were conducted to profile secondary metabolite production and identify key biosynthetic genes.

**Results:**

Experimental results show that the assembled *I. glomeratus* genome was 38.68 Mb in size, consisting of 23 scaffolds with a GC content of 47.98%. The genome annotation process identified 67 transcription factors, four polyketide synthases (PKSs), one non-ribosomal peptide synthase, and 11 terpenoid synthases (TPSs). Multi-omics analysis revealed that terpenoid biosynthesis in *I. glomeratus* was significantly enhanced in DS and RS media. Betulin and betulinic acid exhibited the most dramatic increases in RS medium, reaching 4,658–9,275-fold and 4–503-fold higher concentrations, respectively. The transcriptome results showed that the expression of enzymes such as *IgAACT*, *IgHMGR*, *IgSQS*, *IgSES*, and *IgTPS9* was significantly higher in the RS medium than in the other treatment groups. Integrated metabolomic and transcriptomic analyses suggested that *IgPKS1* participates in orsellinic acid biosynthesis. while *IgPKS2* is likely involved in naringenin biosynthesis. Additionally, *IgTPS9* was associated with betulinic acid biosynthesis, and *IgTPS10* contributed to tetracyclic sesquiterpene B-type triterpene formation. Co-expression network analysis and transcription factor binding site prediction indicated that *IgMYB3* may regulates *IgPKS1* expression, whereas *IgHSF1* may simultaneously modulate *IgTPS4* and *IgTPS6*.

**Discussion:**

This study provides novel insights into the regulatory mechanisms governing secondary metabolite production in *I. glomeratus*. These findings offer a foundation for targeted metabolic engineering and the optimized production of valuable compounds.

## 1 Introduction

The prevalence of fungal resources in our country is well-documented, and the utilization of fungal medicines has become a significant component of the traditional Chinese medicine industry (Guo et al., 2021). In recent years, the development of modern medical technology has led to significant progress in the understanding of the active components and pharmacological effects of various medicinal fungi. This has resulted in a growing interest among scholars worldwide in the medical value and health benefits of these organisms (Łysakowska et al., 2023; Mateo et al., 2015). Fungal secondary metabolites serve as a vital source of lead compounds in the domain of pharmaceutical research and development. In addition to this primary function, these compounds play a crucial role in stress response mechanisms. These metabolites exhibit a variety of structural frameworks, including polyketides, terpenoids, non-ribosomal peptides, and alkaloids (Vassaux et al., 2019) Recent studies have demonstrated that these bioactive compounds possess a variety of physiological activities in humans, including immunomodulatory (Suabjakyong et al., 2015), antitumor (Zhang et al., 2019), hepatoprotective, hypoglycemic (Liu et al., 2019), antioxidant (Zorov et al., 2014), antimicrobial (Angelini et al., 2019), and antiviral (Chen et al., 2006) properties. Consequently, these metabolites hold significant promise for medical applications (Avalos and Limón, 2021).

**Figure 1:**
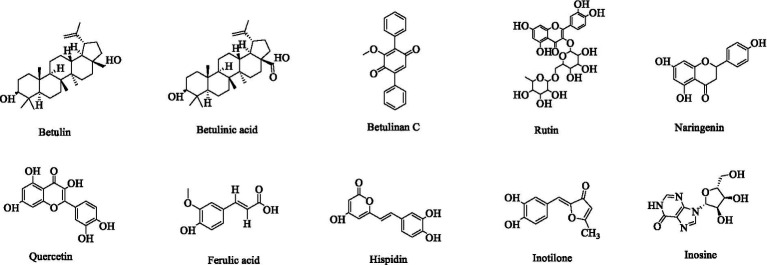
Presents a structural diagram of several compounds isolated from *Inonotus*.

The Hymenochaetaceae family, which belongs to the Basidiomycota division, includes the medicinally significant genus *Inonotus* (Joshi et al., 2021; Cannon and Kirk, 2007). Recent phytochemical investigations have identified diverse bioactive compounds from *Inonotus* species, including polyphenols (Shahidi and Ambigaipalan, 2015), polysaccharides (Duru et al., 2019), flavonoids (Mathesius, 2018), terpenoids (Kim et al., 2020; Alzand et al., 2018), and steroids (Li and Bao, 2022). These secondary metabolites exhibit novel structural features and significant biological activities (Figure 1). It is noteworthy that triterpenoids, including betulin, betulinic acid, and betulinan C, have been isolated from the mycelia of *Inonotus obliquus* (Raal et al., 2024). Betulin has been demonstrated to possess a wide range of pharmacological properties, including antiviral, antibacterial, and antitumor activities (Amiri et al., 2020). Betulinic acid has emerged as a particularly promising compound due to its multifaceted bioactivities, exhibiting anti-inflammatory, anti-HIV (Li et al., 2016), anticancer (especially against melanoma) (Zhang et al., 2020), antibacterial, anthelmintic (Fontanay et al., 2008), and antitumor effects (Bildziukevich et al., 2019; Lee et al., 2019). Notably, betulinic acid exhibits selective toxicity, demonstrating minimal impact on normal cells (Zuco et al., 2002), a property that enhances its clinical viability. The pharmacological properties of betulinic acid are such that it is a valuable candidate for anti-HIV therapies and oncological treatments, warranting further preclinical and clinical investigation (Ali-Seyed et al., 2016). Betulinan C has demonstrated significant biofilm inhibitory activity (Firke et al., 2015). Flavonoid constituents such as rutin, naringenin, and quercetin have been identified in *Inonotus* species (Nakajima et al., 2007; Wang et al., 2021), exhibiting potent anti-inflammatory and antioxidant properties (Atoki et al., 2024; Wang G. et al., 2022; Hassani and Esmaeili, 2024). Rutin has also been shown to possess notable anticancer activity (Imani et al., 2021). Other bioactive compounds include ferulic acid, a phenolic compound with antioxidant capacity isolated from *I. obliquus* (Xu et al., 2015), and inosine, an anti-inflammatory agent obtained from *I. obliquus* fruiting body methanol extracts (Tao et al., 2016). In addition, two polyphenolic compounds, hispidin and inotilone, were isolated from *I. hispidus* fruiting body ethanol extracts (Hou et al., 2019). In addition to its antioxidant activity (Palkina et al., 2021), hispidin has demonstrated potential hypoglycemic activity (Lee et al., 2008). Inotilone has been shown to possess immunomodulatory, anticancer, and antiviral effects (Wangun et al., 2006).

Solid-state fermentation is a microbial cultivation technique that employs solid substrates, demonstrating significant potential for the production of bioactive metabolites from medicinal fungi (Berovic and Zhong, 2023). This approach effectively integrates modern biotechnological principles with traditional Chinese medicinal processing, offering distinct advantages such as enhanced efficacy, reduced toxicity, improved pharmaceutical properties, and increased yields of active constituents (Li et al., 2020). Recently, the strategy of using powdered Chinese herbal materials as fermentation substrates to direct fungal growth and metabolism has attracted considerable research interest (Wu et al., 2013). Herbs including *Polygonum multiflorum*, *Coix lacryma-jobi*, *Pisum sativum* flour, *Salvia miltiorrhiza*, *Panax ginseng*, and *Astragalus membranaceus* are abundant in polysaccharides, flavonoids, saponins, and other essential nutrients, thereby providing comprehensive nutritional support for fungal development. Evidence suggests that the distinct nutritional composition and physical structure of various herbal substrates can modulate fungal enzyme systems and secondary metabolic pathways, potentially leading to the biosynthesis of novel bioactive compounds (Zhang X. et al., 2023). Nevertheless, systematic investigations into the impact of single or composite Chinese herbal powders on fungal fermentation behavior and the underlying regulatory mechanisms are still insufficient. Consequently, a deeper exploration of the applicability and functional regulatory roles of these herbal substrates is of considerable importance.

Terpenoids, the most abundant class of secondary metabolites in nature, have garnered significant research interest due to their structural diversity and broad spectrum of biological activities. Within their biosynthetic pathways, terpene synthases (TPS) serve as pivotal enzymes that govern carbon skeleton formation and structural diversification. These enzymes catalyze the conversion of universal precursors—isopentenyl pyrophosphate (IPP) and dimethylallyl pyrophosphate (DMAPP)—into various terpenoid scaffolds (Wu et al., 2016). Subsequent modifications by cytochrome P450 enzymes, including oxidation, hydroxylation, and methylation reactions, further enhance structural complexity and biological functionality. Notably, the genetic diversity of the TPS family directly correlates with terpenoid structural variability, which in turn influences functional diversity (Luo et al., 2024). Based on their carbocation formation mechanisms, TPS enzymes are classified into three categories (Christianson, 2017). Class I terpenoid cyclase includes monoterpene, sesquiterpene, and diterpene cyclase, which remove the pyrophosphate group of the substrate by ionization of metal ions (Mg^2+^, Mn^2+^); class II terpenoid cyclase includes diterpene, triterpene, and sesquiterpene cyclase, which remove the pyrophosphate group of the substrate by protonation of a carbon–carbon double bond formed by aspartate side chains. The catalytic structural domains of Class I terpenoid cyclase are an aspartic acid-rich region (DDXXD) and NSE/DTE, which mainly remove the pyrophosphate group of the substrate by ionization; the catalytic structural domains of class II terpenoid cyclase are also an aspartate-rich region (DXDD), which removes the pyrophosphate group of the substrate mainly through protonation (Oldfield and Lin, 2012).

The biosynthesis of polyketide compounds is primarily catalyzed by polyketide synthase (PKS) and post-modifying enzymes. PKS, the pivotal enzyme in the synthesis of polyketide compounds, catalyzes the sequential decarboxylation and recurrent condensation of multiple thiooctanoyl coenzyme A molecules to generate polyketide compounds. A taxonomic classification of PKS is possible based on its protein structure and catalytic mechanism, resulting in three distinct types: type I, type II, and type III (Cox, 2007). Type I PKS is further subdivided into type I modular PKS and type I iterative PKS. The majority of fungal PKSs are classified as type I. The fundamental structural domains of PKS are comprised of *β*-ketolipoyl synthase (KS), acyltransferase (AT), and acyl carrier protein (ACP) components (Buyachuihan et al., 2024; Musiol-Kroll and Wohlleben, 2020; Sigrist et al., 2020). Depending on the extent to which the β-keto group is reduced, PKSs can be further categorized into three subgroups: highly reducing PKS (HR-PKS), partially reducing PKS (PR-PKS), and non-reducing PKS (NR-PKS) (Herbst et al., 2018). In addition to other organisms, the biosynthesis of the flavonoid naringenin is typically catalyzed by a combination of type III PKS, chalcone isomerase (CHI), and flavonoid synthase (FNS) (Pluskal et al., 2019; Nabavi et al., 2020). However, recent studies suggest that fungi may synthesize flavonoids through unique metabolic pathways, a finding that challenges conventional knowledge. Genomic studies have revealed the presence of gene/protein sequences associated with flavonoid biosynthesis in fungal genomes (Mohanta, 2020). This finding suggests that these organisms may possess a distinct synthesis mechanism compared to plants. Further studies revealed that a fungal non-ribosomal peptide synthase-polyketide synthase (NRPS-PKS) heterotrimeric enzyme, FnsA (structural domain composition: A-T-KS-AT-DH-KR-ACP-TE), was able to catalyze the synthesis of naringenin using either p-coumaric acid (p-CA) or p-hydroxybenzoic acid (p-HBA) as substrates (Zhang et al., 2022). Furthermore, through a self-resistant gene-directed strategy, the researchers identified a biosynthetic gene cluster for chlorflavonin, a fungal flavonoid with acetolactate synthase inhibitory activity, and a synthetic pathway that reveals a novel mechanism for the biosynthesis of fungal flavonoids. The core step of the pathway is catalyzed by NRPS-PKS, which facilitates the generation of the key precursor chalcone. Subsequently, a novel CHI converts the chalcone to a flavonoid via a histidine-mediated oxa-Michael addition reaction. Ultimately, a flavin mononucleotide (FMN)-dependent oxidoreductase (FNS) catalyzes flavonoid desaturation to form flavonoids (Zhang W. et al., 2023). These findings contribute to the expansion of knowledge regarding the flavonoid biosynthetic pathway.

In this study, we performed comprehensive whole-genome sequencing and functional annotation of *I. glomeratus* strains using a hybrid sequencing approach that combined second-generation Illumina NovaSeq and third-generation Oxford Nanopore Technologies platforms. The present study builds upon the established genomic foundation by conducting integrated multi-omics analyses to characterize PKS and TPS genes associated with secondary metabolism. This involved correlating genomic data with LC-MS/MS-based metabolite profiling and RNA-seq transcriptomic data across six distinct substrate culture conditions. Furthermore, leveraging the complete genome assembly, we systematically identified and computationally analyzed transcription factor families in *I. glomeratus*, with particular focus on their potential regulatory roles in PKS and TPS gene expression. The findings of this study offer significant molecular insights into secondary metabolic pathways, their bioactive products, and transcriptional regulatory networks in this medicinally important fungal species.

## 2 Materials and methods

### 2.1 Fungus strain

The *I. glomeratus* strain is conserved in the Yunnan Province Gaoligong Mountain Biodiversity Key Laboratory, Yunnan Academy of Forestry and Grassland Sciences, Kunming.

### 2.2 Fungal cultivation

The preserved *I. glomeratus* strain was retrieved from a −80 °C freezer. Following a thawing process on ice, the sample was inoculated onto Potato Dextrose Agar (PDA) medium. The inoculated medium was then subjected to incubation in conditions of darkness at 26 °C with 50–80% relative humidity for a period of 7 days. Thereafter, it was stored at a temperature of 4 °C. During fungal culturing, 0.5 cm^2^ mycelial plugs were aseptically collected from the edge of *I. glomeratus* colonies and uniformly inoculated onto different media. These samples were subsequently cultivated in a 26 °C constant-temperature incubator. The liquid medium formulation for the genome sequencing strain is malt/yeast extract medium. Cultivate at 28 °C on a dark rotating shaker at 150 rpm. Harvest the fungal mycelium after 10 days. The solid medium formulation for sequencing strains of metabolomics and transcriptomics is as follows: HSW (20 g processed *Polygonum multiflorum* powder + 15 mL MM medium), YM (20 g processed coix seed powder + 15 mL MM medium), WDF (20 g *Pisum sativum* flour + 15 mL MM medium), DS (20 g *Salvia miltiorrhiza* powder + 15 mL MM medium), RS (20 g *Panax ginseng* powder + 15 mL MM medium), and HQ (20 g *Astragalus membranaceus* powder + 15 mL MM medium), 15 mL MM medium (containing 6 g/L sodium nitrate, 0.52 g/L potassium chloride, 0.52 g/L magnesium sulfate, and 1.52 g/L potassium dihydrogen phosphate). The culture medium should be transferred into tissue culture flasks, inoculated, and then incubated at 26 °C in a constant-temperature incubator for 15 days, after which the fungal mycelia were harvested. All media were sterilized via autoclaving at 121 °C for 20 min.

### 2.3 Genome sequencing and assembly

Following a 10-day period of liquid culture, genomic DNA was extracted from the mycelium of *I. glomeratus*. The high-throughput sequencing was conducted by Shanghai Personal Biotechnology Co., Ltd. A whole-genome shotgun (WGS) strategy was employed to construct libraries with varying insert sizes, which were subsequently subjected to paired-end (PE) sequencing using the Illumina NovaSeq platform. The raw sequencing data were *de novo* assembled using Falcon and CANU to generate contigs and scaffolds. Subsequently, the assembled sequences were polished using Pilon v1.18 (Walker et al., 2014) for error correction.

### 2.4 Gene prediction and annotation

Using the MAKER (version: 2.31.10) software, the gene sets predicted by various methods were integrated. Firstly, the software RepeatMasker (version: open-4.0.9) was used to annotate repeats based on the RepBase library^1^; then, the software RepeatModeler (version: open-1.0.11) was used to build a library based on the de novo prediction of its sequence features; finally, all the repeat prediction results were combined and predicted. Then, we used RepeatModeler (version: open-1.0.11) to build a library based on the de novo prediction of our sequence features, and we also used RepeatMasker (version: open-4.0.9) to compare and predict the repeat sequences; finally, all the results of the repeat prediction were merged and made unredundant to obtain the final genome repeat sequence set. The tRNAscan-SE (version: 1.23) was used for tRNA prediction, the rRNA database for rRNA prediction, and INFERNAL (version: 1.1.2) based on the Rfam database to find ncRNA sequences in the genome. BLAST searches of non-redundant (NR) protein sequences from the NCBI, Kyoto Encyclopedia of Genes and Genomes (KEGG), Gene Ontology (GO), and Clusters of Orthologous Groups (COG/KOG) were performed to annotate the gene products.

### 2.5 Metabolomics analysis

#### 2.5.1 Metabolite extraction

Weigh 60 mg of the sample into a 2 mL centrifuge tube. Add 500 μL of pre-chilled methanol (−20 °C) and 500 μL of cold water (4 °C), then add 100 mg of glass beads and vortex for 30 s. Place the centrifuge tube into a 2 mL adapter, immerse it in liquid nitrogen for 5 min, then remove and allow it to thaw at room temperature. Mount the centrifuge tube in a grinder using a 2 mL adapter and oscillate at 55 Hz for 2 min, performing two grinding cycles. Centrifuge the tube at 12,000 rpm for 10 min at 4 °C. The supernatant is collected, concentrated, and dried by centrifugation. Reconstitute the dried sample in 300 μL of 50% aqueous methanol solution (1:1, 4 °C) containing 2-chlorophenylalanine (4 ppm). Filter through a 0.22 μm membrane to obtain the final sample for analysis. The prepared sample is then subjected to LC-MS analysis.

#### 2.5.2 Chromatographic conditions

Chromatographic analysis was performed according to the method described by Qiao et al. using an Agilent 1,260 Infinity LC system (Agilent Technologies, Santa Clara, CA, United States) coupled to an Orbitrap Elite-ETD mass spectrometer (Thermo Fisher Scientific, Waltham, MA, United States). Separation was achieved on an ACQUITY UPLC BEH C18 column (1.7 μm, 2.1 × 50 mm; Waters, Milford, MA, United States) with a mobile phase consisting of (A) 0.1% acetic acid in water and (B) acetonitrile. The flow rate was maintained at 0.3 mL/min with the following gradient program: 0 min, 30% B; 3–5 min, 53% B; 12 min, 90% B; 15–18 min, 95% B. The injection volume was 5 μL.

#### 2.5.3 Mass spectrometry conditions

The instrument was operated using an electrospray ionization (ESI) source in both positive and negative ion modes. The spray voltage was set to 3.50 kV for positive mode and 2.50 kV for negative mode. The sheath gas and auxiliary gas were set at 30 and 10 arbitrary units (arb), respectively. The capillary temperature was maintained at 325 °C. Full-scan acquisition was performed at a resolution of 70,000 over an *m*/*z* range of 81–1,000. Fragmentation was conducted using higher-energy collisional dissociation (HCD) with a collision energy of 30 eV. Dynamic exclusion was applied to eliminate redundant MS/MS data.

#### 2.5.4 Data processing and statistical analysis

The raw data were converted to mzXML format using ProteoWizard software (v3.0.8789). Peak detection, filtering, and alignment were performed using the XCMS package in R (v3.3.2), resulting in a data matrix comprising the mass-to-charge ratio (*m*/*z*), retention time (rt), and peak intensity. After data processing, metabolite identification was conducted by querying several databases, including the Human Metabolome Database (HMDB),^2^ METLIN, (see text footnote 2) MassBank,^3^ LipidMaps,^4^ and mzCloud.^5^ Multivariate statistical analyses, including principal component analysis (PCA) and partial least squares discriminant analysis (PLS-DA), were performed to visualize metabolic differences between experimental groups. Metabolites with significant variation were screened based on variable importance in projection (VIP >1) and *p*-value (*p* < 0.05). The metabolite content of mycelia cultured on HSW, YM, WDF, DS, RS, and HQ was analyzed using one-way ANOVA followed by Tukey’s *post hoc* test. Each treatment included three biological replicates.

### 2.6 Secondary metabolite biosynthesis gene analysis

The secondary metabolite biosynthesis genes of *I. vitis*, *I. hispidus*, and *I. obliquus* were predicted using the antiSMASH online tool.^6^ Gene structures were annotated via FGENESH,^7^ while NRPS/PKS domain-containing gene clusters were identified using the NRPS/PKS analysis platform.^8^ Additionally, protein domains were analyzed through NCBI BLAST^9^ to detect contigs harboring NRPS, PKS, and TPS genes.

### 2.7 Cluster analysis

Known PKS and TPS protein sequences were retrieved from NCBI and aligned using the Clustal W program in MEGA 5.0 software. Subsequently, these sequences were compared with the protein sequences obtained in this study. A phylogenetic tree was constructed using the IQ-TREE web server^10^ with the maximum likelihood (ML) method for rapid and accurate inference. The analysis was performed using default parameters with 1,000 bootstrap replicates to ensure robust tree topology.

### 2.8 Prediction of TPS proteins

InterProScan v5.44-79.0 (Jones et al., 2014) was employed to identify terpene synthase (TPS) proteins in *I. glomeratus* by analyzing conserved structural domains, including farnesene synthase (TRI5, IPR024652), pentenyl synthase (Pents, IPR034686), isopentenyltransferase (PTase, IPR039653), squalene-hopene cyclase (lanosterol synthase, IPR018333), and squalene synthase (SQS, IPR002060). The candidate gene sequences obtained were validated through comparison with the NCBI protein database. Subsequently, multiple sequence alignments were conducted using DNAMAN software (version 6.0) to further characterize conserved domains.

### 2.9 Identification and analysis of transcription factors

The protein domains of MYB (PF00249), bHLH (PF00010), bZIP (PF00170), FTD (PF04082), TFIIB (PF08613), HSF (PF00447), C2H2-zinc finger (PF00096), CCCH-zinc finger (PF00642), C6-Zinc (PF00172), Forkhead (PF00250), Ankyrin (PF12796), and HMG (PF00505) transcription factors were retrieved from the InterProScan database. Global alignment and screening of protein sequences in the *I. glomeratus* fungal genome were conducted using HMMER software (version 3.4), with an E-value threshold set to <10^−5^. Sequences shorter than 100 amino acids were manually excluded.

### 2.10 Transcriptome sequencing and differential gene expression analysis

Utilizing next-generation sequencing (NGS) technology on the Illumina HiSeq platform with a paired-end sequencing approach, we conducted the sequencing of samples cultivated under six distinct substrate conditions. After RNA-seq, the first step was to analyze raw reads in fastq format for quality control, filtering out some adapter sequences and low-quality Reads to obtain clean reads. The Q20 (%) and Q30 (%) content of the clean reads were calculated and data quality assessment performed on the clean reads. Differentially expressed genes (DEGs) were analyzed using HISAT2 (v2.1.0) software to align the clean data to the reference genome. Based on FPKM values, DEseq2 was used for differential screening analysis. GO functional enrichment analysis and KEGG pathway enrichment analysis were performed on DEGs. The criteria for selecting DEGs were as follows: expression difference fold change |log2FoldChange| >1, significance *p*-value <0.05. The significantly enriched GO terms and KEGG pathways of the DEGs were identified, and their main biological functions were determined. Based on the sequence numbers of PKS, TPS, and TFs in the whole-genome data of *I. glomeratus*, their gene quantification indicators (FPKM values) were searched in the transcriptomic data, and TBtools software (version 2.056) was used to draw an interactive heatmap to analyze the expression levels of the target genes.

### 2.11 Correlation analysis between the metabolome and transcriptome data

Metabolomics and transcriptomics were integrated using Pearson correlation coefficients (PCCs). All data were log-transformed prior to analysis, and the correlation between metabolomics and transcriptomics was assessed using the core function in the stats R package (version 4.1.0), with a PCC threshold of 0.95. A nine-quadrant plot was generated using the plyr and ggplot2 R packages. Draw the correlation network diagram using the igraph package in R. Draw the correlation and chord diagram using the circlize package in R. Draw the cluster correlation heatmap using the ComplexHeatmap package in R.

### 2.12 Real-time quantitative fluorescence PCR

Genes exhibiting similar expression patterns across different culture matrix conditions were selected, including *IgTFs*, *IgPKS*, and *IgTPS* genes, such as *IgPKS1*, *IgPKS2*, *IgTPS3*, *IgTPS9*, *IgTPS11*, *IgMYB3*, *IgHMG1*, *IgAnkyrin6*, and *IgbHLH2*. Gene-specific primers were designed using Primer Premier 5.0 software to assess their expression levels. Quantitative real-time PCR (qRT-PCR) analyses were conducted in triplicate for each sample. Tubulin alpha was used for the internal control. The sequence information for all primers is detailed in Supplementary Table S1. The PCR reaction mixture had a total volume of 20 μL, consisting of 10 μL of PCR master mix, 1 μL of DNA/cDNA template, 2 μL of primers, and 7 μL of deionized water. The PCR conditions comprised an initial denaturation step at 94 °C for 2 min., followed by 40 amplification cycles (94 °C for 15 s, 65 °C for 15 s, and 72 °C for 45 s), and a final extension at 72 °C for 10 min.

### 2.13 Prediction of transcription factor binding sites

Using the whole-genome and transcriptome data of *I. glomeratus*, DNA sequences 2000 bp upstream of the start codon of PKS and TPS genes that exhibit similar expression patterns to TFs were extracted with TBtools software version 2.056. Potential binding sites for TFs to their co-expressed gene promoter regions were subsequently predicted using the JASPAR online tool, with a confidence level set at 90% (Fornes et al., 2020).

## 3 Results

### 3.1 Basic features of the *Inonotus glomeratus* fungal genome

#### 3.1.1 Genome annotation

A total of 38,374,347 high-quality reads were obtained through Illumina sequencing, which were subsequently assembled into a high-quality genome. The assembled genome had a size of 38.68 Mb, comprising 23 scaffolds with an N50 of 2,085,074 bp and a GC content of 47.98%. Gene prediction identified 8,944 protein-coding genes, with the longest contig measuring 4,540,672 bp, an average overlap cluster length of 1,668,449 bp, and the longest overlap cluster spanning 4.54 Mb (Table 1). tRNA genes were predicted using tRNAscan-SE (v1.3.1), rRNA genes were identified via RNAmmer 1.2 (Lagesen et al., 2007), and other non-coding RNAs were primarily detected through Rfam comparison (Griffiths-Jones, 2005). This analysis revealed 84 tRNA secondary structures, 15 rRNA genes, and 12 snRNA genes using tRNAscan, RNAmmer, and rfam_scan, respectively.

**Table 1:** Genome assembly and characterization of *I. glomeratus*.

Item	Value	Item	Value
Total length (bp)	38,374,347	Scaffolds	23
Max length (bp)	4,540,672	Contigs N50 (bp)	2,085,074
GC content (%)	47.98	Scaffolds N50 (bp)	2,085,074
Gene number	8,944	Contigs N90 (bp)	1,352,901
Contigs	23	Scaffolds N90 (bp)	1,352,901

#### 3.1.2 Genome annotation of *Inonotus glomeratus*

**Figure 2:**
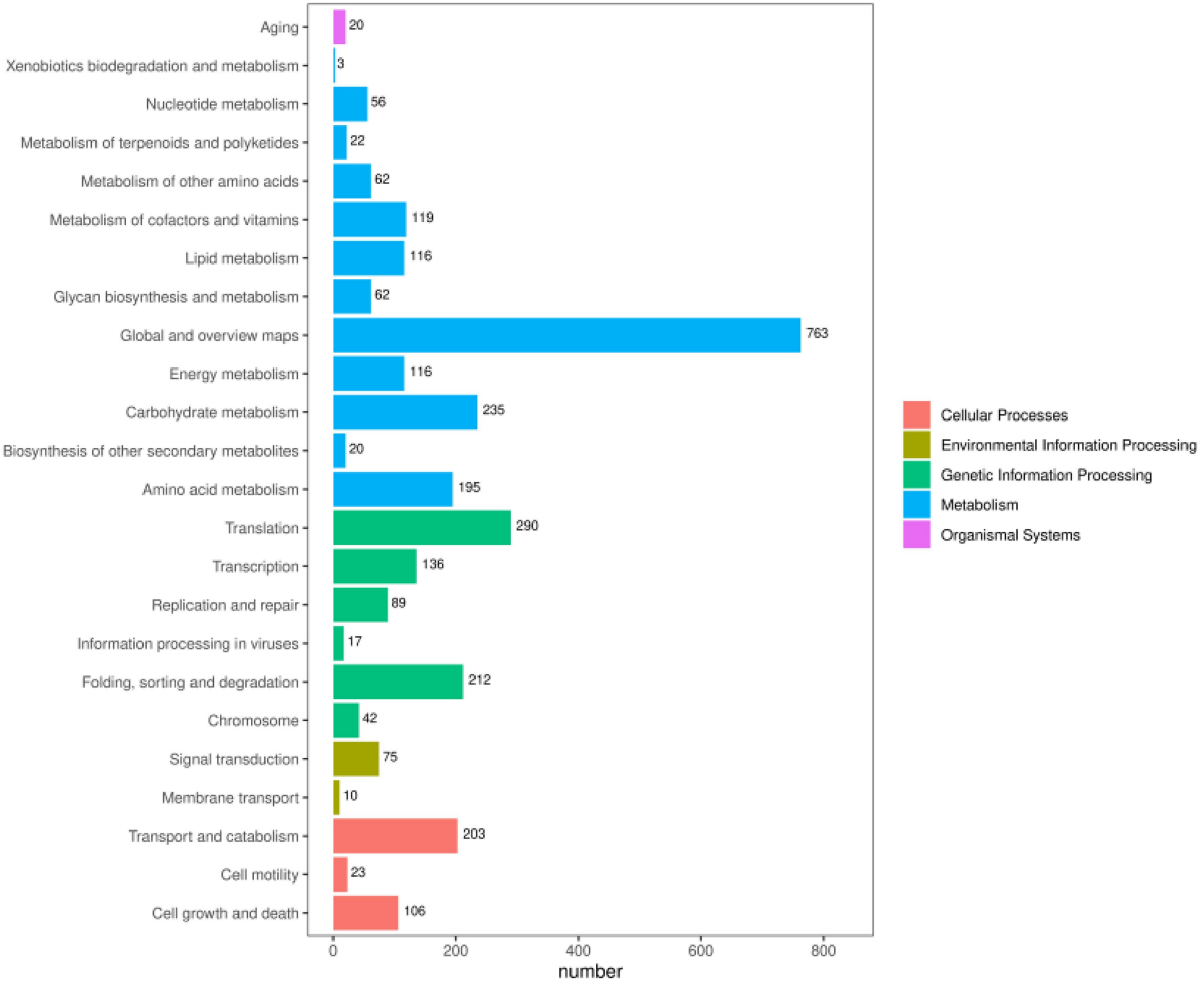
KEGG functional annotation of the protein encoded by the *I. glomeratus* gene. The horizontal axis represents the number of annotated genes under each pathway category, the vertical axis represents the pathway category, and the different colors represent the large category to which they belong.

The 8,944 non-redundant genes predicted in the *I. glomeratus* fungal genome were functionally annotated using multiple databases, including NR, KEGG, GO, EggNOG, and Pfam, yielding varying results. The annotation success rates were 92.67% in the NCBI Nr database (15,441 genes), 40.93% in the KEGG database (6,819 genes), 40.64% in the GO database (6,771 genes), and 85.06% in the EggNOG database (14,173 genes) (Table 2). KEGG analysis revealed that *I. glomeratus* genes are primarily involved in metabolism, genetic information processing, cellular processes, environmental information processing, and biological systems. Among the 24 subcategories, the “global and overview maps” pathway (763 genes) was the most abundant, followed by “translation” (290 genes) and “carbohydrate metabolism” (235 genes) (Figure 2). According to the EggNOG database, most predicted genes were functionally associated with “unknown function” (252 genes), “post-translational modification, protein turnover, molecular chaperones,” and “carbohydrate transport and metabolism” (Supplementary Figure S1). Notably, the enrichment of post-translational modifications and carbohydrate metabolism suggests enhanced regulatory protein activity and energy metabolism. GO annotation provided further insight into the biological significance of these genes. The analysis categorized annotated genes into three functional groups: biological processes, cellular components, and molecular functions. In biological processes, the predominant categories were “translation” (102 genes), “protein transport” (94 genes), and “carbohydrate metabolic process” (134 genes). Within cellular components, genes were primarily associated with “membrane-integrated components” (134 genes), “nucleus” (474 genes), and “cytoplasm” (276 genes). Molecular functions were dominated by “ATP binding” (666 genes) and “metal ion binding” (369 genes) (Supplementary Figure S1). These findings indicate a substantial number of genes related to cellular structure, metabolic efficiency, and regulatory functions, which may contribute to the survival and adaptability of *I. glomeratus*.

**Table 2:** Functional annotation of *I. glomeratus* genome.

Item	Count	Percentage (%)
NR	15,441	92.67%
Uniprot	7,957	88.96%
KEGG	6,819	40.93%
EggNOG	14,173	85.06%
GO	6,771	40.64%
Pfam	5,752	64.31%

#### 3.1.3 Additional annotation of *Inonotus glomeratus*

##### 3.1.3.1 Carbohydrate genes

The carbohydrate-active enzymes (CAZy) family represents one of the most critical gene families in fungal genomes, playing a pivotal role in fungal metabolism (Garron and Henrissat, 2019). In this study, 297 genes encoding CAZy enzymes were identified in the *I. glomeratus* genome (Table 3), comprising 154 glycoside hydrolases (GHs), 17 carbohydrate esterases (CEs), 57 auxiliary activities (AAs), 4 carbohydrate-binding modules (CBMs), 53 glycosyltransferases (GTs), and 12 polysaccharide lyases (PLs). GHs serve as the primary enzymes responsible for hydrolyzing glycosidic bonds in cellulose and hemicellulose, whereas AAs frequently function synergistically with GHs. Proteins harboring domains from the GH5, GH6, GH7, GH8, GH9, and GH12 families predominantly target cellulose, while the GH18 and GH19 families act on chitin substrates. Additionally, families AA1–AA3, AA5–AA9, and AA14 exhibit activity toward cellulose and hemicellulose (Sista Kameshwar and Qin, 2018). The predominance of GHs and AAs in *I. glomeratus* suggests a high lignocellulose degradation capacity, reflecting its efficient energy acquisition potential.

**Table 3:** CAZy functional classification of *I. glomeratus*.

Description	Class	Number	Content (%)
Glycoside hydrolases	GHs	154	51.85
Glycosy transferases	GTs	53	17.85
Polysaccharide lyases	PLs	12	4.04
Carbohydrate esterases	CEs	17	5.72
Auxiliary activities	AAs	57	19.19
Carbohydrate-binding modules	CBMs	4	1.35

##### 3.1.3.2 Transport classification database

**Figure 3:**
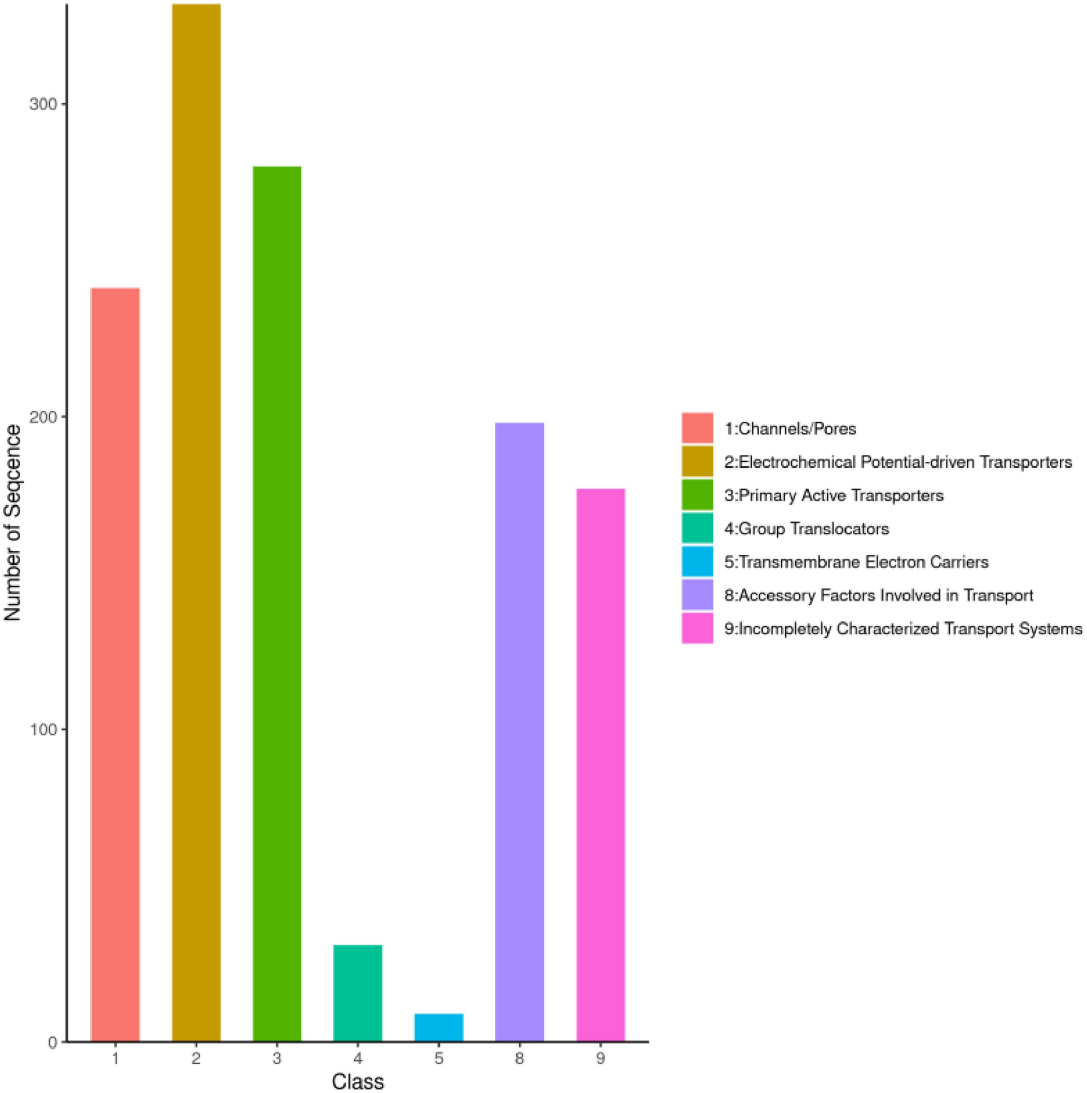
TCDB functional classification of *I. glomeratus*. The horizontal axis represents the annotated gene category, and the vertical axis represents the number of genes.

The Transporter Classification Database (TCDB) comprises over 10,000 non-redundant transport systems, categorized into 1,322 transport protein families. As a freely accessible reference resource, TCDB provides comprehensive information on the structure, function, mechanism, evolution, and disease associations of transport proteins across diverse organisms (Cragg et al., 2015). Analysis revealed that the strain harbors a diverse array of cell membrane transport proteins, including 332 electrochemical potential-driven transporters, 280 primary active transporters, 241 channels/pores, 198 transport cofactors, 177 incompletely characterized transport systems, 31 group translocases, and 9 transmembrane electron carriers (Figure 3). These findings suggest that the strain exhibits significant functional diversity and robust material transport capabilities.

### 3.2 Comparative analysis of genomic characteristics of *Inonotus*

The genome of *I. glomeratus* was analyzed and compared with those of seven other fungi within the same genus (Supplementary Table S2). Among these, *I. glomeratus* exhibited the largest genome size (38.68 Mb), whereas *I. hispidus* possessed the smallest (34 Mb). Owing to variations in sequencing platforms and assembly techniques, the number of scaffolds differed significantly across strains. *I. vitis* contained the highest number of scaffolds (Herbst et al., 2018), while *I. hispidus* had the fewest (Kim et al., 2020). *I. glomeratus* comprised 23 scaffolds, and the GC content across all seven genomes ranged between 47.5 and 48.5%.

### 3.3 Characterization of *Inonotus glomeratus* TPS proteins

**Figure 4:**
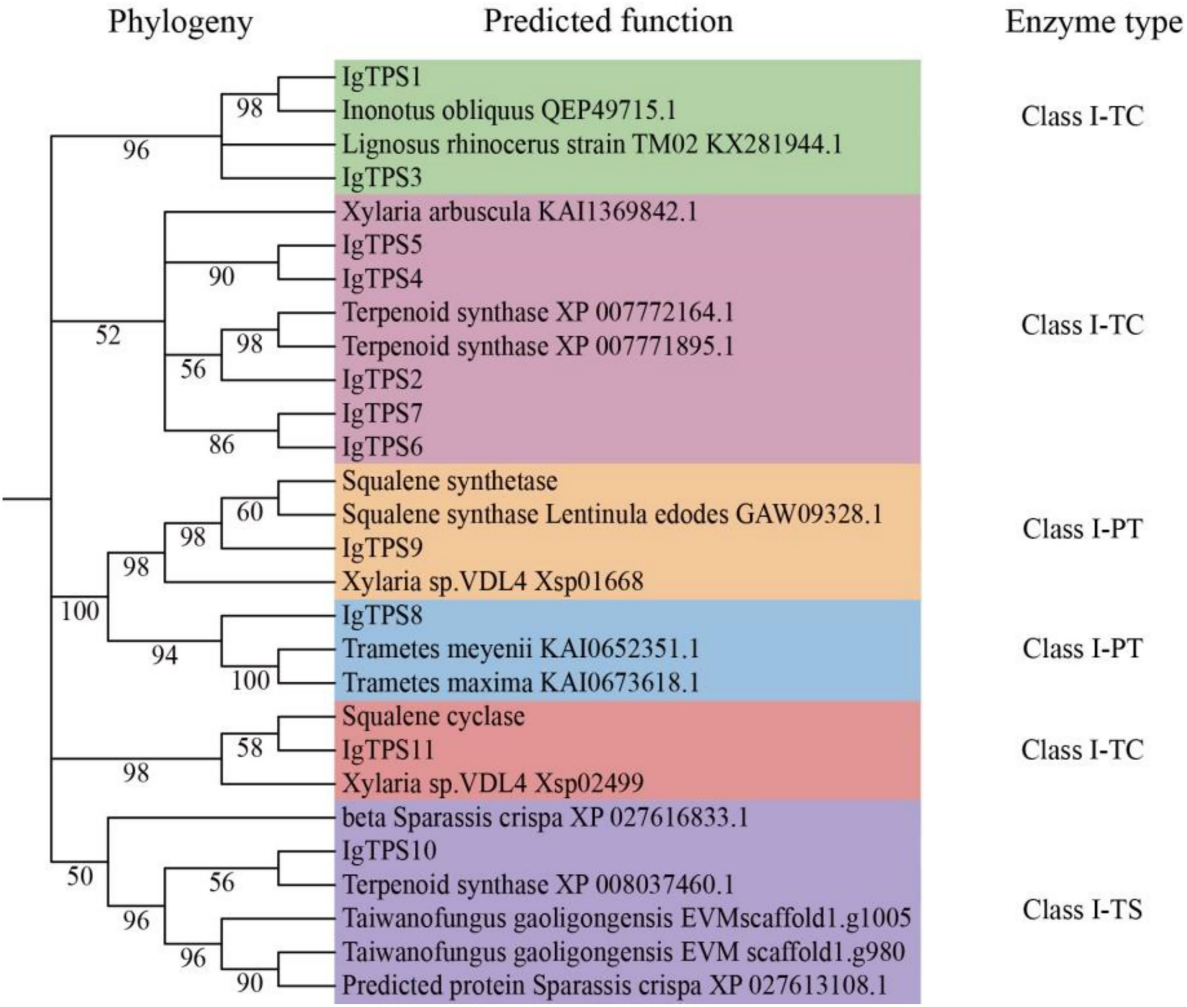
Genomic list of terpenoid biosynthesis in *I. glomeratus*.

A total of 11 TPS genes were identified in the *I. glomeratus* genome, comprising 7 pentalenene synthases (PentS), 1 prenyltransferase (PTase), 1 squalene synthase (SQS), 1 trichodiene synthase (TRI5), and 1 lanosterol synthase (Figure 4). These genes were systematically designated as *IgTPS1* through *IgTPS11*. Phylogenetic analysis revealed that five TPS protein sequences formed a distinct clade with pentalenene synthase sequences from *Xylaria arbuscula* (KAI_1369842) and terpenoid synthases (XP_007772164, XP_007771895). This clustering suggests these enzymes share functional homology with terpenoid synthases and are likely to catalyze isoprene unit polymerization and cyclization to generate diverse terpenoid compounds. Notably, *IgTPS9* contains the conserved “DXXXDD” motif and clusters with squalene synthase sequences from *Xylaria* sp. VDL4 (Xsp_01668) and *Lentinula edodes* (GAW_09328) with 98% sequence identity, indicating potential involvement in triterpenoid biosynthesis. *IgTPS8* shows a close phylogenetic relationship with prenyltransferases from *Trametes meyenii* (KAI_0652351) and *T. maxima* (KAI_0673618). Furthermore, *IgTPS10* clusters with TRI5 sequences from terpenoid synthase (XP_008037460) and *Taiwanofungus gaoligingensis* (EVW scaffold1.1005), suggesting its encoded enzyme participates in type B tetracyclic sesquiterpenoid biosynthesis.

### 3.4 Metabolomics analysis in different media

**Figure 5:**
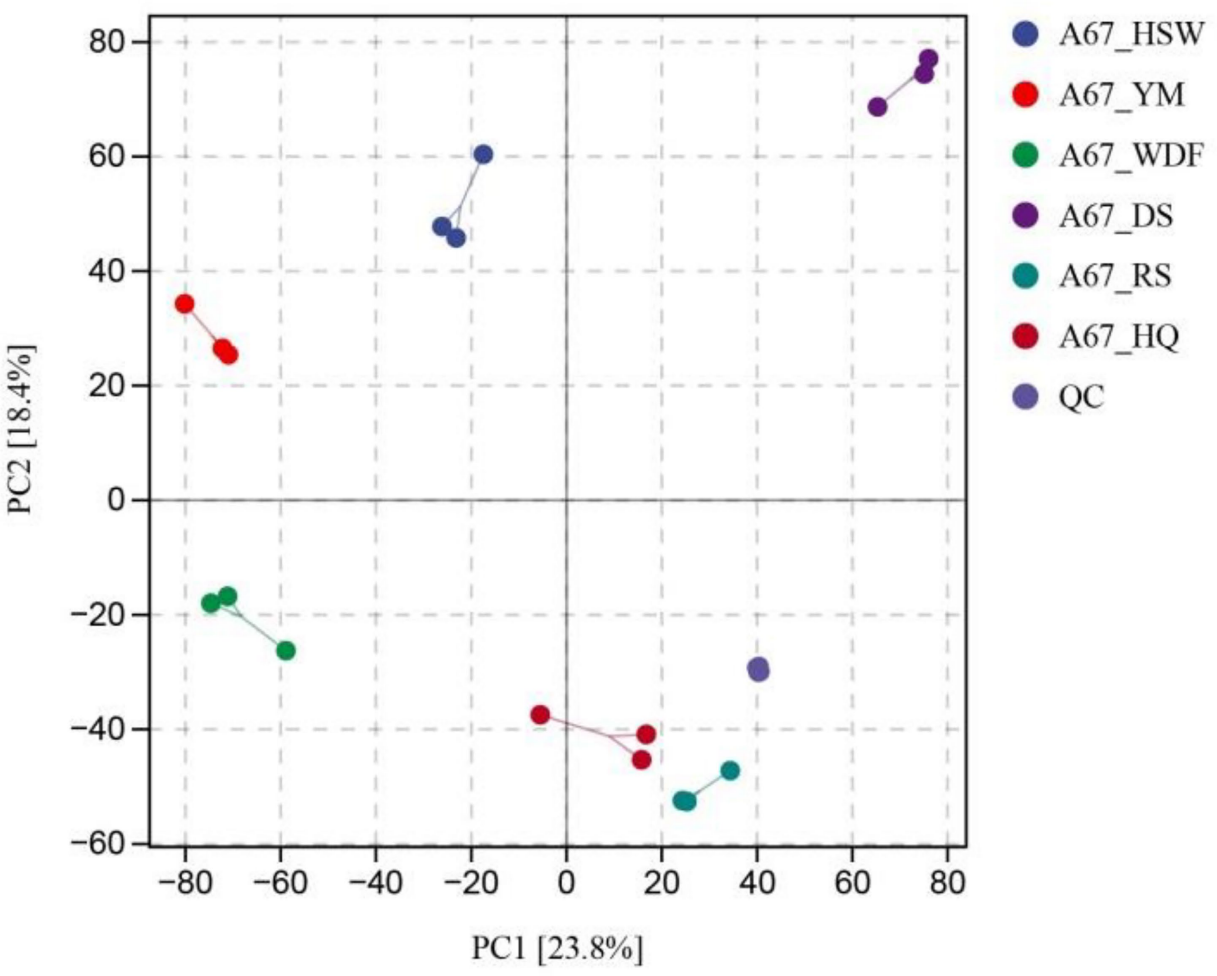
PCA score diagram of mass spectrometry data of each group of samples and quality control samples. PC1 represents the first principal component, PC2 represents the second principal component, and the percentage represents the interpretation rate of the principal component to the data set; each point in the figure represents a sample, the samples of the same group are represented by the same color, and the group is grouped.

The objective of this study was to investigate the diversity of metabolites in *I. glomeratus* under different culture substrate conditions. To this end, *I. glomeratus* was analyzed by untargeted metabolomics in six solid substrate cultures. The identified metabolites encompassed a diverse array of chemical classes, including alkaloids, terpenoids, lipids and lipid-like molecules, organic heterocyclic compounds, and phenylpropanoids and polyketides. In the principal component analysis (PCA) plot, the QC samples were grouped, and parallel samples within the group exhibited analogous compositions, thereby indicating that they possessed analogous metabolic profiles and that the overall analysis was reliable and reproducible (Figure 5). The biological replicate samples from the six different culture substrate conditions were clustered in disparate regions, thereby indicating that the metabolites were significantly different. A total of 1,571 differentially expressed metabolites (DEMs) were identified between HSW and DS, with 1,039 being up-regulated and 532 being down-regulated. Similarly, 1,575 DEMs were identified between HSW and RS, with 1,118 being up-regulated and 457 being down-regulated. Finally, 1,585 DEMs were identified between WDF and RS, with 961 being up-regulated and 634 being down-regulated. A total of 1765 differentially expressed metabolites (DEMs) were identified between YM and DS, with 1,443 being up-regulated and 622 being down-regulated (Supplementary Figure S2). In order to gain a more profound understanding of the metabolic pathways involved in the differentially expressed genes, KEGG significance enrichment analysis was performed on the differentially expressed genes. The top 20 KEGG pathways with the smallest FDR values, i.e., the pathways with the highest degree of enrichment, were selected for the construction of the enrichment factors plot. As shown in Supplementary Figure S3, the following KEGG metabolic pathways were found to be significantly enriched: metabolic pathways, biosynthesis of amino acids, ABC transporters, tryptophan metabolism, aminoacyl-tRNA biosynthesis, D-amino acid metabolism, and others. The analysis revealed that the content of lipids and lipid-like molecules attained its zenith in all culture media and exhibited a significant difference (*p* < 0.05) from all other treatments except RS and DS media. Conversely, the content of alkaloids and their derivatives reached its nadir in HSW, DS, RS, and HQ media, and there was no significant difference (*p* > 0.05) among the matrices (Supplementary Figure S4). Previous studies have reported that secondary metabolites in substrates such as *P. multiflorum*, *C. lacryma-jobi*, *S. miltiorrhiza*, *P. ginseng*, and *A. membranaceus* include terpenoids and alkaloids, suggesting that these additives significantly influenced terpenoid and alkaloid production in this study.

**Figure 6:**
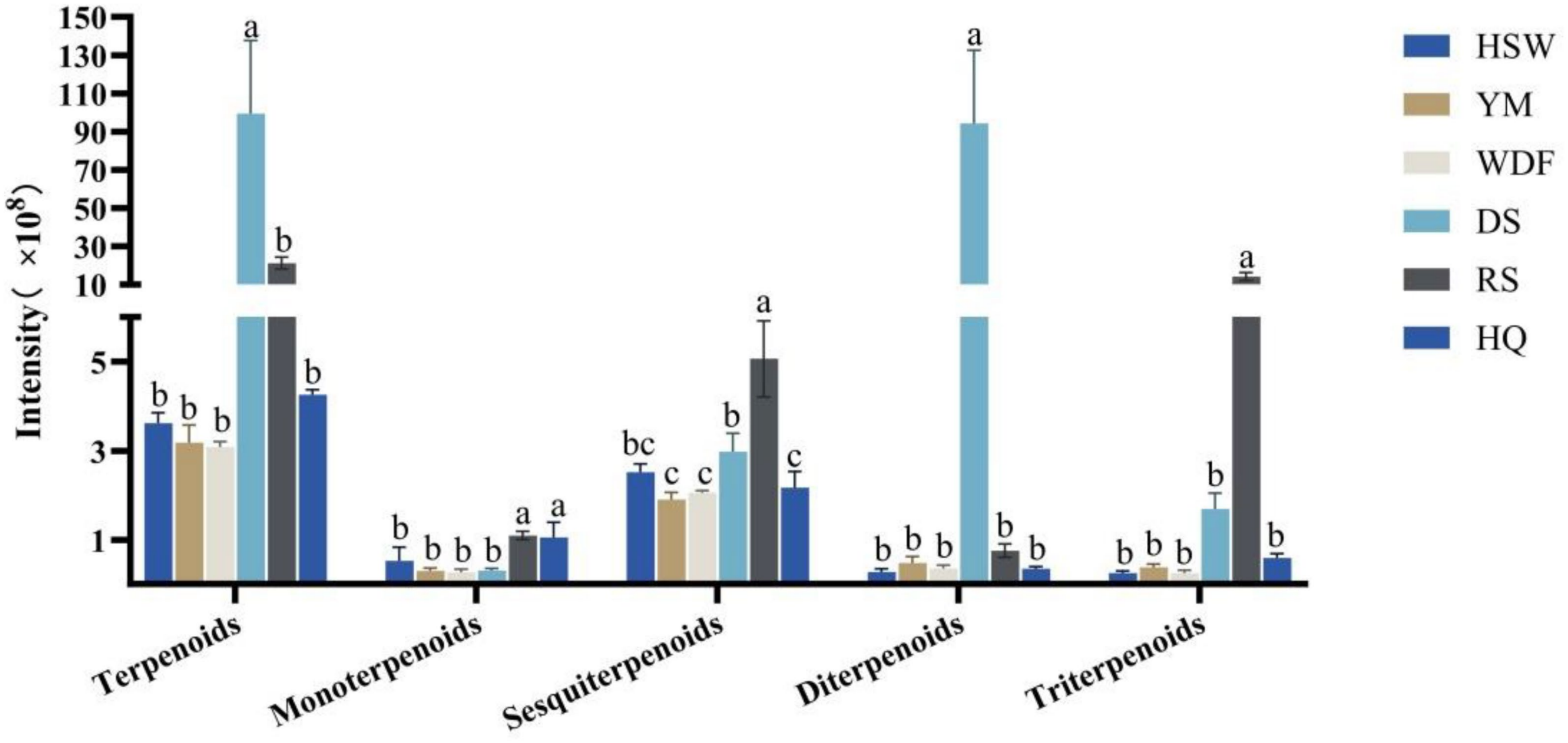
Expression of terpenoid secondary metabolites in each sample of *I. glomeratus* under different culture substrates. Mean ± SD (*n* = 3) was used, and similar letters inside the same treatment are statistically equivalent at *p* < 0.05, based on Tukey’s multiple range test. The horizontal axis delineates the classification of terpene metabolites into distinct categories, namely terpenoids, monoterpenoids, sesquiterpenoids, diterpenoids, and triterpenoids. The vertical axis provides a quantitative representation of the metabolite content.

In DS medium, the terpene content was found to be the highest, with levels ranging from 4 to 32 times higher compared to other culture substrates. The difference in terpene content between the treatments was found to be statistically significant (*p* < 0.05). The compounds identified in the metabolome were categorized into monoterpenes, sesquiterpenes, diterpenes, and triterpenes. The content of diterpenes was the highest in DS medium, with levels ranging from 124 to 331 times higher than those observed in the other culture substrates. In RS medium, the contents of sesquiterpenes and triterpenes were found to be significantly higher than those in the other five culture substrates, with levels ranging from 1 to 2 times and from 8 to 56 times, respectively. Furthermore, the differences among treatments were found to be significant (*p* < 0.05). The content of monoterpenes remained relatively constant across the six culture substrates (Figure 6).

**Figure 7:**
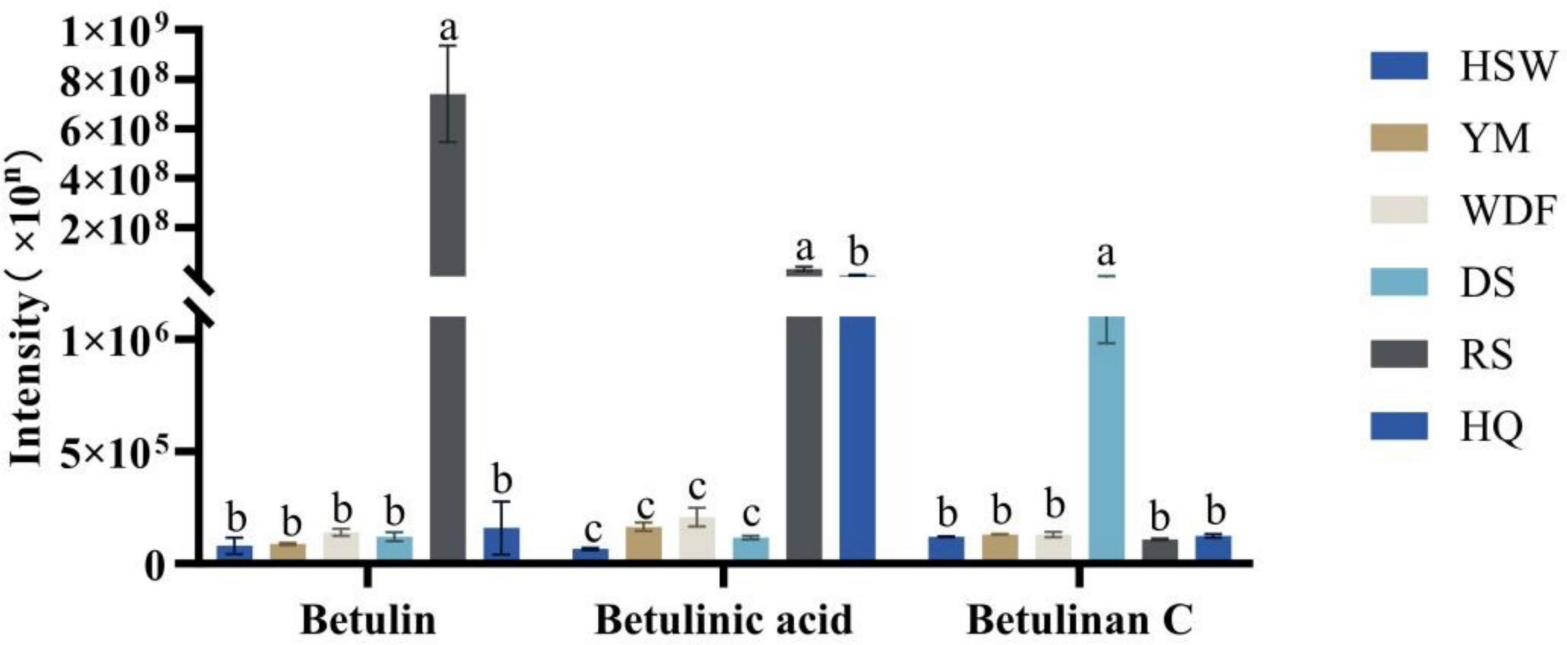
*Inonotus glomeratus* the following investigation is concerned with the content of betulin compounds in each sample under different culture substrates. Mean ± SD (*n* = 3) was used, and similar letters inside the same treatment are statistically equivalent at *p* < 0.05, based on Tukey’s multiple range test. The horizontal axis of the figure indicates the concentration of betulin compounds, while the vertical axis denotes the metabolite content.

A total of betulin, betulinan C, and betulinic acid, which are active triterpenoid compounds, were detected in the metabolome samples. In RS medium, the contents of both betulin and betulinic acid were the highest, ranging from 4,658 to 9,275 times and 4 to 503 times the other culture substrates, respectively. In DS medium, the contents of betulinan C were the highest, ranging from 19 times of HSW, 17 times of YM, 17 times of WDF, 21 times of RS, and 18 times of HQ, and the differences among treatments were significant (*p* < 0.05). In other cultures, the contents of betulin and betulinic acid were the highest. The differences among treatments were found to be statistically significant (*p* < 0.05). In contrast, in other culture media, the contents of the three compounds were lower and did not differ significantly (Figure 7).

**Figure 8:**
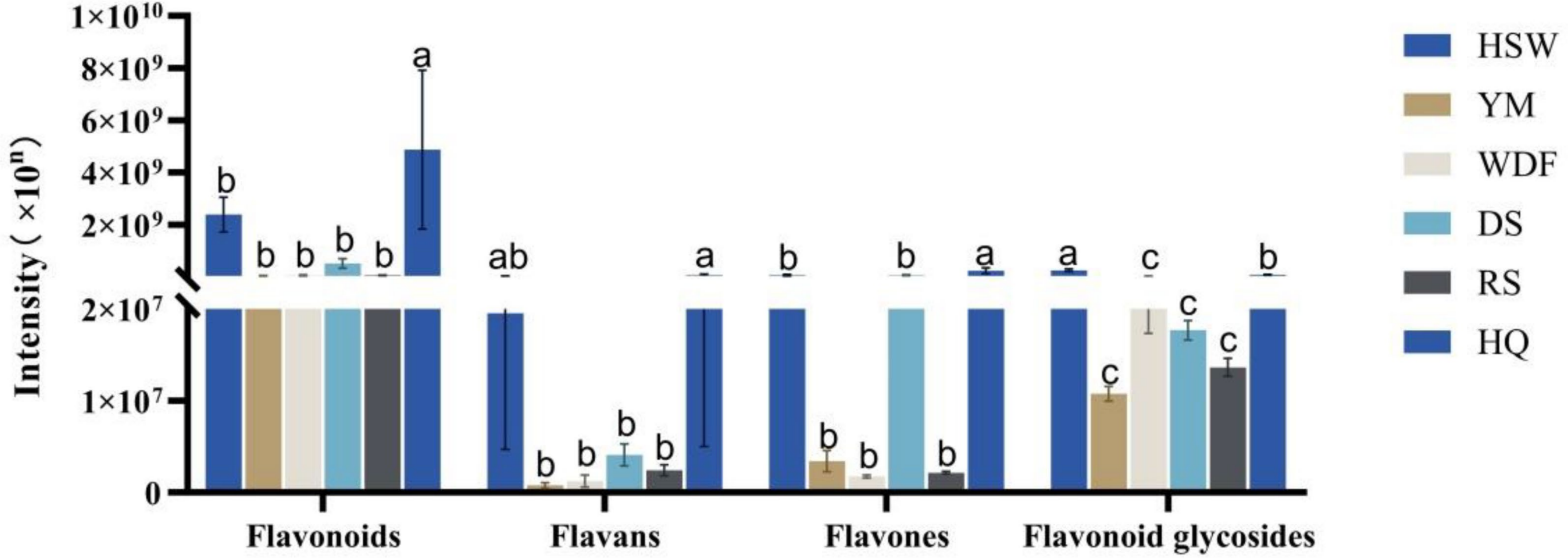
Expression of flavonoid secondary metabolites in each sample of *I. glomeratus* under different culture substrates. Mean ± SD (*n* = 3) was used, and similar letters inside the same treatment are statistically equivalent at *p* < 0.05, based on Tukey’s multiple range test. The horizontal axis indicates that the flavonoid metabolites are flavonoids, flavans, flavones, and flavonoid glycosides, and the vertical axis indicates the metabolite content.

In HQ medium, flavonoid content demonstrated the greatest levels, exhibiting a range of 2 to 138 times the levels observed in other culture substrates. The discrepancy between these treatments was found to be statistically significant (*p* < 0.05). The study demonstrated that the secondary metabolites of *A. membranaceus* were predominantly flavonoids, thereby signifying that the alterations in flavonoid content observed in this study were considerably influenced by the presence of *A. membranaceus*. The contents of flavonoids and flavonoid glycosides were found to be the highest in HQ medium, with levels 2–71 and 3–133 times higher, respectively, compared to other culture substrates. The flavonoid glycosides were found to be most abundant in the HSW medium, exhibiting a concentration that was 22 times higher than that of the YM medium, 10 times higher than that of the WDF medium, 13 times higher than that of the DS medium, 18 times higher than that of the RS medium, and 3 times higher than that of the HQ medium, with statistically significant differences across all treatments (*p* < 0.05) (Figure 8).

### 3.5 Transcriptome analysis in different media

**Figure 9:**
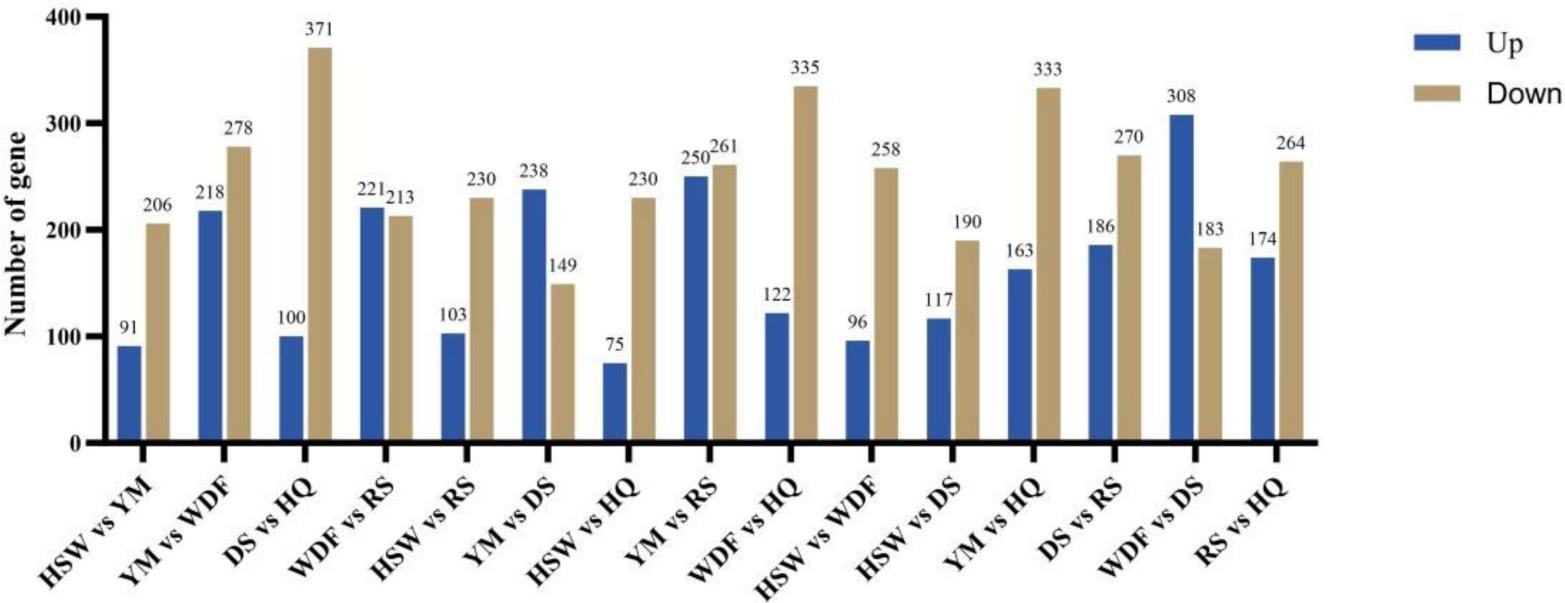
Analysis of differentially expressed genes (DEGs) of *I. glomeratus* under different culture substrates. The *x*-axis represents different culture substrates, and the *y*-axis represents the number of differentially expressed genes.

Through bioinformatic analysis of the sequencing results, low-quality reads—including adapter sequences and unidentified or low-quality bases—were removed. Table 4 presents the statistics of clean mapped reads obtained from RNA-seq analysis. The proportions of clean reads for HSW, YM, WDF, DS, RS, and HQ were 98.75, 98.6, 98.91, 98.94, 98.78, and 98.79%, respectively. The total read alignment rates for the sequenced samples were 96.16, 95.90, 96.53, 96.35, 96.37, and 96.60%, respectively (Table 4). Differentially expressed genes (DEGs) were analyzed across different sample comparisons (Figure 9). Specifically, HSW vs. YM exhibited 297 DEGs (91 up-regulated, 206 down-regulated), while YM vs. WDF showed 496 DEGs (218 up-regulated, 278 down-regulated). Similarly, WDF vs. RS had 434 DEGs (221 up-regulated, 213 down-regulated), and HSW vs. RS displayed 333 DEGs (103 up-regulated, 230 down-regulated). Furthermore, YM vs. DS contained 387 DEGs (238 up-regulated, 149 down-regulated), whereas YM vs. RS revealed 511 DEGs (250 up-regulated, 261 down-regulated). Lastly, WDF vs. DS demonstrated 491 DEGs (308 up-regulated, 183 down-regulated). KEGG pathway enrichment analysis revealed that the differentially expressed genes (DEGs) were predominantly enriched in pathways related to amino sugar and nucleotide sugar metabolism, glycosphingolipid biosynthesis—globo and isoglobo series, longevity regulation pathway—multiple species, pentose and glucuronate interconversions, nitrogen metabolism, and starch and sucrose metabolism. To further elucidate the functional roles of these DEGs in associated biological processes, Gene Ontology (GO) enrichment analysis was performed. The results demonstrated significant enrichment in molecular functions (MF) such as oxidoreductase activity, misfolded protein binding, and hydrolase activity (acting on glycosyl bonds). Additionally, DEGs were enriched in cellular components (CC), including chromosomes and the GET complex, as well as biological processes (BP) such as terpenoid indole alkaloid biosynthesis, small molecule catabolism, and stimulus response.

**Table 4:** Summary of sequencing data quality and statistical information for transcriptome assembly.

Sample	Clean reads	Q20 (%)	Q30 (%)	GC content (%)	Total mapped	Multiple mapped	Uniquely mapped
HSW	48,808,008	99.05	97.09	51.88	46,931,657 (96.16%)	1,086,809 (2.32%)	45,844,848 (97.68%)
YM	47,462,476	98.98	96.92	51.51	45,515,751 (95.90%)	1,447,828 (3.18%)	44,067,923 (96.82%)
WDF	50,337,982	99.18	97.45	51.42	48,593,150 (96.53%)	1,514,343 (3.12%)	47,078,807 (96.88%)
DS	47,226,964	99.14	97.31	51.57	45,504,781 (96.35%)	1,244,082 (2.73%)	44,260,699 (97.27%)
RS	60,831,394	99.06	97.12	51.67	58,625,773 (96.37%)	1,460,664 (2.49%)	57,165,109 (97.51%)
HQ	48,159,336	99.06	97.12	51.65	46,520,514 (96.60%)	1,435,364 (3.09%)	45,085,150 (96.91%)

### 3.6 Integrated metabolomics and transcriptomics analysis

**Figure 10:**
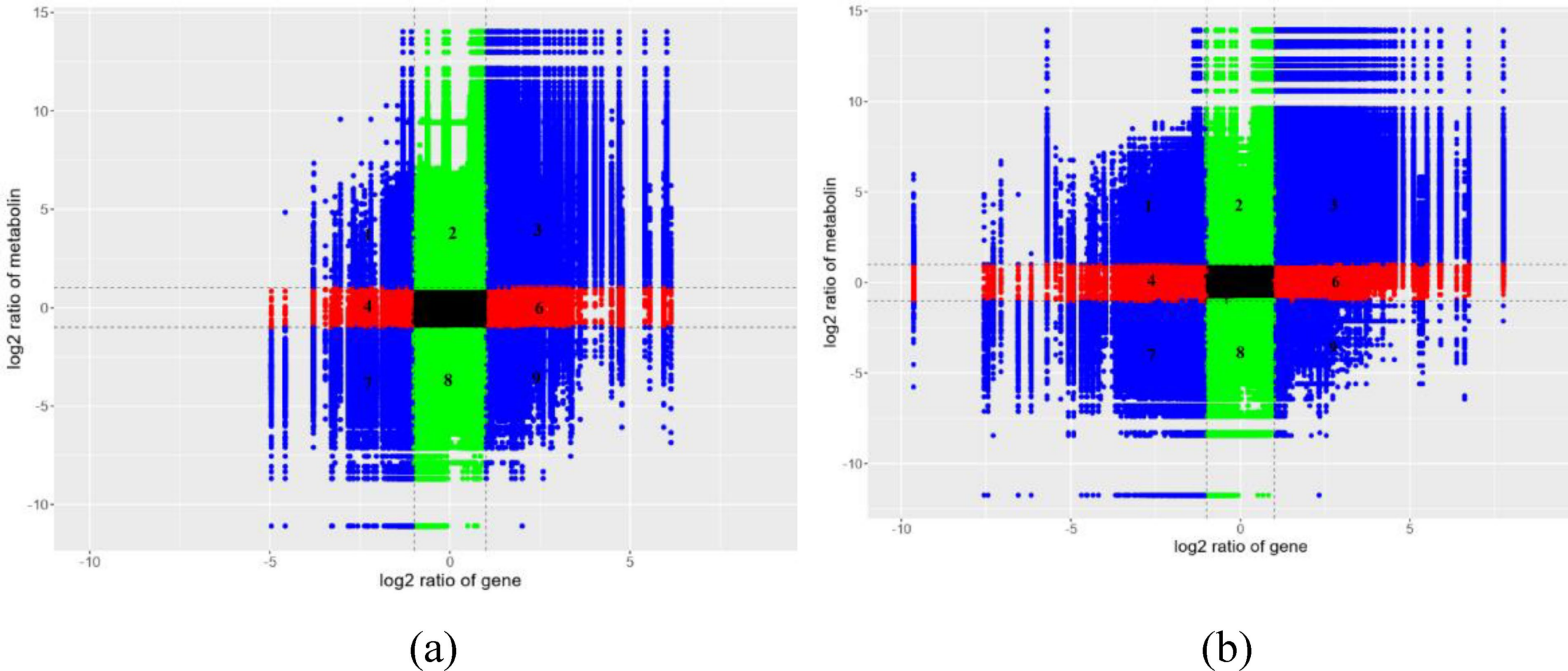
Integrated metabolomics and transcriptomics analysis of *I. glomeratus*. **(a)** YM vs. DS; **(b)** YM vs. RS. The horizontal axis represents the fold change of genes, and the vertical axis represents the fold change of metabolites. The dotted line marks the position of |Log2FC >1|. From left to right and top to bottom, they are divided into 1–9 quadrants in sequence. Black: Neither genes nor metabolites are differentially expressed. The genes and metabolites in this differential group are not differentially expressed. Left diagonal: Genes and metabolites have the same differential expression pattern, and for the genes and metabolites with consistent regulatory trends, the change of metabolites may be positively regulated by genes. Right diagonal: Genes and metabolites have the same differential expression pattern, and for the genes and metabolites with inconsistent regulatory trends, the change of metabolites may be negatively regulated by genes. Red, green: Metabolites remain unchanged, while genes are up- or down-regulated, or genes remain unchanged while metabolites are up- or down-regulated.

Integrative analysis of transcriptional and metabolic networks was performed by stringent multi-omics correlation profiling (Pearson correlation coefficient thresholded at |*r*| > 0.95). In this Cartesian framework, co-regulated molecular pairs clustered in diagonal sectors (positive correlation: quadrants 3 and 7; inverse relationships: quadrants 1 and 9), while baseline expression units populated the central zone (Figure 10; Supplementary Figure S5). This spatial organization revealed a tight interconnectivity between transcriptional reprogramming and metabolic restructuring, suggesting candidate regulatory nodes for phenotype modulation. Through KEGG pathway enrichment analysis, an integrated study was conducted on the DEGs and DEMs in *I. glomeratus*, aiming to systematically reveal the regulatory association between gene expression and metabolite level changes (Supplementary Figure S6). Furthermore, through correlation analysis, the degree of association between gene expression levels in the transcriptome and metabolite contents in the metabolome was quantified, thereby analyzing the potential impact of gene expression changes on metabolite accumulation, as well as the possible feedback regulatory mechanism of metabolite dynamics on gene expression. The results of this correlation analysis were presented in the form of a heatmap, visually reflecting the correlation trend between genes and metabolites and their clustering characteristics (Supplementary Figure S7). Additionally, by constructing a correlation network diagram, metabolic-gene regulatory pairs with strong correlations between differentially expressed genes and metabolites were identified and displayed (Supplementary Figure S8). To further analyze the complex relationship between genes and metabolites, chord diagrams were used to visualize the key correlation patterns, clearly presenting the interaction network of multiple pairs of genes and metabolites (Supplementary Figure S9).

### 3.7 Gene expression analysis of IgPKS and IgTPS in different media

**Figure 11:**
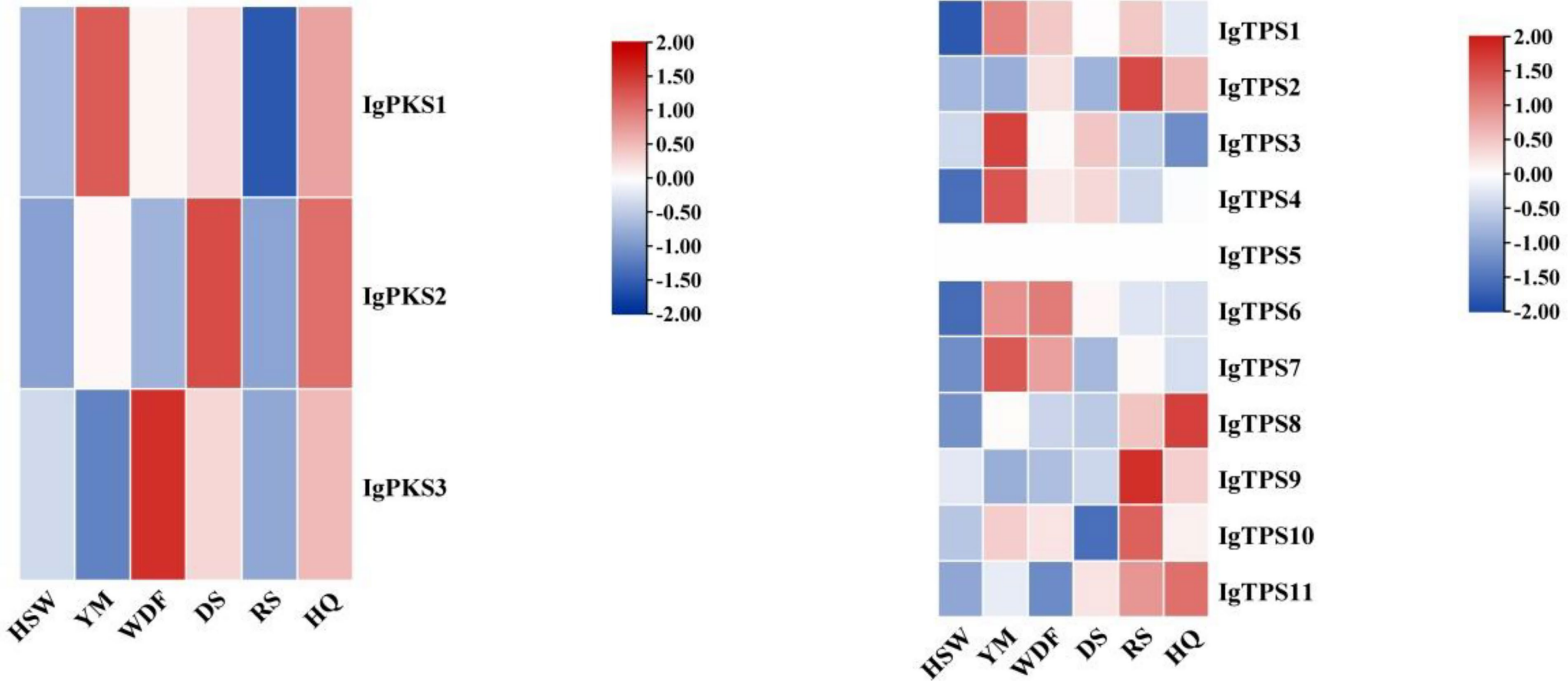
Interactive heatmap of *IgPKS* and *IgTPS* gene expressions under different substrate culture conditions (left: *IgPKS*; right: *IgTPS*). The horizontal axis represents different culture substrates, and the vertical axis represents gene expression. The expression levels are encoded by colors, with red and blue representing high and low expression, respectively.

Gene expression analysis of *IgPKS* and *IgTPS* in different media conditions revealed distinct expression patterns. In YM medium, *IgPKS1*, *IgTPS1*, *IgTPS3*, *IgTPS4*, and *IgTPS7* exhibited significantly higher expression levels compared to other treatments. Similarly, *IgPKS2* showed elevated expression in DS medium, while *IgPKS3* was upregulated in WDF medium. In RS medium, *IgTPS2*, *IgTPS10* and *IgTPS9* were significantly more highly expressed than in other treatments. Additionally, *IgTPS8* demonstrated markedly higher expression in HQ medium (Figure 11). These findings suggest that PKS and TPS gene expression vary significantly depending on the substrate, highlighting substrate-specific regulatory mechanisms.

### 3.8 Gene expression analysis of IgTFs in different media

**Figure 12:**
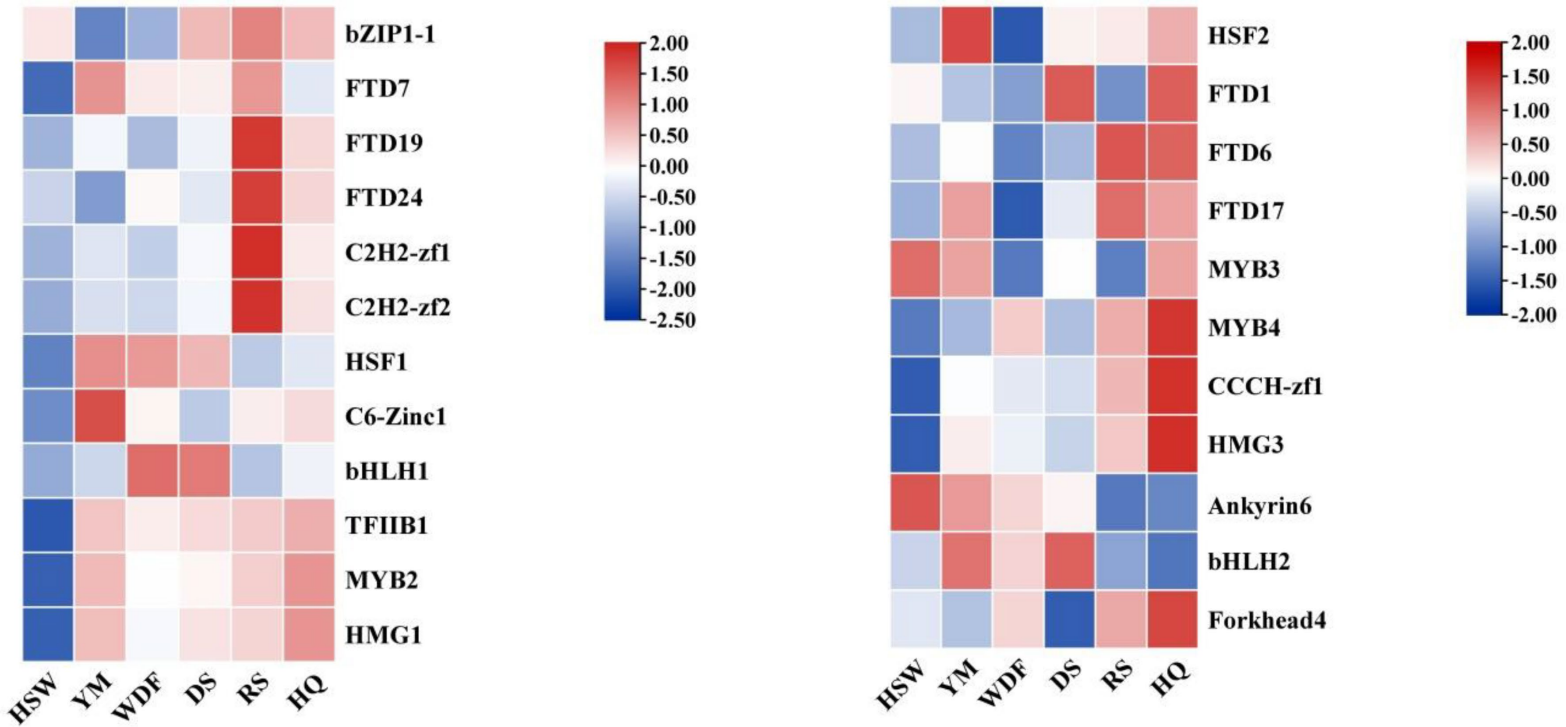
Interactive heatmap of *IgTFs* gene expression under different substrate culture conditions. The horizontal axis represents different culture substrates, and the vertical axis represents gene expression. The expression levels are encoded by colors, with red and blue representing high and low expression, respectively.

The analysis of gene expression of *IgTFs* under different media conditions revealed significant differences in the expression of transcription factors (TFs) across various media. Specifically, under YM medium, the expression level of *IgC6-Zinc1* was significantly higher than that of other treatments. Under WDF and DS medium, the expression level of *IgbHLH1* was significantly higher than that of other treatments. Under RS medium, the expression levels of *IgbZIP1-1*, *IgFTD7*, *IgFTD24*, *IgC2H2-zf1*, and *IgC2H2-zf2* were upregulated. Under HQ medium, the expression levels of *IgHSF2*, *IgFTD1*, *IgFTD6*, *IgFTD17*, *IgMYB3*, *IgMYB4*, *IgCCCH-zf1*, *IgHMG3*, and *IgForkhead4* were significantly higher than those of other treatments (Figure 12).

### 3.9 Analysis of secondary metabolite biosynthetic genes

**Figure 13:**
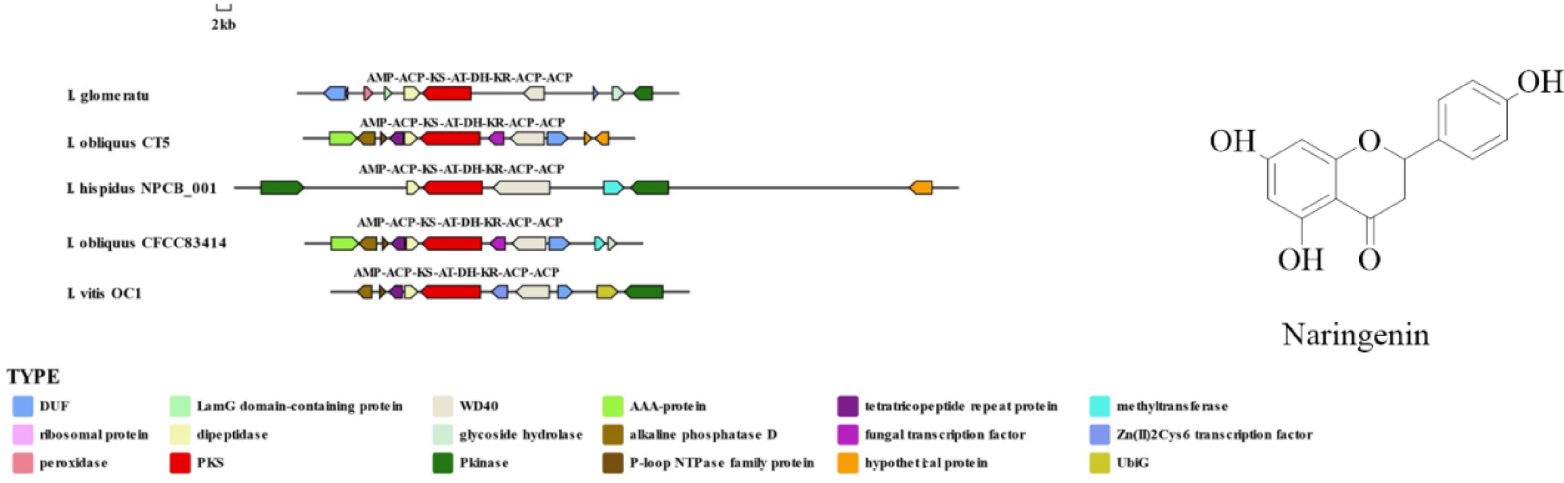
Comparison of biosynthesis of the hypothetical naringenin biosynthetic gene cluster.

**Figure 14:**
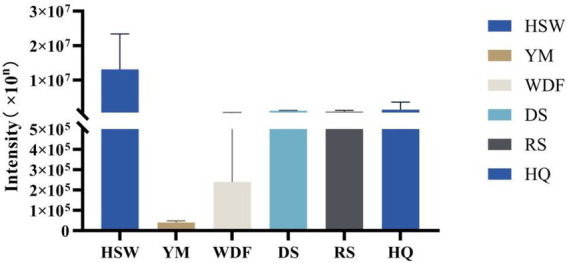
Relative abundance of naringenin under different media conditions. The *x*-axis represents different media conditions, and the *y*-axis represents the content of naringenin.

**Figure 15:**
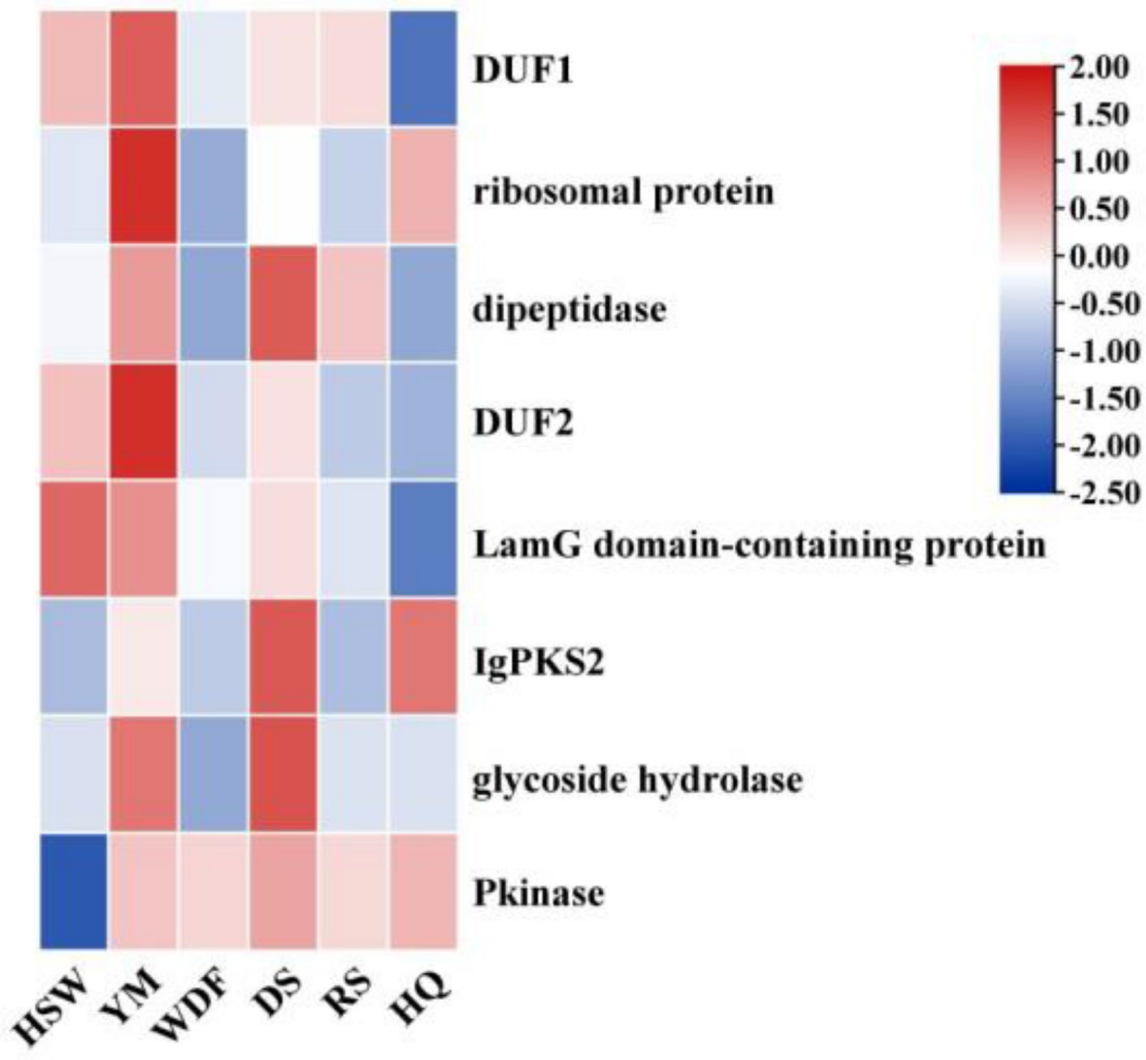
Heatmap of the expression levels of *IgPKS2* and its surrounding genes under different media substrates. The horizontal axis represents different media substrates. The vertical axis represents gene expression, and the expression levels are encoded by colors, with red and blue indicating high and low expression, respectively.

This study identified 16 biosynthetic gene clusters (BGCs) in the genome of *I. glomeratus* through antiSMASH and local BLAST analysis. The PKS genes in *I. glomeratus* exhibited the following domain architectures: *IgPKS1* (SAT-KS-AT-PT-ACP-ACP-TE), *IgPKS2* (AMP-ACP-KS-AT-DH-KR-ACP-ACP), and *IgPKS3* (KS-ACP-TE-AT). Comparative analysis of the *IgPKS2* protein kinase domains across different species—*I. obliquus* (CT5), *I. hispidus* (NPCB001), *I. vitis* (OC1), and *I. obliquus* (CFCC83414)—revealed that all four genomes encoded proteins structurally similar to *IgPKS2*, containing the conserved PKS domain arrangement AMP-ACP-KS-AT-DH-KR-ACP-ACP (Figure 13). Phylogenetic analysis further demonstrated that *IgPKS2* clustered closely with these four PKS genes, exhibiting high homology (Supplementary Figure S10). Furthermore, gene deletions and horizontal gene transfer events were observed within each gene cluster. Variations in flanking modifying genes underscored the diversity of flavonoid biosynthesis while highlighting the coexistence of high conservation and pathway plasticity. Given that *IgPKS2* homologs in the comparative genomes primarily contribute to flavonoid biosynthesis, we hypothesize that *IgPKS2* functions predominantly in the synthesis of naringenin or its derivatives. Notably, the domain architecture of *IgPKS2*—particularly the core domains (AMP, ACP, KS, AT, DH, KR, and ACP)—was highly conserved across species, though minor structural variations likely reflect functional adaptations to specific ecological niches. Metabolomic analysis indicated that naringenin levels were significantly elevated in HSW medium compared to other conditions (Figure 14). This finding aligned closely with the expression patterns observed in heatmap data (Figure 15), reinforcing the robustness of the results. The strong correlation between gene expression dynamics and naringenin accumulation suggests that transcriptional regulation directly modulates naringenin biosynthesis, ultimately governing its differential production under varying culture substrates.

**Figure 16:**
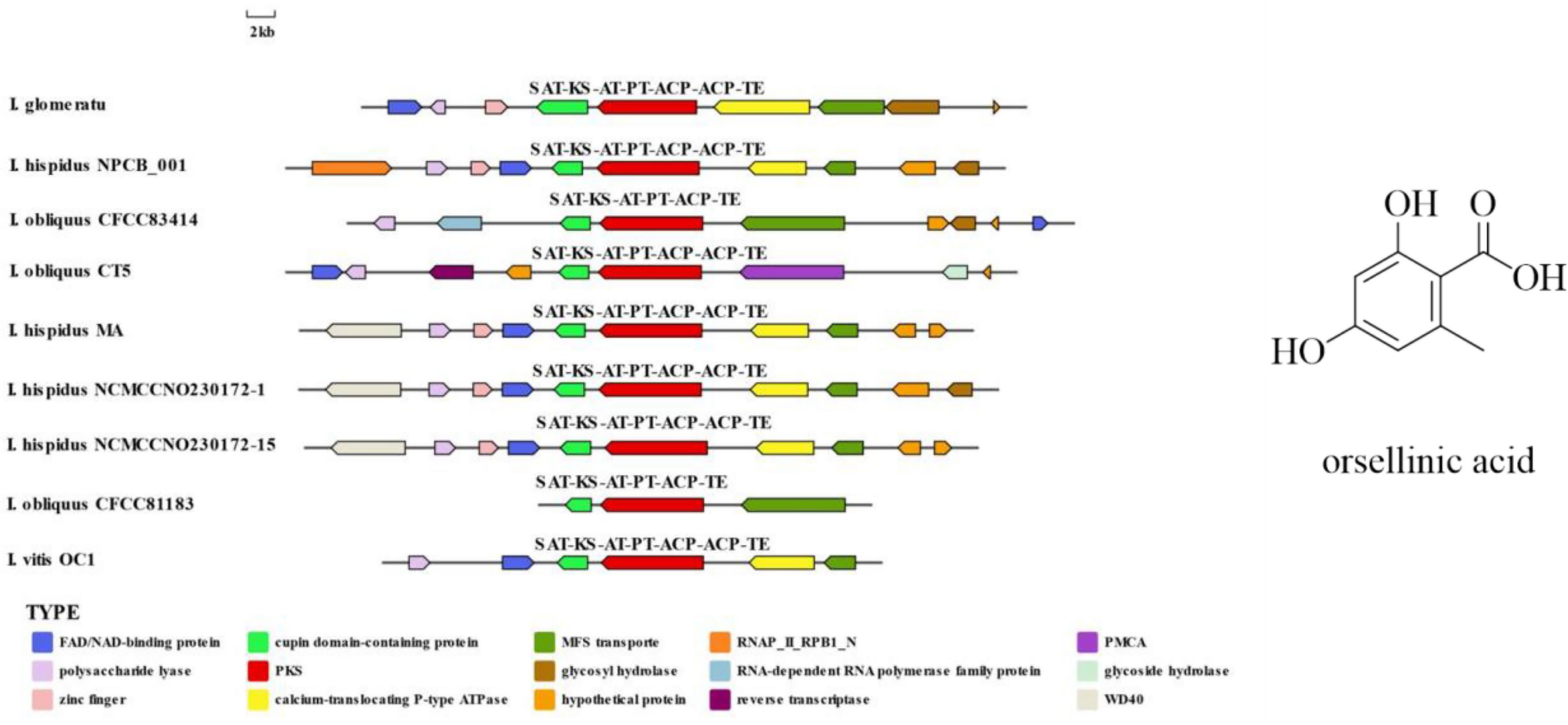
Comparison of biosynthesis of the hypothetical orsellinic acid biosynthetic gene cluster.

**Figure 17:**
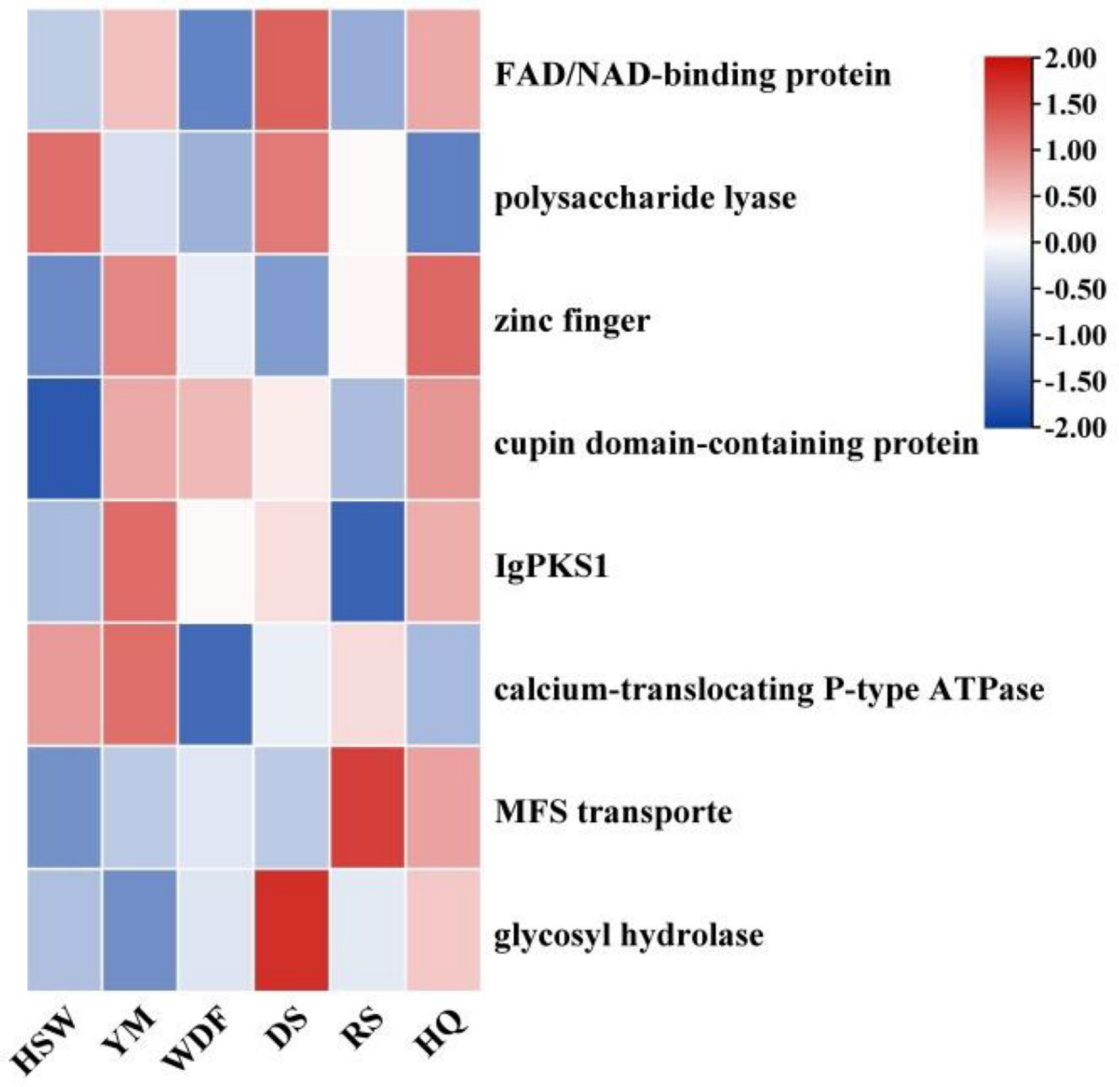
Transcriptional expression of *IgPKS1* and its surrounding genes under different culture substrate conditions. The horizontal axis indicates different culture substrates. The *y*-axis represents gene expression. The expression levels are color-coded, with red and blue indicating high and low expression, respectively.

*IgPKS1* possesses a domain structure that is similar to that of other known PKS genes, comprising SAT-KS-AT-PT-ACP-ACP-TE domains. By clustering *IgPKS1* with eight known PKS genes and constructing a phylogenetic tree (Supplementary Figure S10), we found that *IgPKS1* clustered closely with these genes, exhibiting a high degree of homology. Based on the analysis of the phylogenetic tree and comparison of gene clusters, it is hypothesized that *IgPKS1* may be involved in the biosynthesis of orsellinic acid or its derivatives. Furthermore, the following species of *I. hispidus* (NPCB001), *I. obliquus* (CT5), *I. hispidus* (MA), *I. hispidus* (NCMCCNO230172-1), *I. hispidus* (NCMCCNO230172-15), *I. vitis* (OC1), and *I. obliquus* (CFCC83414) have been found to possess the same modifier genes as *I. glomeratus.* All containing cupin domain-containing proteins, FAD/NAD-binding proteins, polysaccharide lyases, MFS transporters, and calcium-translocating P-type ATPases (Figure 16). Therefore, it is speculated that *IgPKS1* can catalyze the synthesis of orsellinic acid or its derivatives. Sequence alignment analysis revealed that the SAT-KS-AT-PT-ACP-ACP-TE domain is highly conserved across different species, which may be related to its key role in the kinase function of the *IgPKS1* protein. Conversely, the variability of this domain among different species may be associated with their specific biological functions or adaptability. The study also found that in YM medium, the gene expression levels were significantly higher than those under other conditions (Figure 17). This suggests that the upregulation or downregulation of gene expression may play a crucial role in the biosynthesis of orsellinic acid or its derivatives.

**Figure 18:**
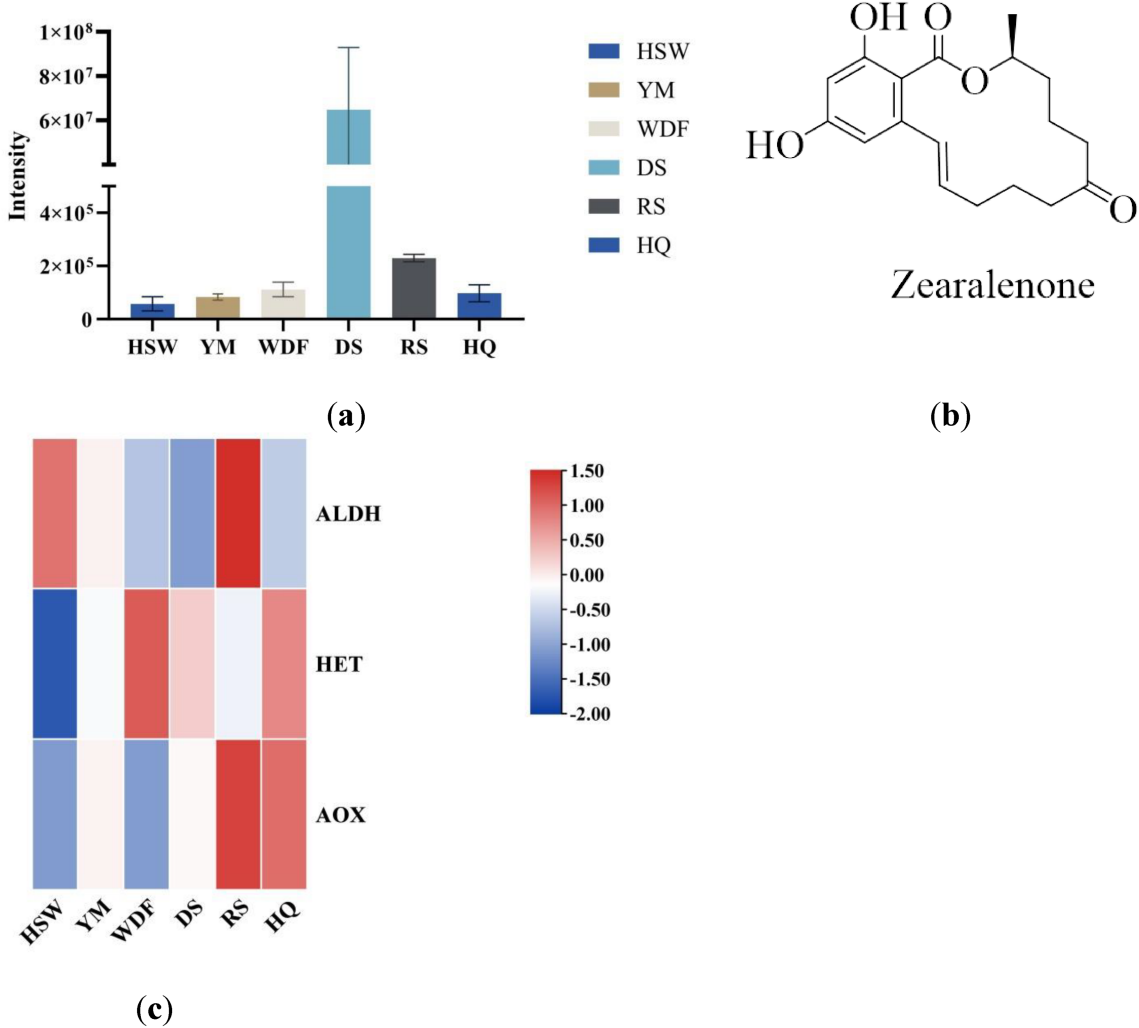
This figure shows the expression levels of secondary metabolites **(a)** and the chemical structures **(b)** of zearalenone in different culture media for each sample. The *x*-axis represents different culture substrates, and the *y*-axis represents the content of zearalenone. **(c)** An interactive heatmap of gene expression. The *x*-axis represents different culture substrates. The *y*-axis represents gene expression. The expression levels are encoded by colors, with red and blue representing high and low expression, respectively. ALDH, aldehyde dehydrogenase; HET, heterocyclic transferase; AOX, alcohol oxidase.

In this study, zearalenone—a derivative of orsellinic acid—was identified in both the metabolome and genome. Analysis of the genes associated with its biosynthetic pathway revealed that several key enzymes, including aldehyde dehydrogenase (ALDH), heterocyclic transferase (HET), and alcohol oxidase (AOX), are involved and play crucial roles in zearalenone biosynthesis. Further investigation demonstrated that in RS medium, the gene expression levels of ALDH and AOX were significantly elevated compared to other conditions (Figure 18), suggesting that the upregulation or downregulation of these genes may directly influence zearalenone biosynthesis. Additionally, quantification of zearalenone under different cultivation conditions revealed that its content was substantially higher in DS media than in other conditions (Figure 18), indicating that substrate composition significantly affects zearalenone production.

**Figure 19:**
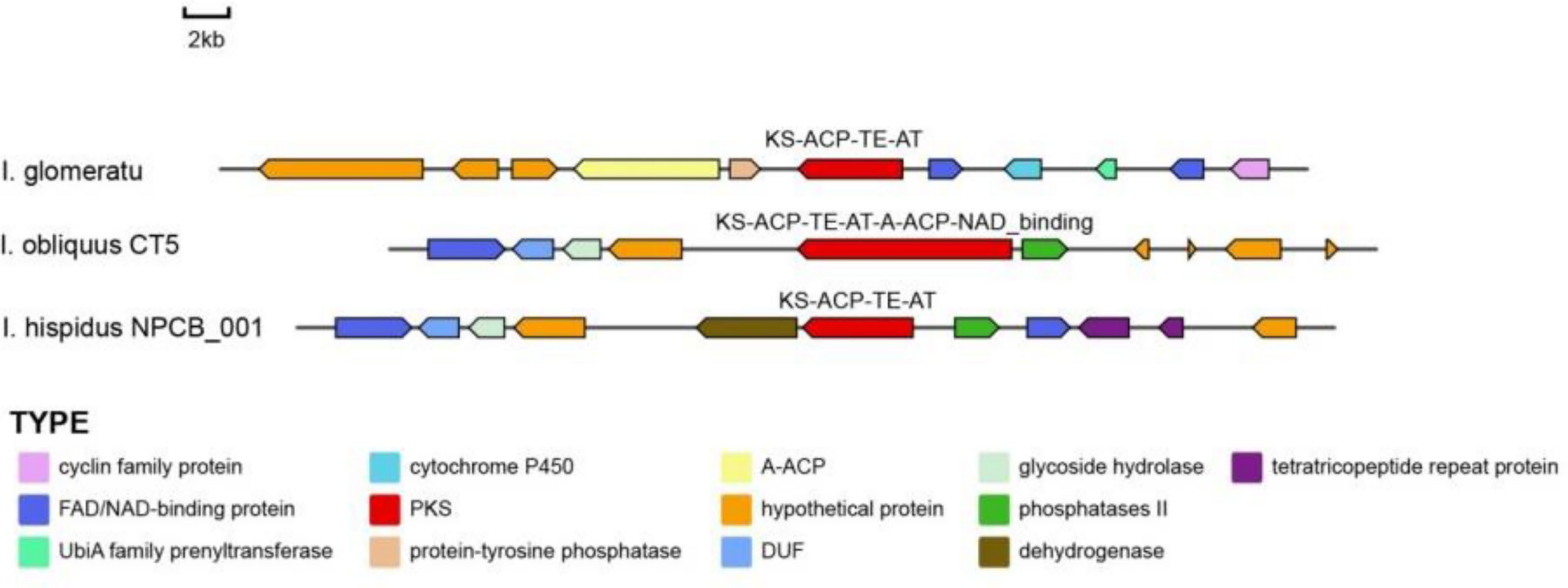
A comparative analysis of the genes surrounding *IgPKS3* and related species was performed.

In this study, *IgPKS3* comprises a domain architecture (KS-ACP-TE-AT) similar to that of *I. hispidus* (NPCB001) and *I. obliquus* (CT5) (Figure 19). Phylogenetic analysis revealed that *IgPKS3* clusters with these two PKS genes and exhibits high homology (Supplementary Figure S10). Sequence alignment analysis demonstrated that the KS-ACP-TE-AT domain is highly conserved across different species, which may be related to its critical role in the function of the *IgPKS3* protein. Concurrently, the variability of this domain among different species may be associated with their specific biological functions or adaptability.

### 3.10 Biosynthesis of betulinic acid in *Inonotus glomeratus*

**Figure 20:**
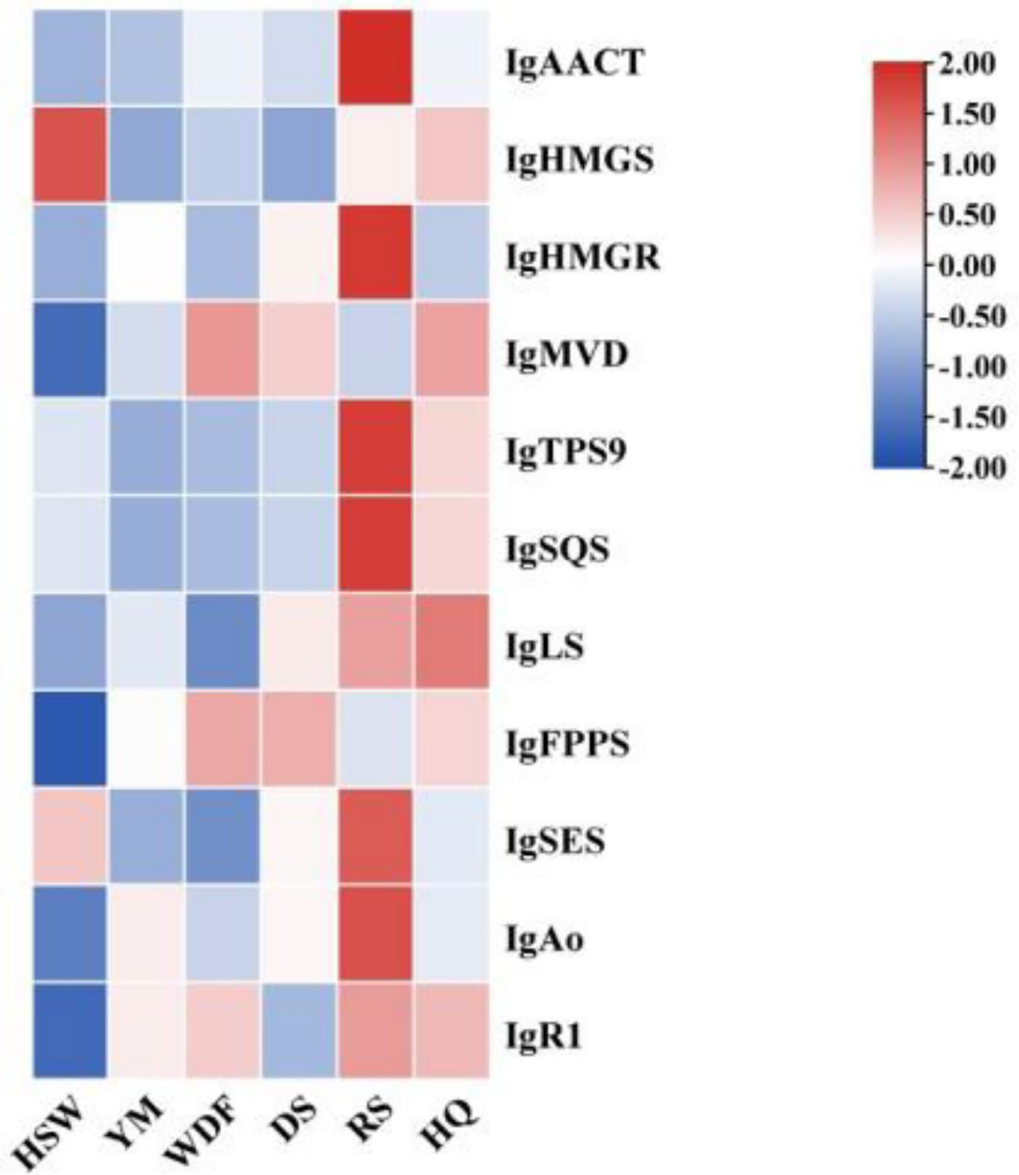
Heatmap showing the expression levels of enzyme genes in the betulinic acid biosynthesis of the mevalonic acid (MVA) pathway. The *x*-axis represents different culture substrates, and the *y*-axis represents gene expression. The expression levels are encoded by colors, with red and blue representing high and low expression, respectively.

**Figure 21:**
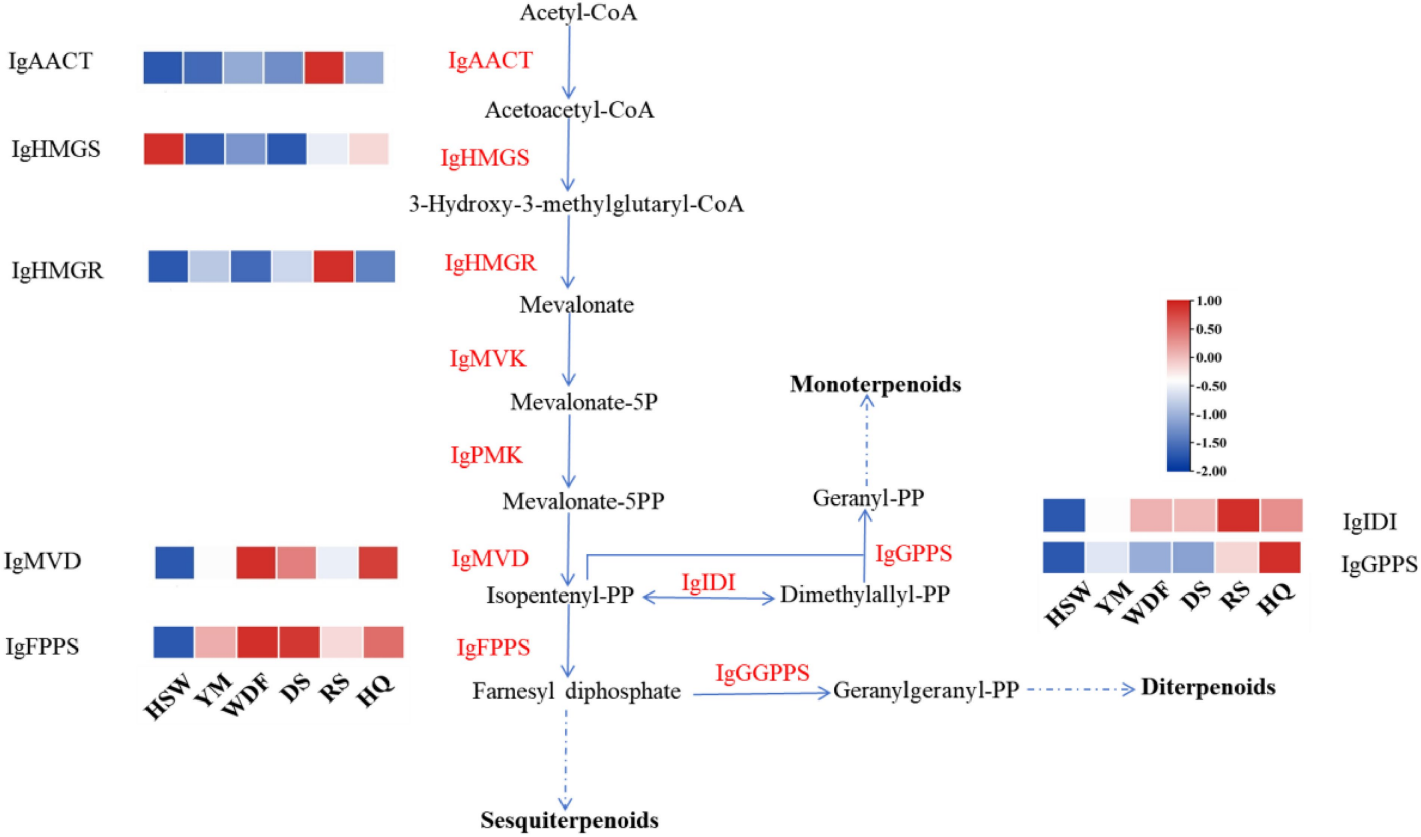
This figure shows the enzymatic reactions in the mevalonate (MVA) pathway in *I. glomeratus* and the expression levels of some key genes. The *x*-axis represents different culture substrates, and the *y*-axis represents gene expression. The expression levels are color-coded, with red and blue indicating high and low expression, respectively. Dashed lines represent steps made up of several enzyme reactions. AACT, acetoacetyl-CoA thiolase; HMGS, HMG-CoA synthase; HMGR, HMG-CoA reductase; PMK, phosphomevalonate kinase; MVD, mevalonate-5-pyrophosphate decarboxylase; IDI, isopentenyl diphosphate isomerase; GPPS, geranyl diphosphate synthase.

**Figure 22:**
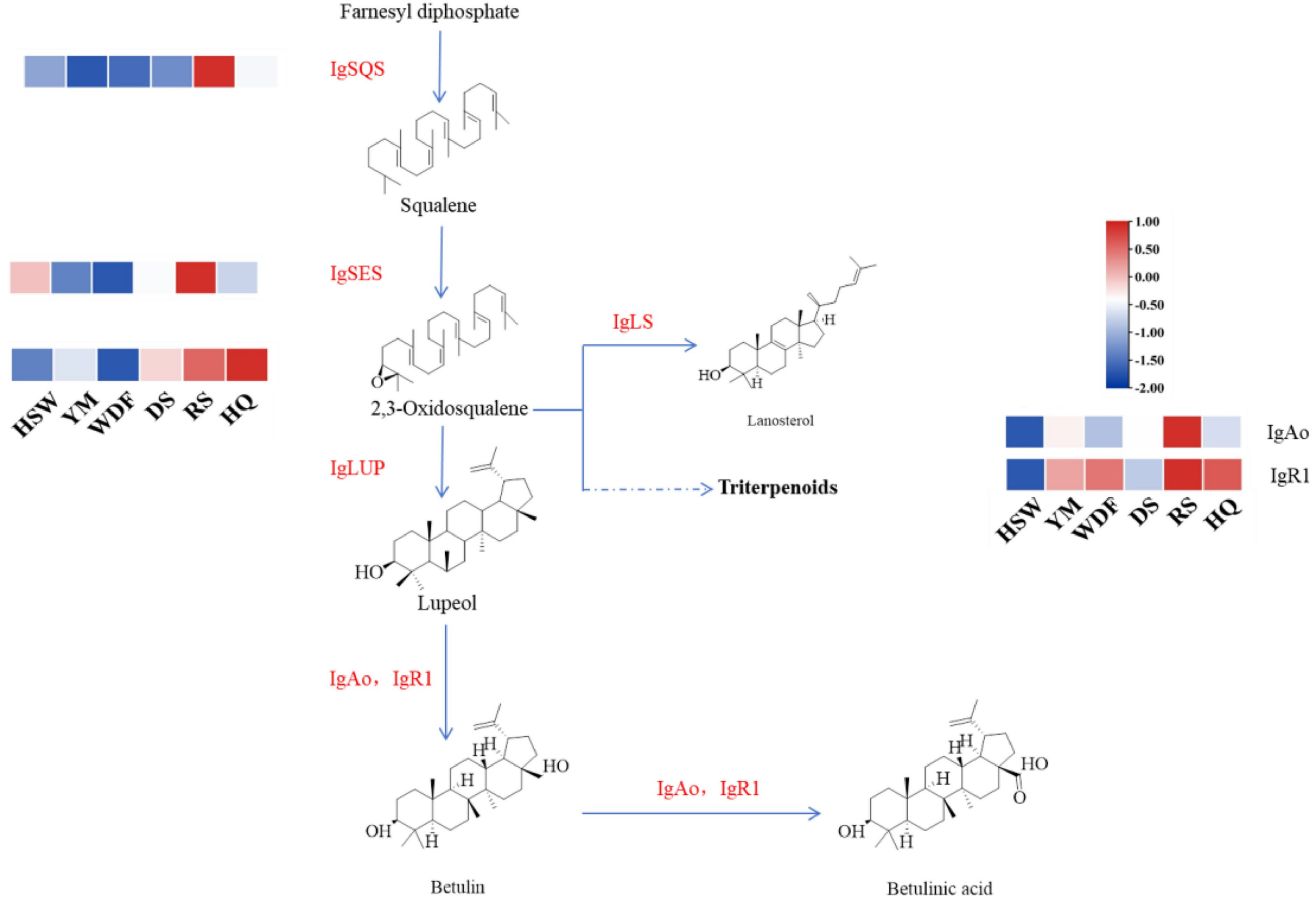
The synthesis pathway of the betulinic acid compound in *I. glomeratus*. The horizontal axis represents different culture substrates, and the vertical axis represents gene expression. The expression levels are encoded by colors, with red and blue representing high and low expression, respectively. Dashed lines represent steps made up of several enzyme reactions. SQS, squalene synthase; LS, lanosterol synthase; LUP, lupeol synthase; FPPS, farnesyl pyrophosphate synthase; SES, squalene epoxidase synthase.

Through genomic analysis, we identified key enzyme genes involved in terpene biosynthesis, including AACT, HMGS, HMGR, MVD, SQS, LS, FPPS, and SES (Supplementary Table S3). Local BLAST and conserved motif analysis showed that there are five genes are similar with CrAo (Supplementary Figure S11) and three genes is similar with known ATR1 in *I. glomeratus* genome (Supplementary Figure S12). These genes exhibited high similarity to CrAo and ATR1 have been listed in Supplementary Table S4. Based on the combined results of gene expression and metabolomics analysis, IgAo (Supplementary Figure S13) and IgR1 (Supplementary Figure S14) were further identified. Metabolome analysis detected three triterpenoid compounds with bioactive properties: betulin, betulinan C, and betulinic acid. Notably, in RS media, betulin and betulinic acid levels were the highest, exhibiting 4,658–9,275-fold and 4–503-fold increases, respectively, compared to other substrates, consistent with the gene expression trends (Figure 20). These findings suggest that differential gene expression influences the biosynthesis of betulinic acid, resulting in content variations across culture substrates. By systematically analyzing the *I. glomeratus* genome, we successfully identified the terpene synthase (TPS) gene family and constructed a phylogenetic tree. Phylogenetic analysis revealed that *IgTPS9* is within the same evolutionary branch as known squalene synthases, sharing 98% homology. Integrating multi-omics data, we hypothesize that *IgTPS9* in *I. glomeratus* encodes a functional protein with squalene synthase (SQS) activity, thereby contributing to the betulinic acid biosynthesis pathway (Figure 21). The marked differences in betulinic acid content across culture substrates and its structural characteristics are illustrated in Figure 22. In consideration of the cooperative catalysis that is a prerequisite for this biosynthetic process, it is proposed that *IgTPS9* is the catalyst for the conversion of FPPS to squalene, thereby supplying essential precursors for betulinic acid synthesis in *I. glomeratus*. Through multi-omics analysis, genes associated with the synthesis of betulinic acid derivatives were identified. However, these genes require further functional validation or heterologous expression to confirm their roles in the biosynthesis of betulinic acid derivatives.

**Figure 23:**
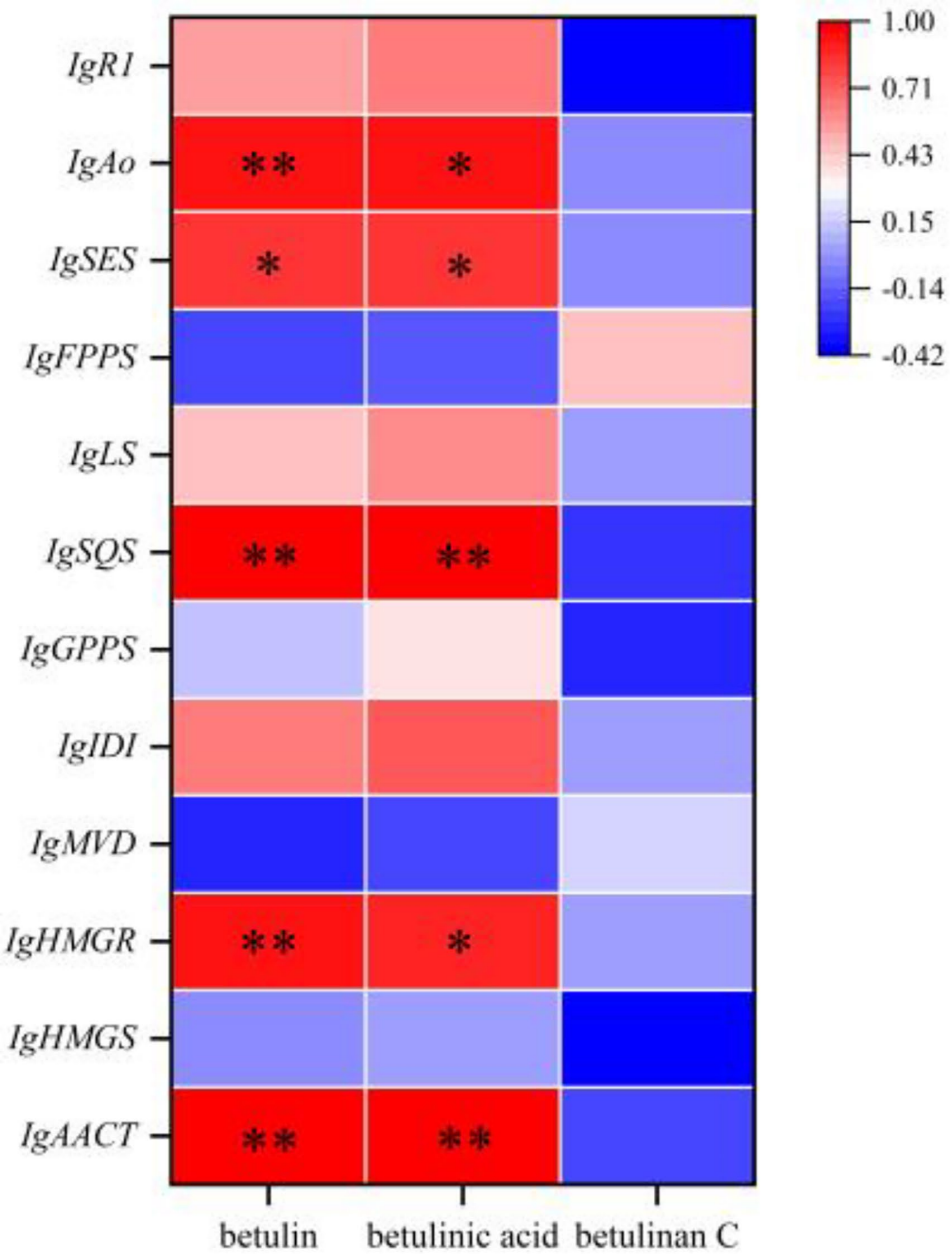
Investigation of the correlation between terpenoid biosynthetic genes and terpenoid metabolites in *I. glomeratus*.

The Pearson correlation coefficient was used to evaluate the relationships between terpenoid biosynthetic genes and terpenoid metabolites in *I. glomeratus*. The terpenoid biosynthetic genes were identified through local BLAST searches and gene annotation, while documented terpenoid metabolites previously reported in *Inonotus* were selected from the metabolomic data for comparison. Significant positive correlations were observed between betulin, betulinic acid, and the genes *IgAACT*, *IgHMGR*, *IgSQS*, *IgSES*, and *IgAo* (Figure 23). These results indicate that terpenoid biosynthetic genes play a critical role in the biosynthesis of terpenoid metabolites, particularly antcins, in *I. glomeratus*.

### 3.11 Identification and analysis of transcription factors in *Inonotus glomeratus*

**Figure 24:**
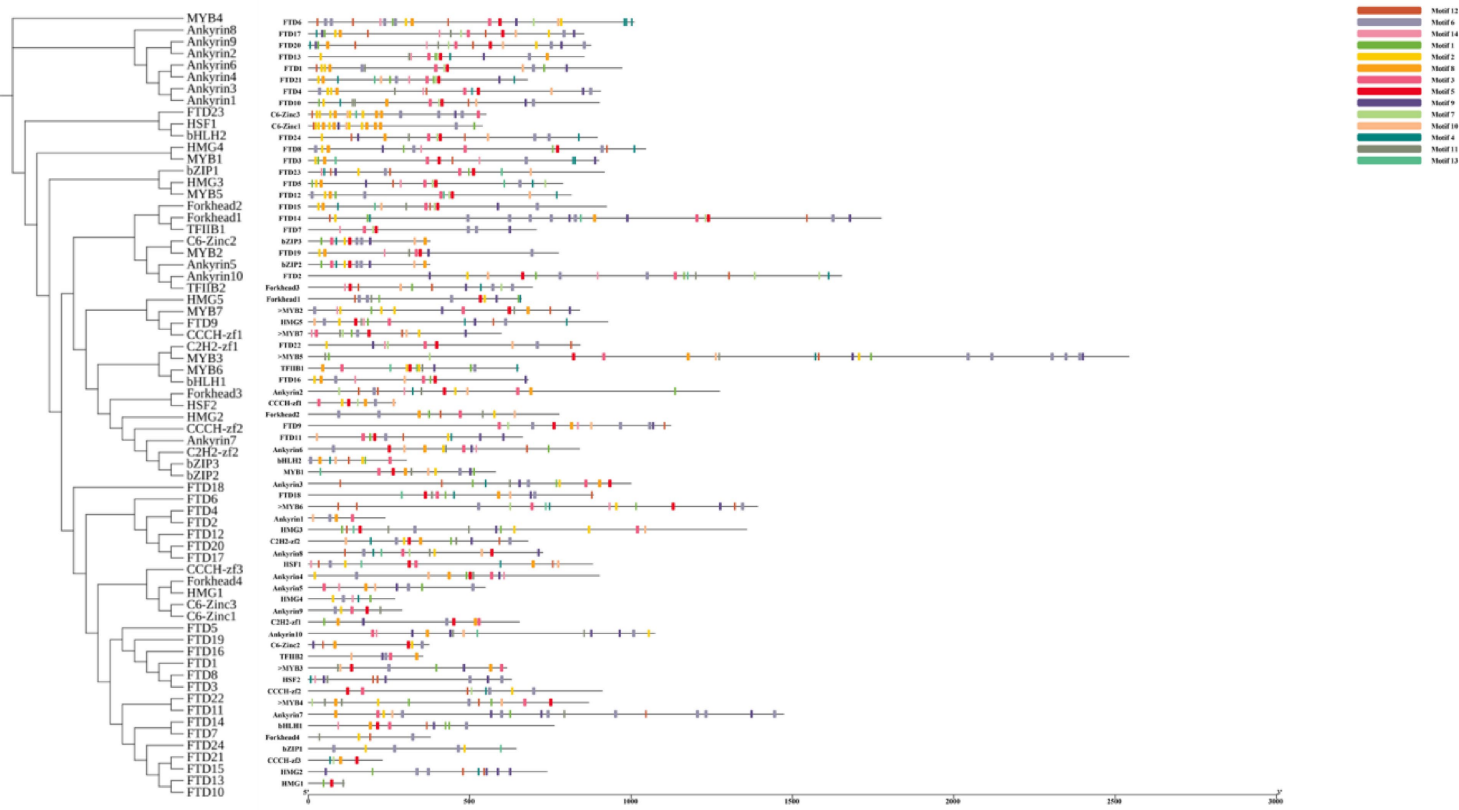
Structural characteristics of 12 transcription factor families in *I. glomeratus*. From left to right: protein phylogenetic tree, conservation motif analysis.

Through comparative analysis, we identified 67 transcription factor sequences in the *I. glomeratus* genome, which were classified into the following families: MYB (7), bZIP1 (3), bHLH (2), C2H2-zf (2), CCCH-zf (3), C6-Zinc (2), HSF (2), TFIIB (2), HMG (5), FTD (24), Forkhead (4), and Ankyrin (10). These sequences were designated as *IgMYB1* to *IgFTD24*. A phylogenetic tree of these 12 TF families was constructed using MEGA X 64 software, revealing that members within the same clade exhibited high similarity in conserved motif composition, suggesting shared functional characteristics. MEME motif analysis identified 14 distinct motifs, with phylogenetically related TFs often harboring similar motifs. Notably, all 24 *IgFTD* proteins contained motif 5, implying that this motif may represent a conserved signature of the *IgFTD* family (Figure 24). Furthermore, variations in motif composition and quantity across different TF families were observed, aligning with phylogenetic divergence. These differences may reflect functional specialization within each TF family.

### 3.12 Prediction of IgTFs binding sites in the promoter regions of IgPKS and IgTPS genes

**Figure 25:**
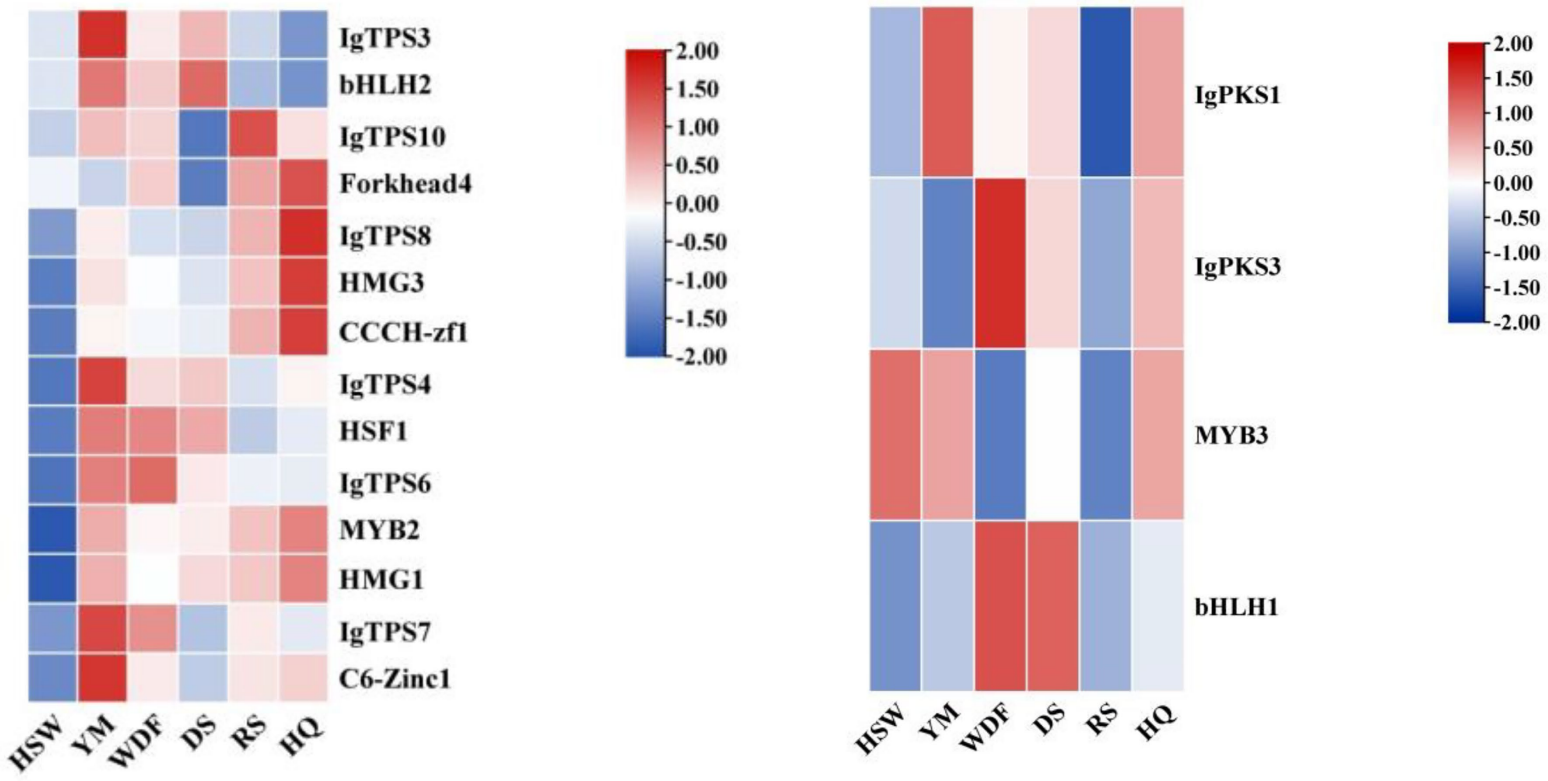
Interactive heatmap of gene expression under different culture substrates (left: *IgTPS*, right: *IgPKS*) and co-expressed *IgTFs*. The horizontal axis represents different culture substrates, and the vertical axis represents gene expression. The expression levels are encoded by colors, with red and blue representing high and low expression, respectively.

A comprehensive analysis of the expression patterns of *IgPKS*, *IgTPS*, and *IgTFs* under different culture substrates revealed that in YM media, the expression levels of *IgbHLH2*, *IgHSF1*, and *IgC6-Zinc1* were higher and closely correlated with those of *IgTPS3*, *IgTPS4*, *IgTPS6*, and *IgTPS7*. In HQ medium, the expression patterns of *IgForkhead4*, *IgHMG3*, and *IgCCCH-zf1* were similar to that of *IgTPS8*, showing a significant upward trend. Additionally, in the WDF medium, the expression patterns of *IgPKS3* and *IgbHLH1* were similar and relatively high (Figure 25). Based on these findings, it is speculated that these *IgTFs* may regulate the *IgPKS* and *IgTPS* genes. To verify this hypothesis, we conducted the following experiments. Using the complete genome data of the *I. glomeratus* fungus and the TBtools software (version 2.056), we determined the promoter regions (2,000 base pairs) of the *IgPKS* and *IgTPS* genes.

The transcription factor (TF) binding sites within the promoter regions of the *IgPKS* and *IgTPS* genes were meticulously analyzed using the JASPAR database. Several potential binding sites with relatively high scores were identified, suggesting their possible involvement in the regulation of the *IgPKS* and *IgTPS* genes. *IgMYB3* was found to bind to the negative strand of the *IgPKS1* promoter region from nucleotides 1,027 to 1,033, with the binding site sequence “tacccaa” and a relative score of 0.9060156, indicating its potential role in regulating *IgPKS1* expression. The binding sites for *IgbHLH1* and *IgbHLH2* were identified as “gcacgtgc” and “gcatgtgca,” respectively, both of which exhibited strong matches with the promoter region of *IgPKS3*. The promoter regions of *IgC3H-Zinc1* and *IgHMG3* were found to be highly similar to that of *IgTPS8*. *IgC6-Zinc1* bound to the “ctcggaaa” site in the *IgTPS7* promoter region with a relative score of 1, demonstrating high specificity. *IgHSF1* showed strong matching with the promoter regions of *IgTPS4* and *IgTPS6* (Table 5). Collectively, these findings suggest that the identified *IgTFs* may bind to specific DNA sequences within the promoter regions of the corresponding *IgPKS* and *IgTPS* genes, potentially activating their expression.

**Table 5:** Binding sites of co-expressed *IgTFs* in the promoter regions of *IgPKS* and *IgTPS*.

TF ID	Score	Relative score	Sequence ID	Start	End	Strand	Predicted sequence
IgMYB3	7.122556	0.9060156	IgPKS1	1,027	1,033	−	tacccaa
IgbHLH1	13.154779	1	IgPKS3	942	949	+	gcacgtgc
IgbHLH2	12.775514	0.9416177	IgTPS3	316	324	−	gcatgtgca
IgC3H-Zinc1	11.664035	0.95640934	IgTPS8	597	605	−	aaaaagata
IgHMG3	13.529	1	IgTPS8	666	673	−	gccgggga
IgForkhead 4	10.548165	0.9317854	IgTPS10	13	19	+	ataaaca
IgHSF1	8.239277	0.99999994	IgTPS4	362	366	−	ggcca
IgHSF1	8.197334	0.98544836	IgTPS 6	509	514	−	ggccgt
IgC6-Zinc1	12.908792	1	IgTPS 7	753	760	−	ctcggaaa
IgbHLH2	12.775514	0.9416177	IgTPS 3	316	324	−	gcatgtgca

### 3.13 Quantitative real-time polymerase chain reaction analysis

**Figure 26:**
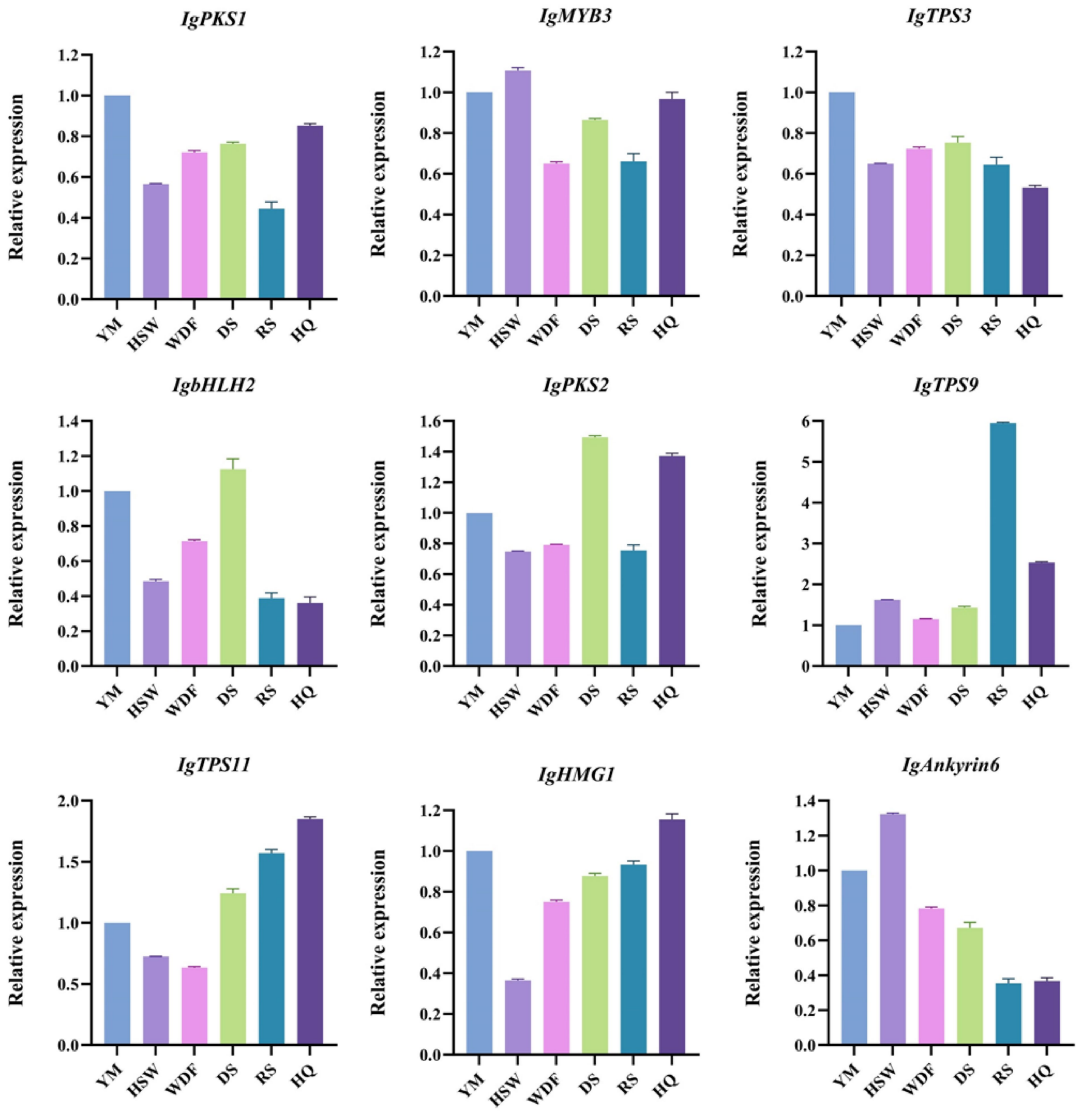
Relative expression of differentially expressed genes by qRT-PCR. The horizontal axis represents different cultivation conditions. The vertical axis represents gene expression. Bars represent the mean ± standard deviation. Processed *P. multiflorum* culture of mycelium (HSW), processed *coix seed* culture of mycelium (YM), *P. sativum* flour culture of mycelium (WDF), *S. miltiorrhiza* culture of mycelium (DS), *P. ginseng* culture of mycelium (RS), and *A. membranaceus* culture of mycelium (HQ).

The expression of nine differentially expressed genes associated with terpenoid metabolism in *I. glomeratus* was analyzed using qRT-PCR, confirming the accuracy of the transcriptome sequencing results. The results demonstrated that the relative expression levels of these nine genes aligned with the trends observed in the transcriptome data. Specifically, the relative expression levels of *IgTPS3* and *IgbHLH2* in YM, HSW, WDF, DS, and RS were comparable but significantly higher than those in HQ. In contrast, the relative expression levels of *IgTPS11* and *IgHMG1* were similar across different cultivation conditions, with HQ exhibiting significantly higher expression than the others. Meanwhile, the relative expression levels of *IgMYB3* and *IgAnkyrin6* remained consistent under different cultivation conditions, though HSW and YM showed significantly higher expression than the other groups (Figure 26). These findings suggest that cultivation conditions significantly influence the expression patterns of terpenoid biosynthesis-related genes in *I. glomeratus*. Variations in cultivation conditions may activate terpenoid metabolic pathways, thereby enhancing the synthesis of diverse bioactive terpenoids in *I. glomeratus*.

## 4 Discussion

Research has shown that *Inonotus* possesses diverse bioactive components, including polysaccharides, polyphenols, and terpenoids (Wang Z. X. et al., 2022), and exhibits multiple pharmacological activities, such as anticancer, anti-inflammatory, antioxidant, and hypoglycemic effects (Kou et al., 2021; Choi et al., 2022). Recent advancements in next-generation sequencing (NGS) technology have substantially accelerated fungal genome sequencing while reducing associated costs (Cacho et al., 2015). In this study, we integrated Oxford Nanopore Technologies (ONT) and Illumina NovaSeq sequencing data to successfully assemble the complete genome of *I. glomeratus*. The average size of most fungal genomes is approximately 40 Mb, and the assembled *I. glomeratus* genome (38.68 Mb) aligns with expectations based on the closely related *Sanghuangporus sanghuang* genome (34.5 Mb). The GC content was determined to be 47.98%, and 8,944 protein-coding genes were predicted. Notably, we identified 297 carbohydrate-active enzyme (CAZyme) genes, with a significant enrichment in lignin-degrading enzymes (e.g., laccases and manganese peroxidases), consistent with its biological role as a white-rot fungus. This functional adaptation enables *I. glomeratus* to efficiently degrade lignocellulose, contributing significantly to carbon cycling in forest ecosystems.

### 4.1 Effects of matrix on metabolite diversity

Changes in growth conditions can stimulate the production of fungal secondary metabolites (Li and Lou, 2018). For instance, oleic acid and fungal elicitors significantly enhance betulinic acid biosynthesis in *I. obliquus*. HPLC analysis revealed that the betulinic acid content in dried mycelium and fermentation broth peaked at an oleic acid concentration of 1.0 g/L, exhibiting an approximately 2-fold increase compared to the control group (Lou et al., 2021). Similarly, the traditional Chinese medicine “*S. sanghuang*” demonstrated increased flavonoid and phenolic compound yields following a two-stage culture strategy involving oscillation-static incubation. Specifically, total flavonoid and total phenol contents in the mycelium increased by 37.92 and 77.27%, respectively (Tian et al., 2015). In this study, metabolomic analysis under varying culture substrate conditions identified a total of 2,324 differentially abundant metabolites. Lipids and lipid-like molecules exhibited the highest content across all culture substrates. Notably, sesquiterpene content was highest in HSW, YM, WDF, and HQ medium. Previous studies have demonstrated that terpenoids and alkaloids are among the secondary metabolites present in *P. multiflorum*, *C. lacryma-jobi*, *S. miltiorrhiza*, *P. ginseng*, and *A. membranaceus* substrates (Murthy et al., 2024; Yin et al., 2018; Zheng et al., 2023). Notably, several terpenoid active compounds found in Chinese medicines were also identified in our metabolomic profiles. These include *P. ginseng* (e.g., ginsenoside Rd, Re, Rg2, Rg3, Rh1, and Rh2) and diterpene quinones from *S. miltiorrhiza* (e.g., tanshinone I, tanshinone IIA, and tanshinone IIB). Furthermore, flavonoids such as naringenin and quercetin, associated with *A. membranaceus*, were also detected. Therefore, there is a certain correlation between these additives and terpenoid content. The composition and biological activity of *Inonotus* metabolites are associated with different culture media. By optimizing the culture substrate, the production of target compounds, such as polyphenols and triterpenoids, can be enhanced (Javahershenas and Nikzat, 2024). This approach is crucial for developing *Inonotus*-based products with specific biological functions.

Studies have confirmed that microbial solid-state fermentation substantially enhances bioactive compounds in Chinese herbal medicines. For instance, lactic acid bacteria fermentation significantly elevated flavonoid and polyphenol levels in *Artemisia argyi* leaves (Zhou et al., 2025), while optimized solid-state fermentation conditions markedly increased polyphenol extraction from *Acanthopanax senticosus* (Su et al., 2023). Additionally, medicinal fungi fermentation has yielded several bioactive triterpenoids (Zhao et al., 2023). In this study, six Chinese herbal medicines were employed as the primary constituents of solid culture substrates to cultivate *I. glomeratus*. The metabolomic analysis revealed that different herbal matrices significantly influenced the production of secondary metabolites. Specifically, terpenoids (4- to 32-fold increase) and diterpenoids (124- to 331-fold increase) were markedly elevated in the DS medium compared to other groups, consistent with the abundance of diterpene-quinone secondary metabolites in *S. miltiorrhiza*. Notably, solid-state fermentation may enhance the biosynthesis of specific bioactive compounds (e.g., tanshinones, diterpene quinones, and phenolic acids) by modulating the metabolic pathways of *S. miltiorrhiza* (Zhang et al., 2014; Naz et al., 2020). The RS medium exhibited the most pronounced increases in sesquiterpenoids (1- to 2-fold) and triterpenes (8- to 56-fold), aligning with the predominance of *P. ginseng* triterpenoid saponins as its active constituents (Han and Zhong, 2003; Chen et al., 2019). For instance, *Armillaria mellea* has been observed to convert ginsenoside Rb2 into the rare ginsenosides C-Y and C-K via the pathway Rb2 → C-Y → C-K (Kim et al., 2018), suggesting that fungal biotransformation exerts a specific effect on the RS substrate and further influences its metabolic flux. The HQ medium significantly enhanced flavonoid accumulation. This observation aligns with the flavonoid-rich properties of *A. membranaceus* and further underscores the regulatory role of substrate composition in metabolite production (Xia et al., 2014; Liu et al., 2021). The results of this study showed that the production of terpenoids and flavonoids was significantly promoted in RS, DS, and HQ media, but the mechanism of its effect on the fermentation process was still unclear. Some of the terpenoids in *S. miltiorrhiza*, *P. ginseng*, and *A. membranaceus* may be involved in the terpenoid metabolism of *I. glomeratus*. The type, structure, and transformation process of these substances in terpenoid metabolism are the focus of the next research.

### 4.2 Genomics and transcriptomics insights

Fungal secondary metabolites (SMs) play vital roles in biomedicine, biocontrol, and the food industry, with their biosynthetic processes primarily governed by the catalytic activity of PKS or TPS (Vassaux et al., 2019). The secondary metabolite analysis tool antiSMASH facilitates the rapid and direct identification of biosynthetic gene clusters (BGCs) and their associated gene cluster families. These metabolites are synthesized by PKSs, non-ribosomal peptide synthases (NRPSs), and TSs (Keller, 2019). In this study, we performed genome mining on the fungus *I. glomeratus* to characterize its secondary metabolite biosynthesis gene clusters. AntiSMASH analysis revealed 16 secondary metabolite gene clusters in the *I. glomeratus* genome. In *Aspergillus nidulans*, the gene cluster F9775, responsible for orsellinic acid synthesis, encodes a PKS with the domain architecture SAT-KS-AT-PT-ACP-ACP-ACP-TE (Sanchez et al., 2010). Here, we identified *IgPKS1*, which exhibits high similarity to PKSs in *I. hispidus* (NPCB001, MA, NCMCCNO230172-1, NCMCCNO230172-15), *I. obliquus* (CT5), and *I. vitis* (OC1), all sharing identical domain structures. Notably, the regulatory gene associated with *IgPKS1* differed from that of F9775, suggesting species-specific variations in orsellinic acid biosynthesis gene clusters. PKSs involved in orsellinic acid synthesis are widely distributed across fungal species and yield diverse derivatives. In this study, we detected zearalenone, an orsellinic acid derivative, and previous studies have demonstrated the essential role of genes in zearalenone biosynthesis (Kim et al., 2005; Gaffoor and Trail, 2006). Flavonoids were originally considered characteristic secondary metabolites exclusive to higher plants (Panche et al., 2016); however, emerging evidence demonstrates that fungi also possess the capacity for flavonoid biosynthesis. For instance, only three flavonoid-related enzymes were annotated in *Auricularia cornea* (Meng et al., 2022), whereas 81 flavonoids were detected in *S. baumii*, despite the identification of merely four biosynthetic enzymes (Wang S. et al., 2022). Similarly, *Stropharia rugosoannulata* was found to harbor 59 structural genes encoding flavonoid biosynthesis-related enzymes (Wu et al., 2024). In this study, *I. obliquus* (CT5), *I. hispidus* (NPCB001), *I. vitis* (OC1), and *I. obliquus* (CFCC83414) all exhibited proteins homologous to *IgPKS2*, featuring the PKS structural domains AMP-ACP-KS-AT-DH-KR-ACP-ACP. Phylogenetic analysis revealed that *IgPKS2* clustered with these four PKS genes into a monophyletic clade with high sequence homology. Furthermore, gene deletions, horizontal gene transfer events within each, and the variability of adjacent modifier genes underscored flavonoid diversity and indicated the coexistence of conserved and divergent biosynthetic pathways. Given that *IgPKS2* in the comparative genomic analysis primarily contributed to flavonoid production, we hypothesize that its principal function involves naringenin or derivative synthesis. Additionally, the expansion of secondary metabolite synthesis-related genes (e.g., PKS) suggests potential for novel bioactive compound production, warranting further exploration for pharmaceutical applications.

Phylogenetic analysis and molecular evolutionary tree construction of the *I. glomeratus* TPS gene family revealed that *IgTPS9* and squalene synthase (SQS) cluster into a single evolutionary unit, suggesting that *IgTPS9* may participate in the betulinic acid biosynthetic pathway. Previous studies have elucidated key steps in the biosynthesis of betulinic acid, a highly bioactive triterpenoid primarily synthesized via the mevalonate pathway (MVA pathway) (Quin et al., 2014). Notably, significant progress has been made in studying betulinic acid biosynthesis in *Saccharomyces cerevisiae*. In this pathway, 2,3-oxidized squalene is first synthesized through the MVA pathway (Meunier et al., 2004), followed by cyclization into lupeol catalyzed by LUPase. Subsequently, lupeol is oxidized by CrAO and ATR1 to yield betulinic acid and betulin (Li et al., 2025; Tang et al., 2024). In this study, integrated multi-omics analysis successfully identified key genes involved in betulinic acid biosynthesis. Local BLAST comparisons further confirmed the presence of two critical enzymes, CrAo and ATR1, in *I. glomeratus* (designated *IgAo* and *IgR1*, respectively; Supplementary Table S4). Additionally, several genes encoding pivotal biosynthetic enzymes, including HMGR and SQS, were cloned from *I. obliquus* (Zhang et al., 2016). Gene expression profiling demonstrated significant upregulation of key enzyme genes (*IgAACT*, *IgHMGR*, *IgSQS*, *IgSES*, *IgAo*, *IgR1*, and *IgTPS9*) in RS medium. Metabolomic analysis corroborated these findings: under the same substrates, betulin and betulinic acid levels were markedly higher than in other treatment groups (*p* < 0.05). Specifically, betulin accumulation reached 4,658–9,275-fold that of other conditions, while betulinic acid levels increased by 4–503-fold. These results not only confirm a strong correlation between gene expression patterns and metabolite accumulation but also provide a robust foundation for elucidating the molecular mechanisms underlying betulinic acid biosynthesis. It is worth noting that betulin, a direct precursor of betulinic acid, has garnered increasing attention in recent years due to its biotransformation potential (Chen et al., 2009; Wu et al., 2017; Kumar and Dubey, 2017). Several studies have demonstrated that fatty acids can enhance mycelial growth in medicinal fungi such as *Ganoderma lucidum* (Yang et al., 2000) and significantly boost secondary metabolite production, including triterpenoids (Yang et al., 2013). A related study identified pH 6.2 and 28 °C as optimal growth conditions for *I. obliquus* in liquid medium when supplemented with different betulin sources (Fradj et al., 2019). These insights offer crucial theoretical support for regulating betulinic acid biosynthesis.

Secondary metabolite biosynthetic gene clusters (SMBGCs) in fungi can be activated through modifications in culture substrates or genetic manipulation. Both the choice of media and cultivation methods significantly influence the growth rate and secondary metabolite (SM) production in *Inonotus* (Bai et al., 2012). Previous studies have demonstrated that woody substrates substantially alter the expression levels of terpene biosynthesis-related genes in *Inonotus* mycelia. For instance, co-culturing *I. obliquus* with *Phellinus punctatus* reduces mycelial biomass but enhances the accumulation of phenolic compounds, melanin, and lanosteroidal triterpenoids (Nazloo et al., 2024). In this study, the addition of YM medium led to significant upregulation of *IgPKS1*, *IgTPS1*, *IgTPS3*, *IgTPS4*, *IgTPS7*, and *IgC6-Zinc1*. Conversely, DS medium notably increased the expression of *IgPKS2* and *IgbHLH1*. Under RS medium conditions, *IgTPS2*, *IgTPS10*, *IgTPS9*, and five *IgTFs* were highly expressed, whereas HQ medium induced significant expression of *IgTPS8* and nine *IgTFs*.

Transcription factors (TFs) are pivotal proteins that modulate gene expression and play an essential role in the regulatory network governing fungal secondary metabolism (Calvo et al., 2024). For instance, in *Aspergillus fumigatus*, the induction of *apdR* expression activated a NRPS-PKS heterotrimeric biosynthesis gene cluster, leading to the production of aspyridones exhibiting moderate cytotoxic activity (Bergmann et al., 2007). Transcriptome analysis has demonstrated that the Ypr1 transcription factor regulates fungal secondary metabolism (Zhou et al., 2021), while the AflRsmA transcription factor in *Aspergillus flavus* influences aflatoxin B1 biosynthesis, oxidative stress response, and sclerotial formation (John et al., 2022). These findings align with our observations. In this study, we integrated co-expression patterns under varying substrate culture conditions with transcription factor binding site predictions, hypothesizing that *IgMYB* may directly regulate *IgPKS1* expression by binding to its promoter region. Additionally, *IgPKS3* expression may be modulated by *IgbHLH1* and *IgbHLH2*, whereas *IgC3H-Zinc1* and *IgHMG3* may negatively regulate *IgTPS8*. Furthermore, *IgHSF1* potentially regulates both *IgTPS4* and *IgTPS6*. These results suggest that these TFs may enhance the transcription of terpene synthase (TPS) genes, thereby promoting TPS synthesis and subsequent sesquiterpene production. Although our multi-omics analysis showed that *IgMYB3* may regulate *IgPKS1* expression and *IgHSF1* may be involved in the transcriptional regulation of *IgTPS4* and *IgTPS6*, these links are currently only derived from the bioinformatics prediction of co-expression networks and cis-elements, and there is still no experimental evidence. In the next stage, relevant research will continue to be carried out to transform computational predictions into experimentally verified regulatory pathways.

### 4.3 Limitation and prospect

Basidiomycetes exhibit distinct gene expression profiles at different developmental stages, resulting in variations in the types and concentrations of terpenoids and other bioactive compounds produced (Vonk and Ohm, 2021). Therefore, further investigation into the gene expression and metabolic pathways of *I. glomeratus* at different developmental stages may help elucidate the transcriptional regulatory mechanisms underlying the biosynthesis of its active metabolites. Specifically, untargeted metabolomics faces challenges in compound identification due to incomplete reference databases, the presence of isomers, and isomerization phenomena. Moreover, it cannot precisely quantify absolute compound concentrations, as it only allows for the comparison of relative abundances across samples, which compromises both data accuracy and reproducibility (Beger et al., 2019). In contrast, targeted metabolomic analysis of individual compounds extracted and purified from *I. glomeratus* provides a more accurate approach, enabling the determination of absolute concentrations. Furthermore, the terpenoids biosynthetic genes identified in this study may be functionally validated in future work and heterologously expressed in engineered microbial systems to enhance the production of bioactive terpenoids from *I. glomeratus*.

## 5 Conclusion

This study employed an integrated multi-omics approach, combining genomics, metabolomics, and transcriptomics, to investigate the regulatory mechanisms underlying secondary metabolite biosynthesis in *I. glomeratus* under six distinct culture substrate conditions. The *I. glomeratus* genome (38.68 Mb, comprising 23 scaffolds) was assembled using Illumina and Nanopore sequencing technologies, which revealed key functional genes, including 67 transcription factors, four PKS, one NRPS, and 11 TPS. Multi-omics analysis delineated specific biosynthetic pathways: *IgPKS1* is potentially involved in orsellinic acid and its derivative biosynthesis, *IgPKS2* may contribute to naringenin production, *IgTPS9* is associated with betulinic acid biosynthesis, and *IgTPS10* participates in tetracyclic sesquiterpene alkene B-type triterpene formation. Co-expression network analysis of the promoter regions of *IgPKS* and *IgTPS* genes, along with transcription factor binding site predictions, suggested that *IgMYB3* regulates *IgPKS1* expression, while *IgHSF1* may modulate the expression of both *IgTPS4* and *IgTPS6*. This study not only enhances the genetic understanding of *I. glomeratus* but also provides valuable insights into the genomic characteristics of fungi within the *Inonotus* genus.

## Data Availability

The genome sequence of *Inonotus glomeratus* has been deposited at NCBI with accession number SAMN40976063, BioProject number PRJNA1100642 (GenBank accession number JBCEZR010000000) https://www.ncbi.nlm.nih.gov/datasets/genome/GCA_040938115.1/ accessed on 6 June 2025.

## Footnotes

^1^ https://www.girinst.org/repbase

^2^ http://www.hmdb.ca

^3^ http://www.massbank.jp/

^4^ http://www.lipidmaps.org

^5^ https://www.mzcloud.org

^6^ https://antismash.secondarymetabolites.org/

^7^ http://www.softberry.com/

^8^ http://nrps.igs.umaryland.edu/

^9^ https://blast.ncbi.nlm.nih.gov/

^10^ http://iqtree.cibiv.univie.ac.at/
