# Supplementary material for: Multi-omics reveals the regulatory effects of Chinese herbal medicine substrates on secondary metabolite biosynthesis in Inonotus glomeratus
Source: Front Microbiol. 2025 Nov 7;16:1658730. doi: 10.3389/fmicb.2025.1658730 (PMC12634532; doi:10.3389/fmicb.2025.1658730)
Supplement: Supplementary file 1 [file Supplementary_file_1.docx]

Supplementary material

FIGURE S1

Functional annotation of proteins encoded by *Inonotus glomeratus* fungal genes: (a), KOG analysis; (b), GO analysis.

FIGURE S2

Analysis of differentially expressed metabolites (DEMs) of *I. glomeratus* under different culture substrate conditions. The horizontal axis represents different culture substrates and the vertical axis represents the number of differentially expressed metabolites.

FIGURE S3

KEGG pathway of differential metabolite enrichment of *I. glomeratu*s cultured in DS, HQ and HSW medium. The horizontal axis represents −log10 (*p*-value). The vertical axis represents the KEGG pathway.

FIGURE S4

Expression of secondary metabolites in each sample of *I. glomeratus* in different culture substrates. Mean ± SD (n = 3) was used, and similar letters inside the same treatment are statistically equivalent at p 0.05, based on Tukey's multiple range test. The horizontal axis represents the metabolite types: alkaloids (alkaloids and derivatives), terpenoids (terpenoids), lipids and lipid-like molecules (lipids and lipid-like molecules), organic heterocyclic compounds (organoheterocyclic compounds), phenylpropanoids, and polyketides. The vertical axis represents the metabolite content.

FIGURE S5

KEGG pathway enrichment analysis of the DEGs and DEMs of *I. glomeratus.* The horizontal axis represents the load values of genes and metabolites, while the vertical axis represents the names of metabolites or genes. It shows the top 15 genes and the top 10 metabolites ranked by their absolute load values; orange represents genes, and yellow represents metabolites.

FIGURE S6

Integrated metabolomics and transcriptomics analysis of *I. glomeratus.* The horizontal axis represents the fold change of genes, and the vertical axis represents the fold change of metabolites. The dotted line marks the position of |Log2FC>1|. From left to right and top to bottom, they are divided into 1-9 quadrants in sequence. Note: Black: Neither genes nor metabolites are differentially expressed. The genes and metabolites in this differential group are not differentially expressed; Left diagonal: Genes and metabolites have the same differential expression pattern, and for the genes and metabolites with consistent regulatory trends, the change of metabolites may be positively regulated by genes; Right diagonal: Genes and metabolites have the same differential expression pattern, and for the genes and metabolites with inconsistent regulatory trends, the change of metabolites may be negatively regulated by genes; Red, green: Metabolites remain unchanged, while genes are up- or down-regulated, or genes remain unchanged while metabolites are up- or down-regulated.

FIGURE S7

Correlation heatmap of *I. glomeratus*. Main area of the heatmap: The horizontal axis represents genes, and the vertical axis represents metabolites; the darker the color, the higher the positive correlation between the gene and the metabolite; conversely, the bluer the color, the stronger the negative correlation between the gene and the metabolite; the asterisk indicates a significant p-value less than 0.05. Metabolite clustering tree: Clustering situation of metabolites; Genetic clustering tree: Genetic clustering situation; Gene Clusters: Gene clustering classification labels. Genes with similar correlation values are clustered together as one cluster. Regulation: Information on the upregulation and downregulation of genes and metabolites in each comparison group.

FIGURE S8

Correlation network diagram of *I. glomeratus*. The color of the lines represents the magnitude of the correlation coefficient, the thickness represents the p-value size, and the shape of the nodes represents the genes and metabolites.

FIGURE S9

Correlation string graph of *I. glomeratus*. On the left is the name of the metabolite, and on the right is the name of the gene. Red indicates a positive correlation, and blue indicates a negative correlation.

FIGURE S10

Construction of a PKS phylogenetic tree for the fungus *I. glomeratus*.

FIGURE S11

Conserved motif analysis of the betulinic acid synthesis pathway gene *IgAo* in *I. glomeratus*.

FIGURE S12

Conserved motif analysis of the betulinic acid synthesis pathway gene *IgR1* in *I. glomeratus*.

FIGURE S13

Heatmap of the expression of the betulinic acid synthesis pathway gene *IgAo* in *I. glomeratus* under different culture substrates. *IgAo* was obtained by native blast. The horizontal axis indicates different culture substrates and the vertical axis indicates gene expression. Expression levels are color-coded, with red and blue indicating high and low expression, respectively.

FIGURE S14

Heatmap of the expression of the betulinic acid synthesis pathway gene *IgR1* in *I. glomeratus* under different culture substrates. *IgR1* was obtained by native blast. The horizontal axis indicates different culture substrates and the vertical axis indicates gene expression. Expression levels are color-coded, with red and blue indicating high and low expression, respectively.

TABLE S1

Primer list of genes for qRT-PCR.

TABLE S2

Genomic characterization of seven strains of the genus *Inonotus.*

TABLE S3

The identity and similarity between betulinic acid biosynthesis enzymes of *I. glomeratus* and *S. baumii.*

TABLE S4

The putative CrAo and ATR1 genes in betulinic acid biosynthesis of *I. glomeratus*.


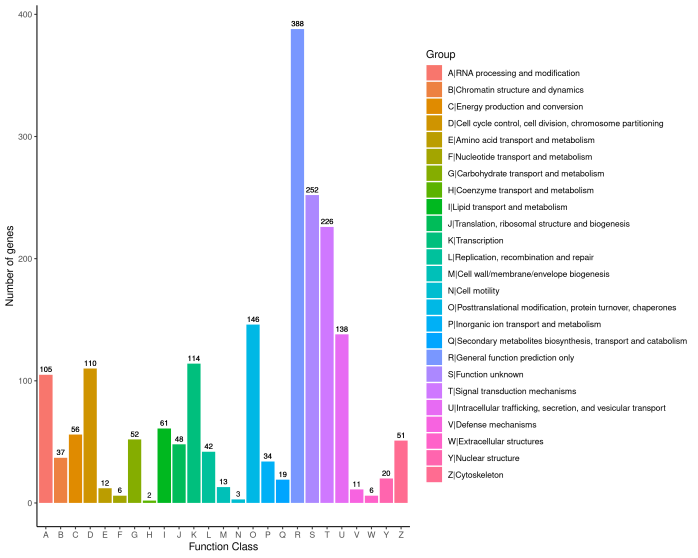

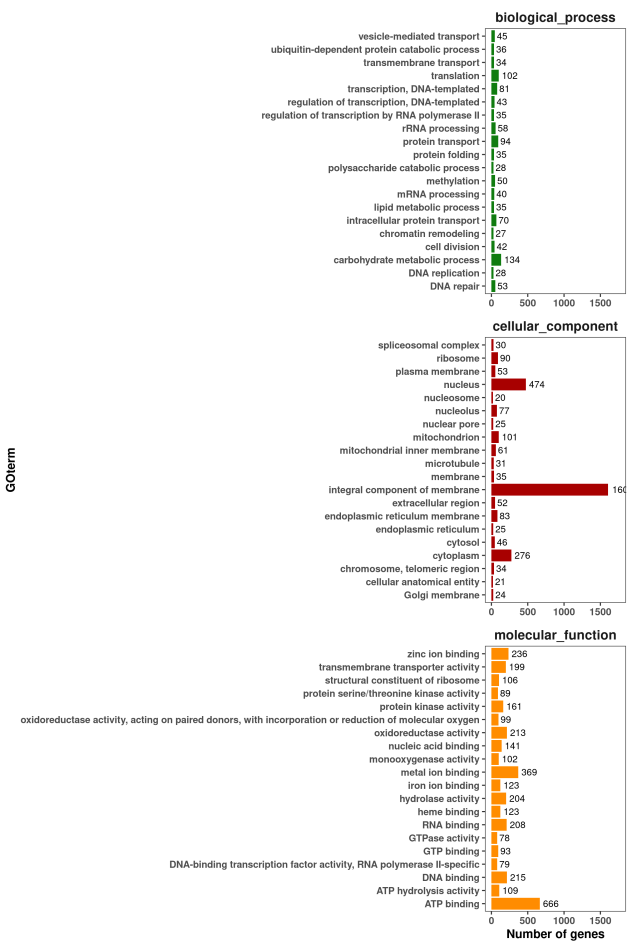


(a) (b)

FIGURE S1

Functional annotation of proteins encoded by *Inonotus glomeratus* fungal genes: (a), KOG analysis; (b), GO analysis.


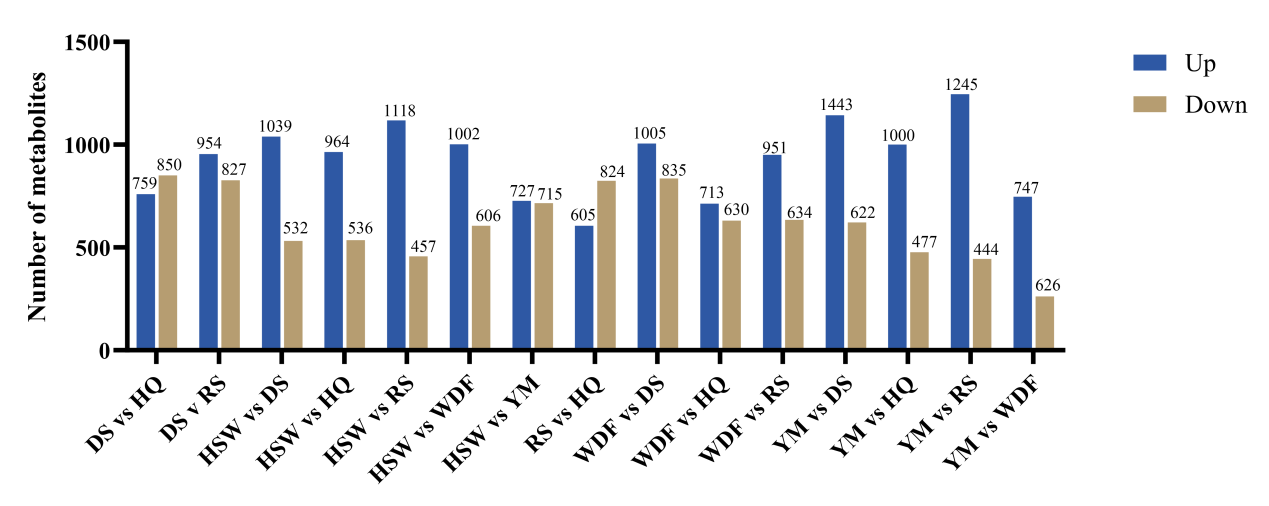


FIGURE S2

Analysis of differentially expressed metabolites (DEMs) of *I. glomeratus* under different culture substrate conditions. The horizontal axis represents different culture substrates and the vertical axis represents the number of differentially expressed metabolites.


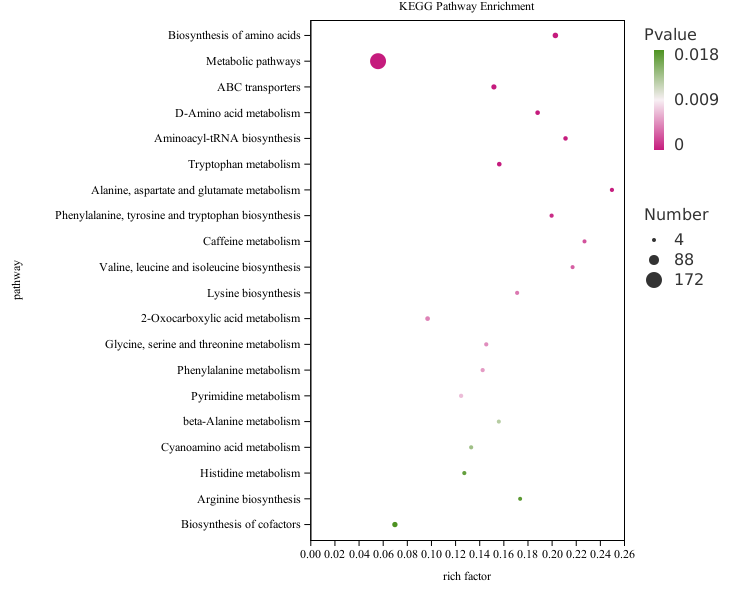


FIGURE S3

KEGG pathway of differential metabolite enrichment of *I. glomeratus* cultured in DS , HQ and HSW medium. The horizontal axis represents −log10 (*p*-value). The vertical axis represents the KEGG pathway.


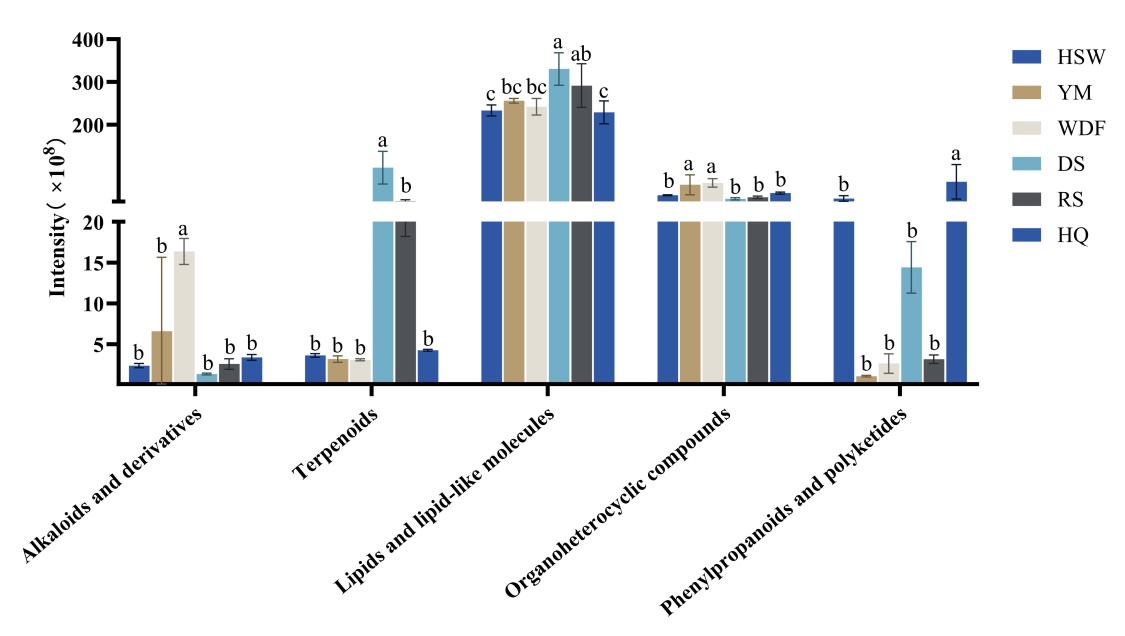


FIGURE S4

Expression of secondary metabolites in each sample of *I. glomeratus* in different culture substrates. Mean ± SD (n = 3) was used, and similar letters inside the same treatment are statistically equivalent at p 0.05, based on Tukey's multiple range test. The horizontal axis represents the metabolite types: alkaloids (alkaloids and derivatives), terpenoids (terpenoids), lipids and lipid-like molecules (lipids and lipid-like molecules), organic heterocyclic compounds (organoheterocyclic compounds), phenylpropanoids, and polyketides. The vertical axis represents the metabolite content.


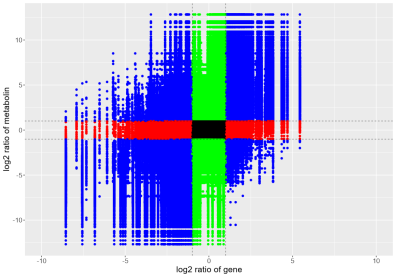

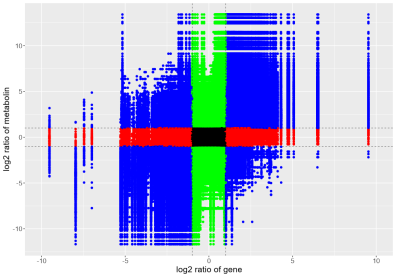

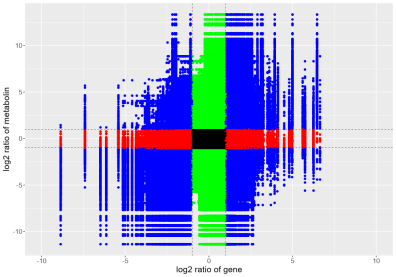


DS vs HQ DS vs RS HSW vs DS


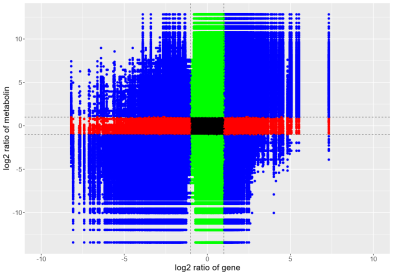

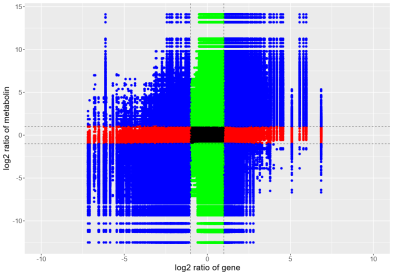

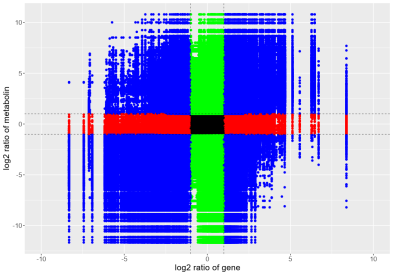


HSW vs HQ HSW vs RS HSW vs WDF


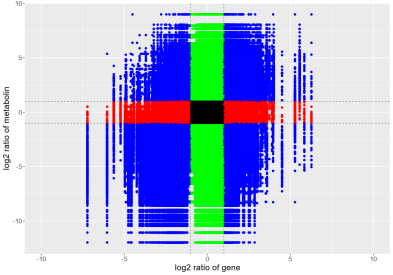

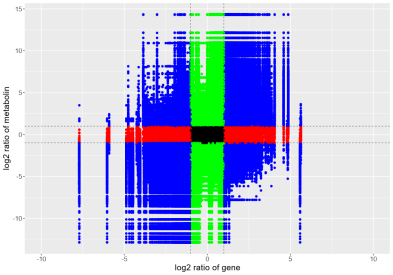

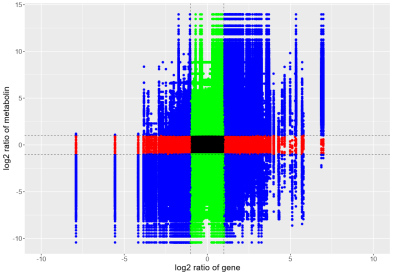


HSW vs YM RS vs HQ WDF vs DS


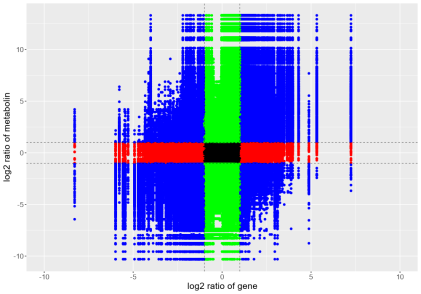

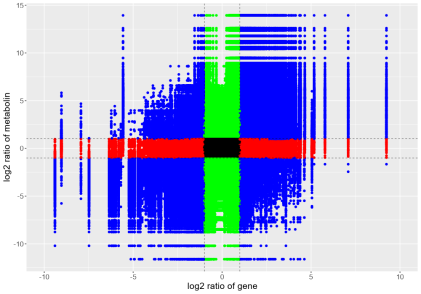

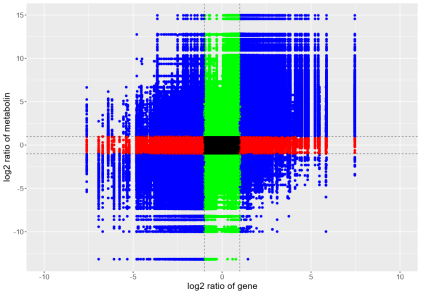


WDF vs HQ WDF vs RS YM vs HQ


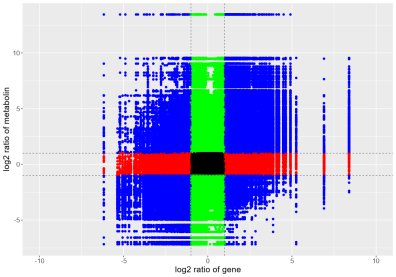


YM vs WDF

FIGURE S5

Integrated metabolomics and transcriptomics analysis of *I. glomeratus.* The horizontal axis represents the fold change of genes, and the vertical axis represents the fold change of metabolites. The dotted line marks the position of |Log2FC>1|. From left to right and top to bottom, they are divided into 1-9 quadrants in sequence. Note: Black: Neither genes nor metabolites are differentially expressed. The genes and metabolites in this differential group are not differentially expressed; Left diagonal: Genes and metabolites have the same differential expression pattern, and for the genes and metabolites with consistent regulatory trends, the change of metabolites may be positively regulated by genes; Right diagonal: Genes and metabolites have the same differential expression pattern, and for the genes and metabolites with inconsistent regulatory trends, the change of metabolites may be negatively regulated by genes; Red, green: Metabolites remain unchanged, while genes are up- or down-regulated, or genes remain unchanged while metabolites are up- or down-regulated.


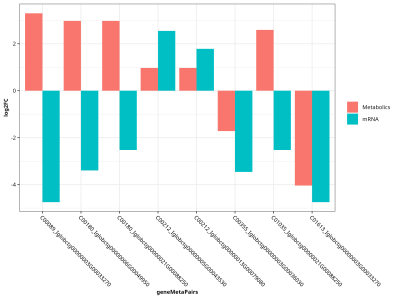

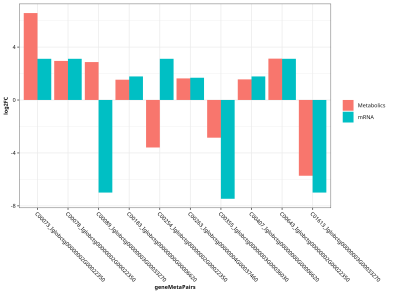

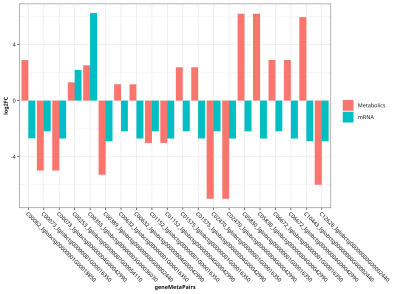


DS vs HQ DS vs RS HSW vs DS


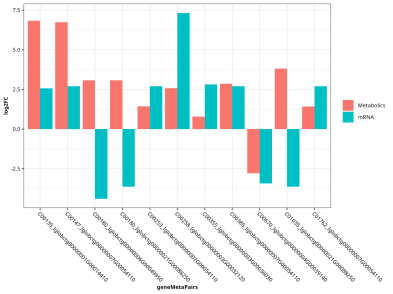

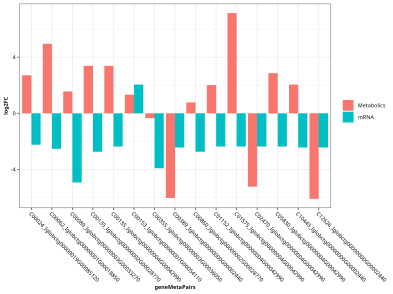

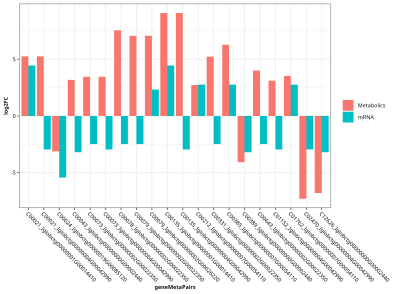


HSW vs HQ HSW vs RS HSW vs WDF


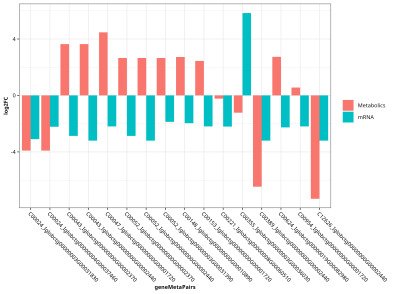

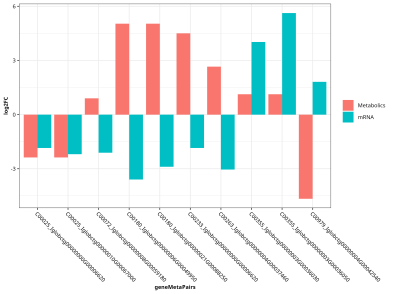

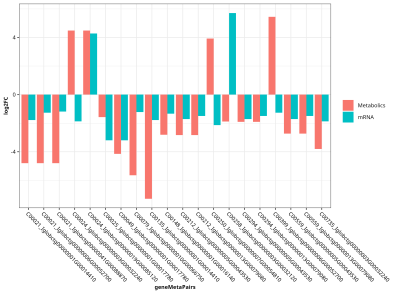


HSW vs YM RS vs HQ WDF vs DS


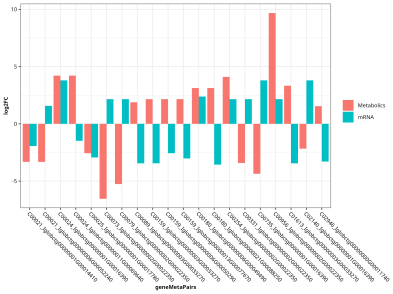

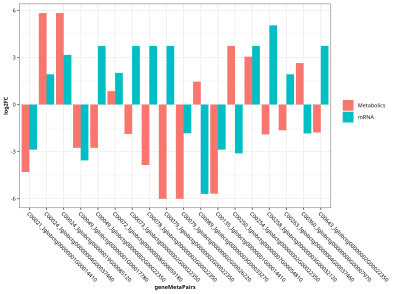

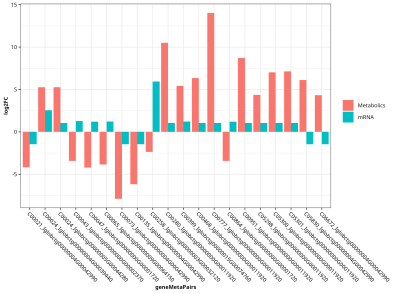


WDF vs HQ WDF vs RS YM vs DS


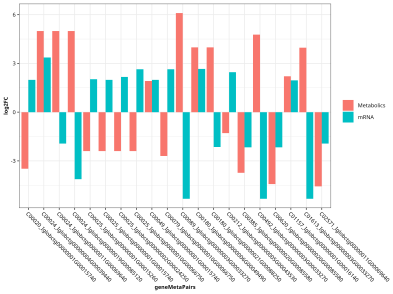

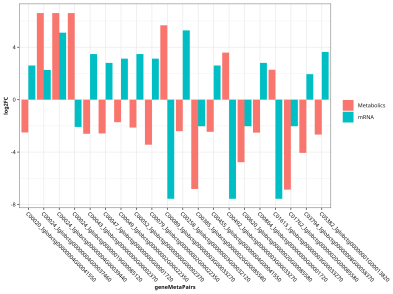

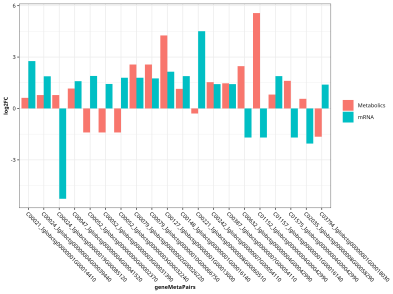


YM vs HQ YM vs RS YM vs WDF

FIGURE S6

KEGG pathway enrichment analysis of the DEGs and DEMs of *I. glomeratus*. The horizontal axis represents the load values of genes and metabolites, while the vertical axis represents the names of metabolites or genes. It shows the top 15 genes and the top 10 metabolites ranked by their absolute load values; orange represents genes, and yellow represents metabolites.


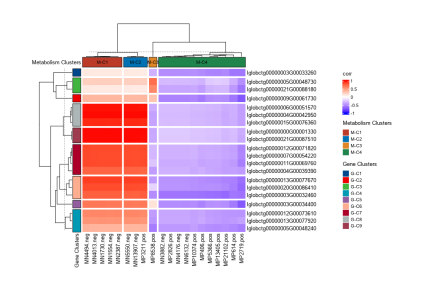

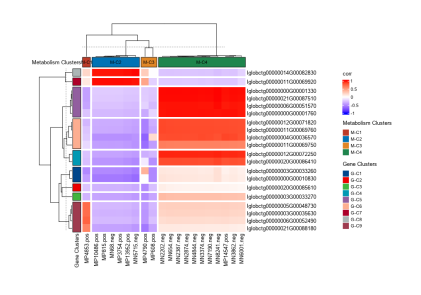

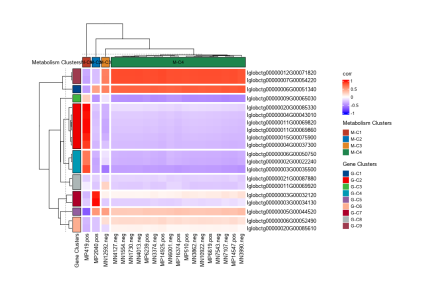


DS vs HQ DS vs RS HSW vs DS


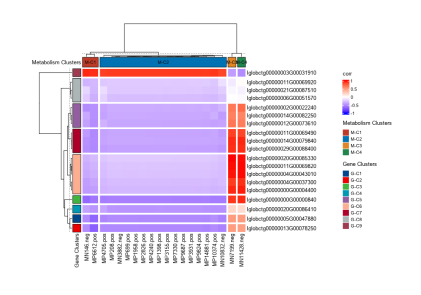

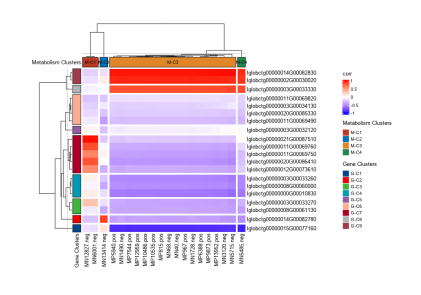

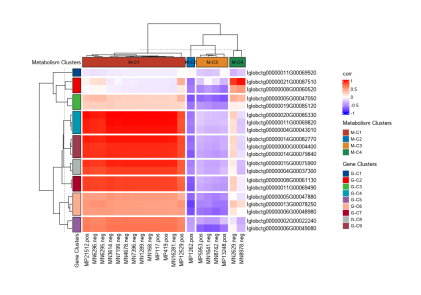


HSW vs HQ HSW vs RS HSW vs WDF


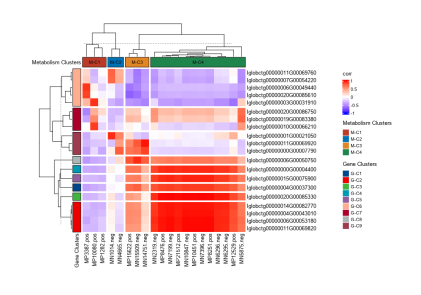

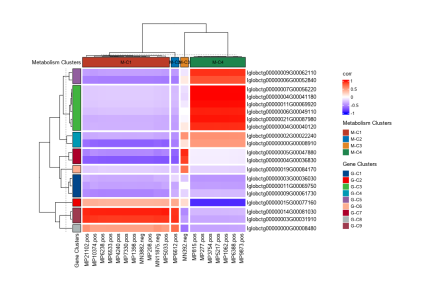

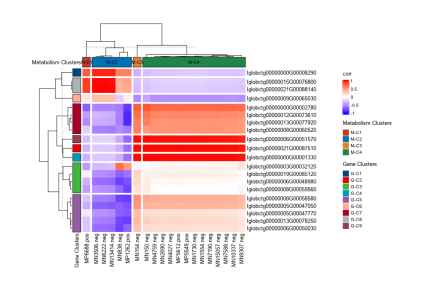


HSW vs YM RS vs HQ WDF vs DS


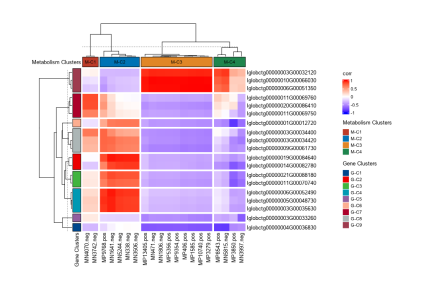

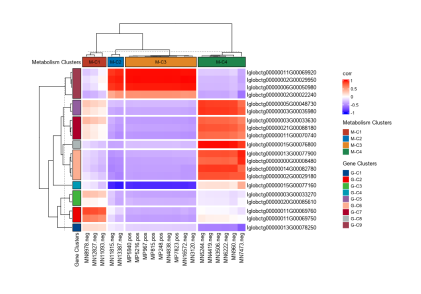

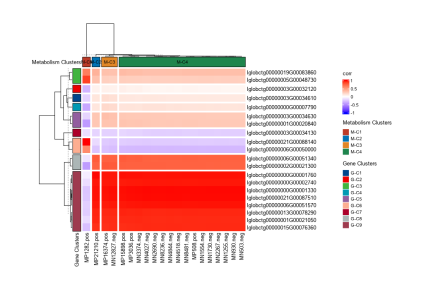


WDF vs HQ WDF vs RS YM vs DS


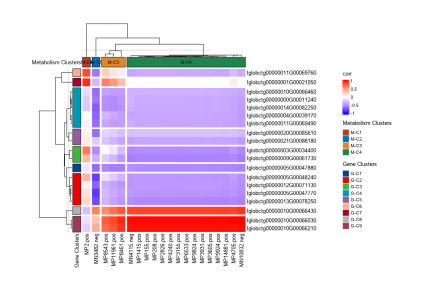

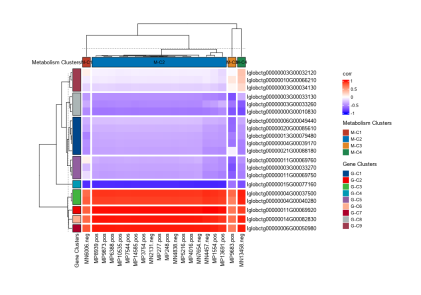

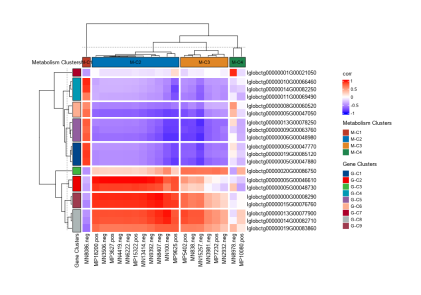


YM vs HQ YM vs RS YM vs WDF

FIGURE S7

Correlation heatmap of *I. glomeratus.* Main area of the heatmap: The horizontal axis represents genes, and the vertical axis represents metabolites; the darker the color, the higher the positive correlation between the gene and the metabolite; conversely, the bluer the color, the stronger the negative correlation between the gene and the metabolite; the asterisk indicates a significant p-value less than 0.05. Metabolite clustering tree: Clustering situation of metabolites; Genetic clustering tree: Genetic clustering situation; Gene Clusters: Gene clustering classification labels. Genes with similar correlation values are clustered together as one cluster. Regulation: Information on the upregulation and downregulation of genes and metabolites in each comparison group.


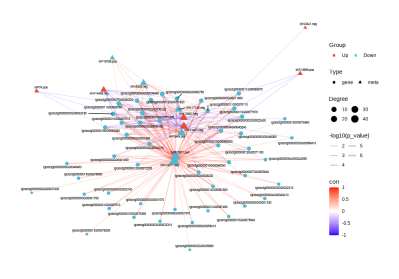

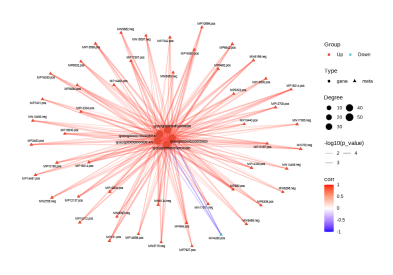

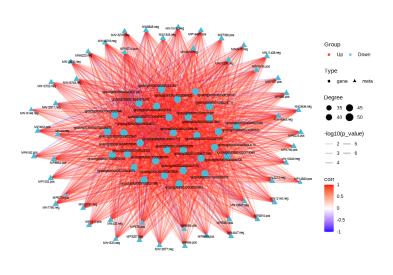


DS vs HQ DS vs RS HSW vs DS


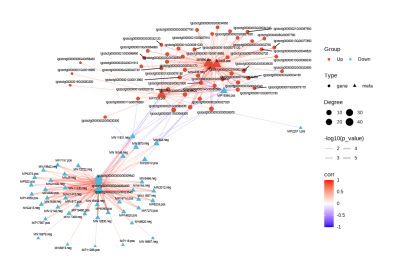

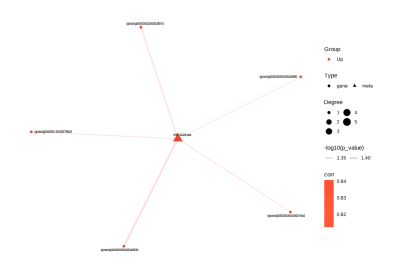

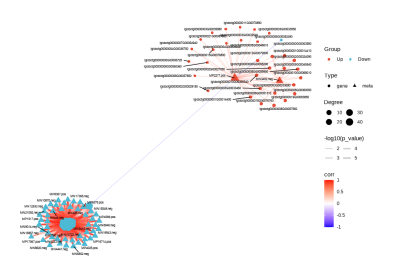


HSW vs HQ HSW vs RS HSW vs WDF


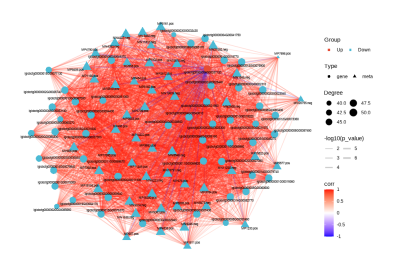

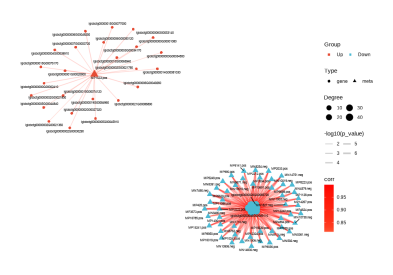

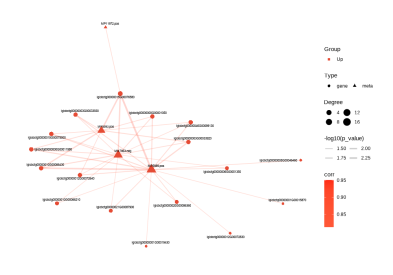


HSW vs YM RS vs HQ WDF vs HQ


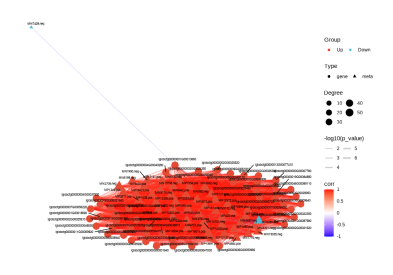

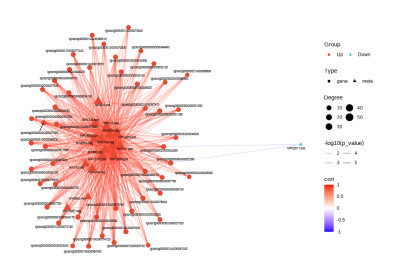

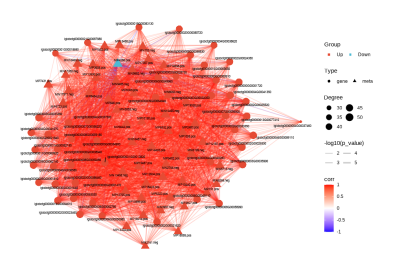


WDF vs RS YM vs HQ YM vs RS


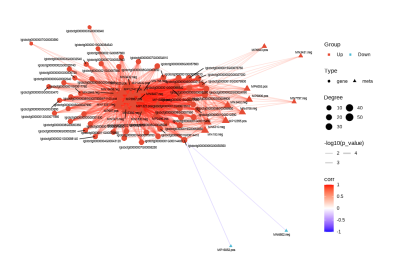


YM vs WDF

FIGURE S8

Correlation network diagram of *I. glomeratus.* The color of the lines represents the magnitude of the correlation coefficient, the thickness represents the p-value size, and the shape of the nodes represents the genes and metabolites.


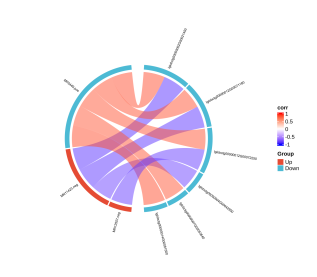

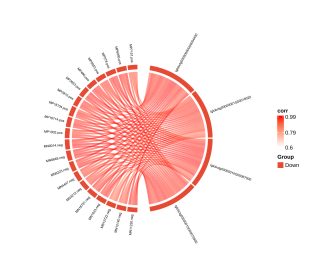

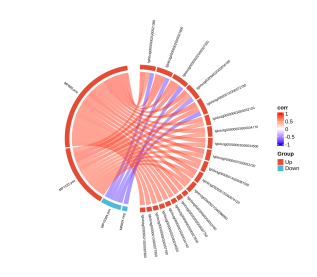

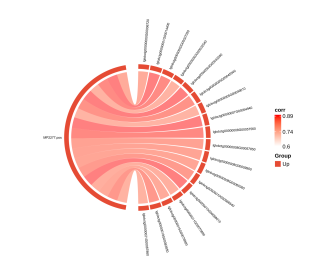


DS vs HQ HSW vs DS HSW vs HQ HSW vs WDF


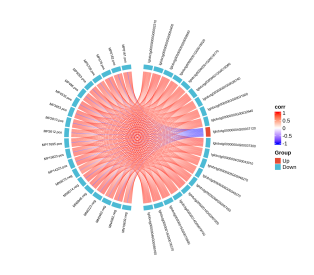

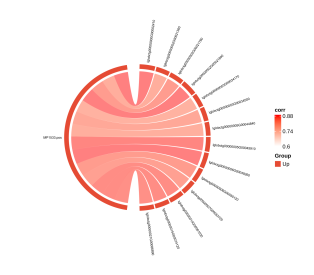

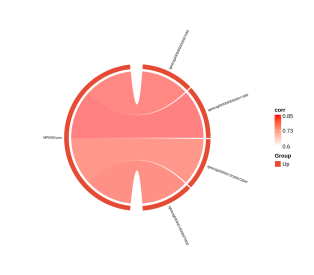

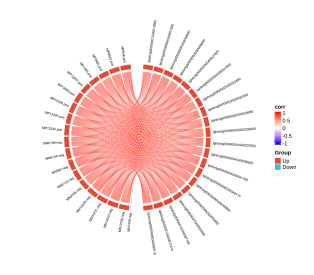


HSW vs YM RS vs HQ WDF vs HQ WDF vs RS


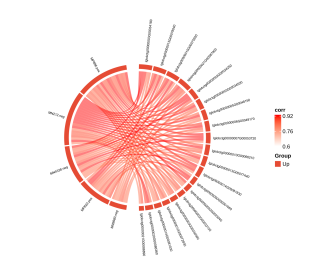

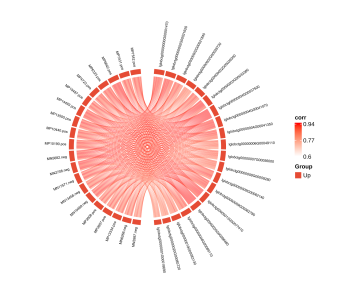

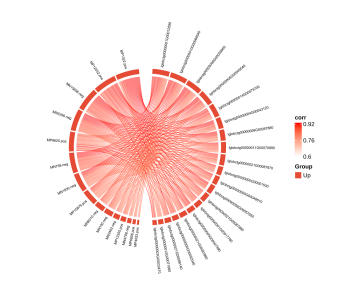


YM vs HQ YM vs RS YM vs WDF

FIGURE S9

Correlation string graph of *I. glomeratus.* On the left is the name of the metabolite, and on the right is the name of the gene. Red indicates a positive correlation, and blue indicates a negative correlation.


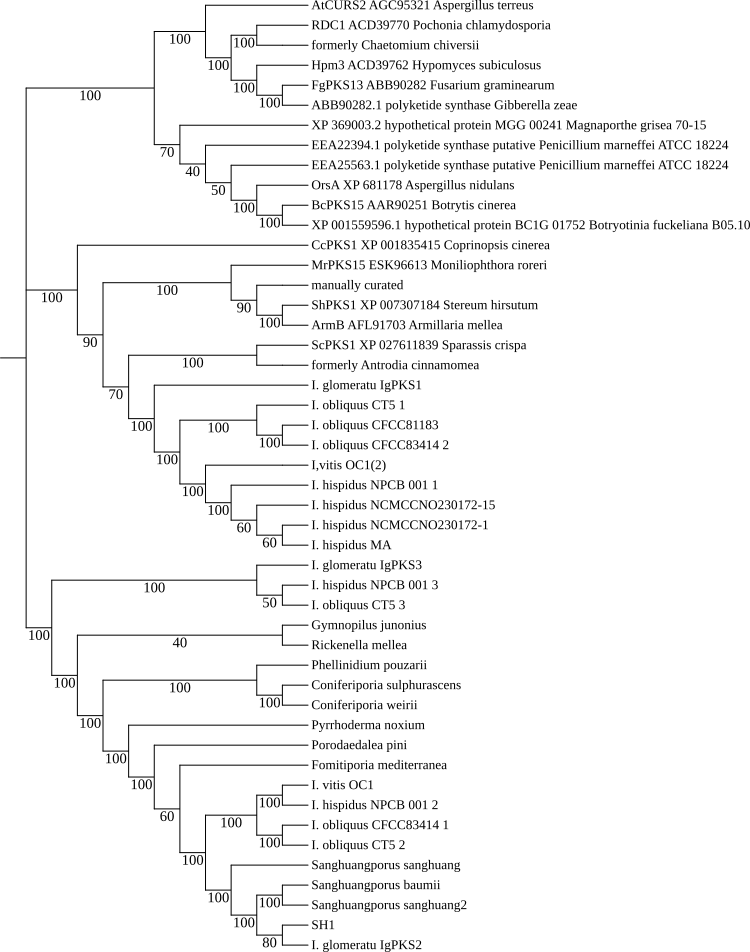


FIGURE S10

Construction of a PKS phylogenetic tree for the fungus *I. glomeratu*s.


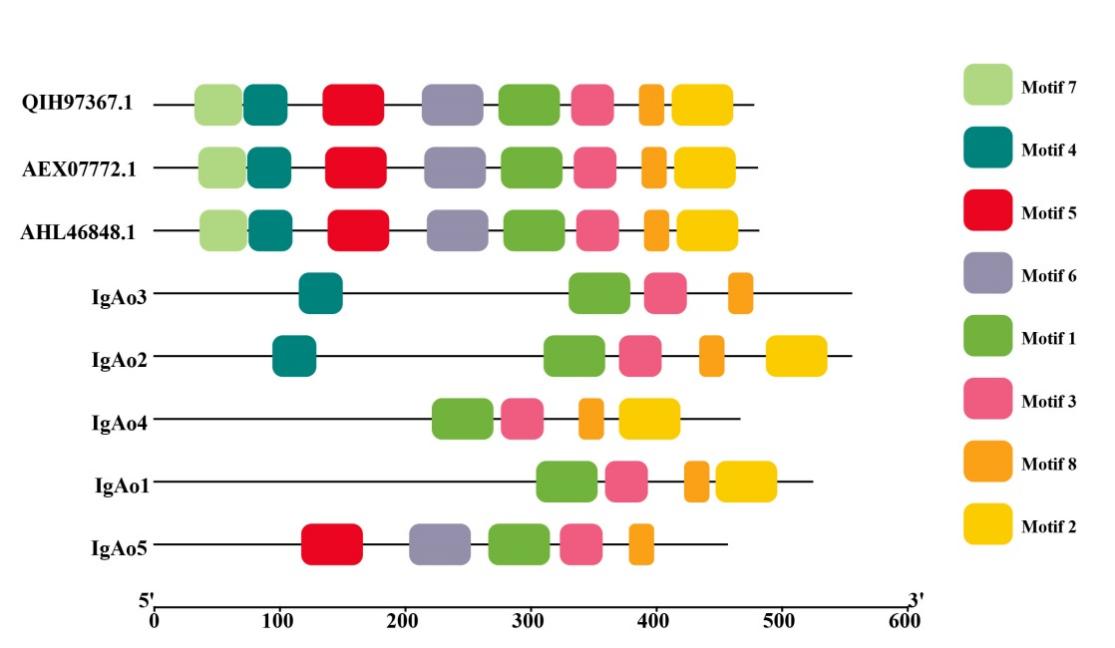


FIGURE S11

Conserved motif analysis of the betulinic acid synthesis pathway gene *IgAo* in *I. glomeratus*.


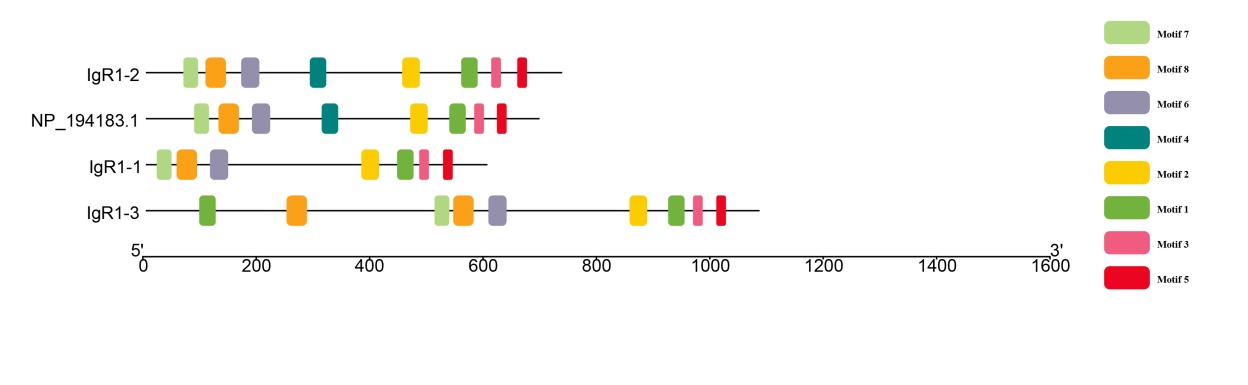


FIGURE S12

Conserved motif analysis of the betulinic acid synthesis pathway gene *IgR1* in *I. glomeratus*.


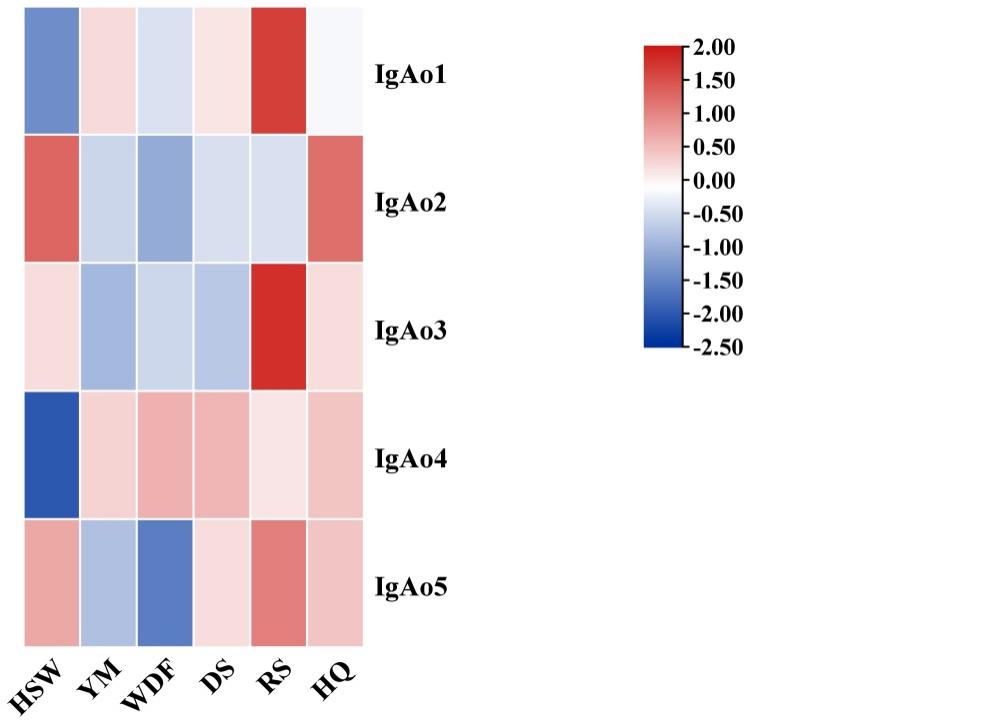


FIGURE S13

Heatmap of the expression of the betulinic acid synthesis pathway gene *IgAo* in *I. glomeratus* under different culture substrates. *IgAo* was obtained by native blast. The horizontal axis indicates different culture substrates and the vertical axis indicates gene expression. Expression levels are color-coded, with red and blue indicating high and low expression, respectively.


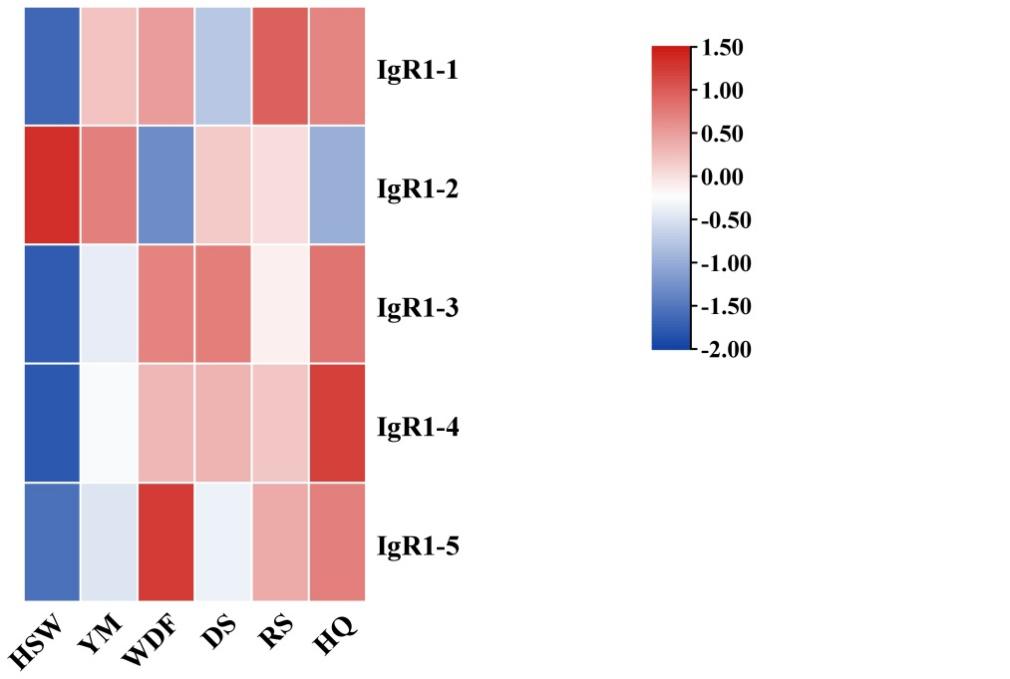


FIGURE S14

Heatmap of the expression of the betulinic acid synthesis pathway gene *IgR1* in *I. glomeratus* under different culture substrates. *IgR1* was obtained by native blast. The horizontal axis indicates different culture substrates and the vertical axis indicates gene expression. Expression levels are color-coded, with red and blue indicating high and low expression, respectively.

TABLE S1 Primer list of genes for qRT-PCR.

| Primer Name | Sequence (5'-3') |
| --- | --- |
| IgPKS1F | TAGCGAAAGAATGCATGCTT |
| IgPKS1R | ACTGGAAGTGATAGAACGTT |
| IgPKS2F | CATCACGCGCGAGCTGTATG |
| IgPKS2R | CGATATCGTATTCGGTGCCG |
| IgTPS3F | ATGGCTCGAGAGATGGCTGA |
| IgTPS3R | CTTGTGTCTCGTCTTAGCGT |
| IgTPS9F | ATCGAAGACGACATGACGAT |
| IgTPS9R | CTTATCGCAGATGTCGAGGA |
| IgTPS11F | GCTGTCCATCCTCAATACAT |
| IgTPS11R | GACAGACATTATTGCGCTGA |
| IgMYB3F | GCTTAGGCTGTCGCAACAGA |
| IgMYB3R | ATCGTGGTTTGCTTCAGAAC |
| IgHMG1F | ATTGACAAGTCCAAGTCTGT |
| IgHMG1R | TAGAAGTCGTACGTGTTCGC |
| IgAnkyrin6F | GTACAGCGTGATGTCAAGGT |
| IgAnkyrin6R | AGCTCCGTCGATATTGTCTA |
| IgbHLH2F | CAACAGGACCCACAGCTTAT |
| IgbHLH2R | TGCGGTAGAGGTGGTGCCTG |
| Tubulin alphaF | TATGAGAATGGATAGTCTTG |
| Tubulin alphaR | TCTATGAGGTATAGATACGC |

**TABLE S2** Genomic characterization of seven strains of the genus *Inonotus.*

| Strains | Accession Number | Total Length(Mb) | caffold | GC Content (%) |
| --- | --- | --- | --- | --- |
| Inonotus obliquus（CFCC83414） | GCA_032164295.1 | 36.1 | 32 | 47.5 |
| Inonotus hispidus（NCMCCNO230172） | GCA_037014295.1 | 37.8 | 28 | 48.5 |
| Inonotus obliquus（CT5） | GCA_023101745.1 | 38.1 | 31 | 47.5 |
| Inonotus hispidus（MA） | GCA_031471775.1 | 34.1 | 24 | 48.5 |
| Inonotus hispidus（Wu） | GCA_047716215.1 | 34.6 | 41 | 48 |
| Inonotus hispidus（NPCB-001） | GCA_024712875.1 | 34 | 17 | 48.5 |
| Inonotus vitis（OC1） | GCA_041146215.1 | 35.2 | 54 | 48.5 |

**TABLE S3 The identity and similarity between betulinic acid biosynthesis enzymes of** *I. glomeratus* **and** *S. baumii***.**

| Gene name | *Sanghuangporus baumii* | *Inonotus glomeratus* | Length | Identity | Similarity | Gaps | Score |
| --- | --- | --- | --- | --- | --- | --- | --- |
| AACT | OCB87509.1 | Iglobctg00000002G00025510 | 420 | 99.50% | 99.50% | 0% | 2072 |
| HMGS | OCB86311.1 | Iglobctg00000003G00031830 | 495 | 99.40% | 99.60% | 0% | 2565 |
| HMGR | OCB83928.1 | Iglobctg00000010G00068430 | 1389 | 99.40% | 99.80% | 0% | 6938 |
| MVD | OCB90355.1 | Iglobctg00000004G00036950 | 402 | 99.30% | 99.30% | 0% | 2034 |
| IDI | OCB86091.1 | Iglobctg00000004G00037830 | 297 | 83.80% | 83.80% | 14.80% | 1249.5 |
| FPPS | OCB92206.1 | Iglobctg00000000G00007560 | 353 | 100% | 100% | 0% | 1849 |
| SQS | OCB89274.1 | Iglobctg00000015G00073960 | 489 | 99.80% | 100% | 0% | 2548 |
| SES | APY26400.1 | Iglobctg00000003G00032360 | 483 | 97.50% | 98.10% | 0% | 2479 |
| LS | QPP12406.1 | Iglobctg00000009G00063780 | 742 | 84.60% | 86.70% | 9.00% | 3325.5 |

Note: Sequence alignment was carried out with the EMBOSS Water program (<https://www.ebi.ac.uk/jdispatcher/psa/emboss_water>).

**TABLE S4 The putative CrAo and ATR1 genes in betulinic acid biosynthesis of *I. glomeratus*.**

| Gene | Accession | Source |
| --- | --- | --- |
| IgAo1 | Iglobctg00000006G00049100 | This study |
| IgAo2 | Iglobctg00000005G00047190 | This study |
| IgAo3 | Iglobctg00000002G00021960 | This study |
| IgAo4 | Iglobctg00000000G00002450 | This study |
| IgAo5 | Iglobctg00000000G00008570 | This study |
| IgR1-1 | Iglobctg00000008G00060650 | This study |
| IgR1-2 | Iglobctg00000014G00080000 | This study |
| IgR1-3 | Iglobctg00000000G00011920 | This study |
| CYP01 | QIH97367.1 | Salvia rosmarinus |
| cytochrome P450 | AEX07772.1 | Catharanthus roseus |
| Cytochrome P450,partial | AHL46848.1 | Betula platyphylla |
| P450 reductase 1 | NP_194183.1 | Arabidopsis thaliana |
